# Recent Advancements and Perspectives of Low-Dimensional Halide Perovskites for Visual Perception and Optoelectronic Applications

**DOI:** 10.1007/s40820-025-01823-z

**Published:** 2025-08-26

**Authors:** Humaira Rafique, Ghulam Abbas, Manuel J. Mendes, Pedro Barquinha, Rodrigo Martins, Elvira Fortunato, Hugo Águas, Santanu Jana

**Affiliations:** i3N/CENIMAT, Department of Materials Science, NOVA School of Science and Technology and CEMOP/UNINOVA, Campus de Caparica, 2829-516 Caparica, Portugal

**Keywords:** Low-dimensional perovskites, Light-emitting diodes, Photodetectors, Phototransistors, Photovoltaics

## Abstract

Low-dimensional (LD) halide perovskites have attracted considerable attention due to their distinctive structures and exceptional optoelectronic properties, including high absorption coefficients, extended charge carrier diffusion lengths, suppressed non-radiative recombination rates, and intense photoluminescence. A key advantage of LD perovskites is the tunability of their optical and electronic properties through the precise optimization of their structural arrangements and dimensionality. This review systematically examines recent progress in the synthesis and optoelectronic characterizations of LD perovskites, focusing on their structural, optical, and photophysical properties that underpin their versatility in diverse applications. The review further summarizes advancements in LD perovskite-based devices, including resistive memory, artificial synapses, photodetectors, light-emitting diodes, and solar cells. Finally, the challenges associated with stability, scalability, and integration, as well as future prospects, are discussed, emphasizing the potential of LD perovskites to drive breakthroughs in device efficiency and industrial applicability.

## Highlights

- This review uniquely bridges the relationship between 0D, 1D, and 2D structural motifs of halide perovskites and their distinct optoelectronic properties; such as photoluminescence, charge transport, and excitonic behavior and how these impact performance across various devices (e.g., LEDs, photodetectors, synapses). This dimensional-property-functionality mapping is not extensively covered in previous reviews.
- Unlike many earlier reviews focused solely on photovoltaics or LEDs, this article expands into emerging fields like artificial synapses and visual perception-related electronics, offering insights into how low-dimensional perovskites could enable next-generation neuromorphic and intelligent sensing systems.
- The review doesn't just summarize the field it also critically evaluates current limitations in scalability, environmental stability, and device integration, and provides future directions to overcome these, particularly through material design and interfacial engineering, making it highly relevant for guiding industrial research.

## Graphical abstract

**Figure:**
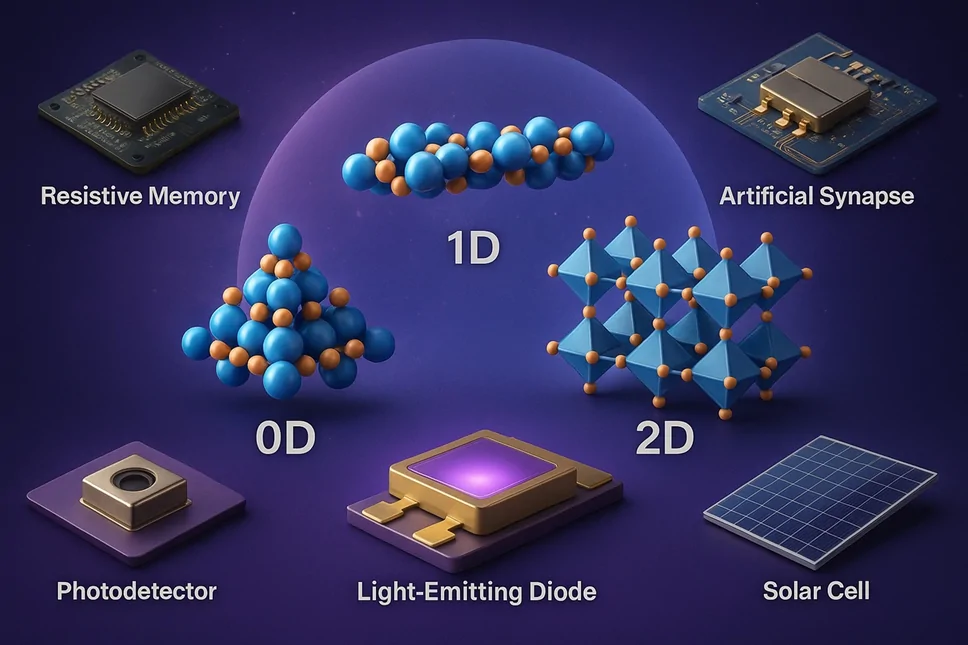

## Introduction

Dimensionality impacts the electron–matter and light–matter interactions, which affect the characteristics and functionalities of semiconducting nanostructured materials and devices [1]. Over the last three decades, significant advances have been made in the synthesis, processing, and characterization of nanocrystals, one-dimensional (1D) nanowires (NWs), and two-dimensional (2D) quantum wells in typical inorganic semiconductor nanostructures (for example, elemental III–V and metal chalcogenide) [2]. The main goal of these studies is to figure out how dimensions, size, architecture, and compositions impact the characteristics of nanostructures so that new or improved properties and applications may be developed [3]. With critical semiconductor nanostructures, many device systems, including integrated optoelectronic and photonic devices, biosensors, energy conversion and storage devices, could be potentially revolutionized [4, 5]. Among various semiconductor materials, halide perovskites have developed as an important semiconductor class for high-performance solution-processed optoelectronics including lasers [6, 7], photovoltaics [8, 9], and light-emitting diodes (LEDs) [10–12].

The most prominent and unique properties are their high electron/hole mobility and absorption factor, long-range ambipolar transport of charge, high dielectric constant including low exciton binding energy and good defect tolerance. The performance of three-dimensional (3D) hybrid photovoltaics has been boosted with maximum power conversion efficiencies (PCEs), as compared with other established technologies [13]. However, 3D hybrid organic–inorganic perovskite solar cells still lack commercialization due to their unstable behavior [14]. Compared to 3D perovskite materials, the low-dimensional (LD)-hybrid perovskites show better stability against moisture, higher photoluminescence, tunable bandgaps, and lower trap densities, leading to better stable and efficient optoelectronic devices [15]. In comparison with bulk perovskites, LD perovskites have favorable optical and electrical properties, including strong anisotropic absorption as well as emission, higher photoluminescence quantum yields (PLQY), adjustable bandgaps, and high binding energies, and thus could provide a variety of thrilling and potentially unique opportunities. Another advantage is the ease of fabrication of LD perovskite materials via low-cost solution processing and at ambient temperatures in several (0D, 1D, and 2D) forms without a highly sophisticated environment.

In the last decades, LD perovskites have been getting tremendous attention due to their extraordinary properties [16]. The crystal sizes, compositions, and shapes play a dominant role in tuning the density of their electronic states and band gaps, making them artificial atoms. For example, in 2D perovskites, ABX_3_ possesses the ability of tenable “A” cation length for the interlayer architecture [17]. This property leads to applying 2D perovskite crystal structures with desirable novel optoelectronic properties. Also, the versatile structures of LD perovskites with better quantum confinement enable them to tune their optoelectronic properties using compositional engineering. Therefore, LD perovskite materials have become the most prominent and efficient candidates for a new generation of several applications based on optoelectronics, including light-emitting diodes [18], phosphorescent material [19], low power transistors [20, 21], exceptionally efficient photodetectors [22, 23], lasers [24], solar cells [25], and printable devices [26, 27].

**Fig. 1:**
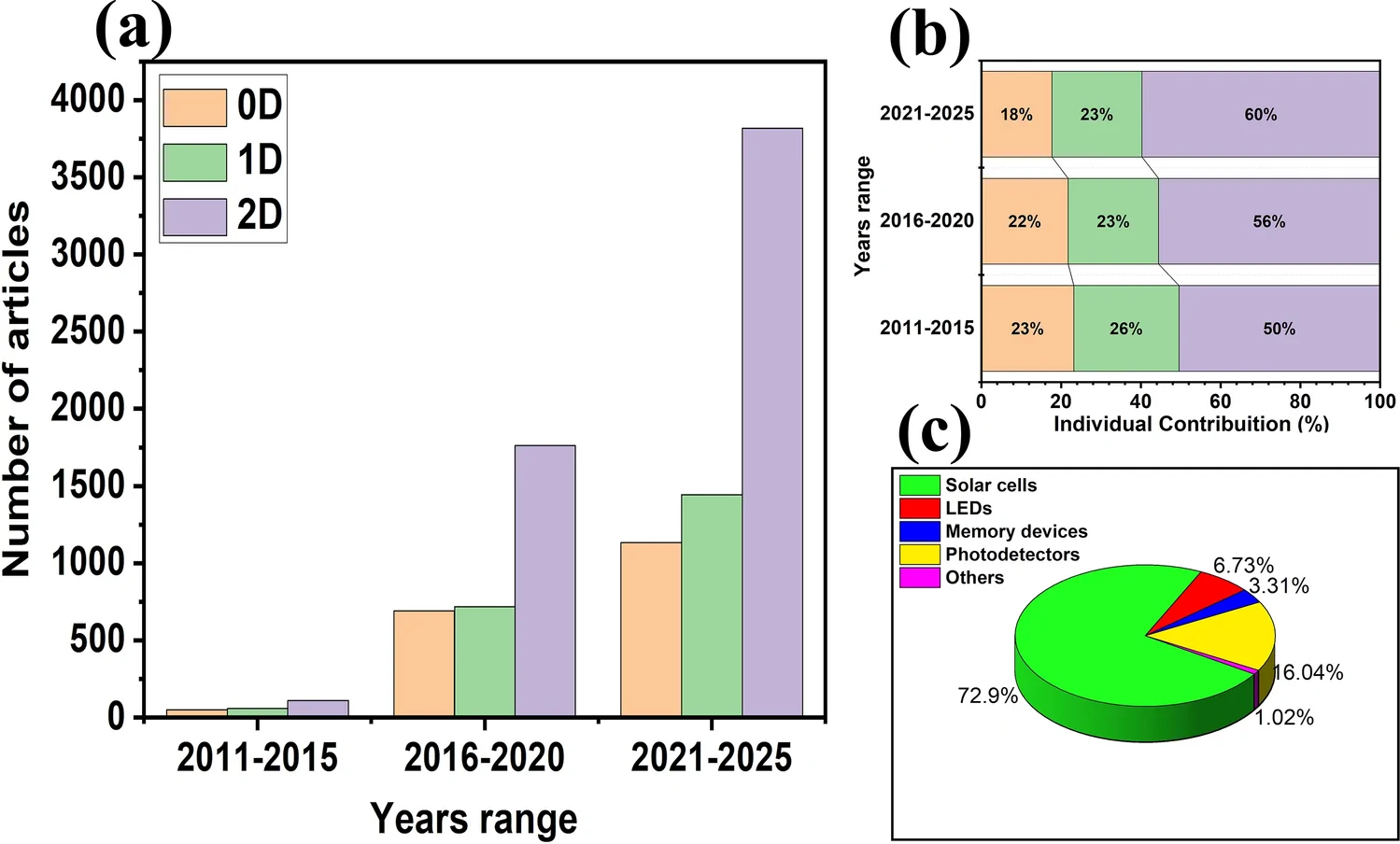
**a** Number of publications on low-dimensional (0D, 1D, 2D) perovskites from 2011 to 2025 across three-time intervals. **b** Contributions from each low-dimensional perovskite group to the total number of articles. **c** Distribution of publications for different applications of low-dimensional perovskites during the 2011–2025 period

In parallel with market growth, research and development efforts in flexible electronics have expanded significantly in recent years, as evidenced by the increasing number of publications in the field of flexible and wearable electronics. We analyzed the number of articles published during three distinct time intervals: 2011–2015, 2016–2020, and 2021–2025. Figure 1a illustrates the number of publications on 0D, 1D, and 2D perovskites during each period. A more detailed analysis is provided in Fig. 1b, which highlights the individual contributions of 0D, 1D, and 2D perovskites to the total publications. The percentage of publications focused on 0D materials increased from 18% to 23%, while 2D perovskite research consistently accounted for more than 50% of the total publications on low-dimensional perovskite materials. To gain further insights, we combined the results from 2011 to 2025 to examine the total number of articles across different applications. The data, represented as a pie chart in Fig. 1c, reveal that the majority of publications (72.9%) are focused on solar cells, followed by photodetectors (16%) and light-emitting diodes (LEDs) (approximately 7%). These data were sourced from the Web of Science. In this review article, we briefly discuss the classification, synthesis, and properties of LD perovskites. In addition, the precise structural, optical, and photophysical features are also illustrated that distinguish them as a leading material in the perovskite field. We also summarise the state-of-the-art applications, main challenges, and emerging prospects for LD perovskite-based memory devices, synapse, photodetectors, LEDs, and solar cells with a deep focus toward enhanced device performance and efficiency for industrial applications.

### LD Perovskites Dimensional Classification

The classification of LD perovskites based on their dimensions includes zero-, one-, and two-dimensional perovskites. Zero-dimensional materials include quantum dots, nanoparticles, and nanoclusters, one-dimensional include nanorods, nanowires, and nanotubes, and three-dimensional includes nanoplates, nanolayers, and nanofilms.

**Fig. 2:**
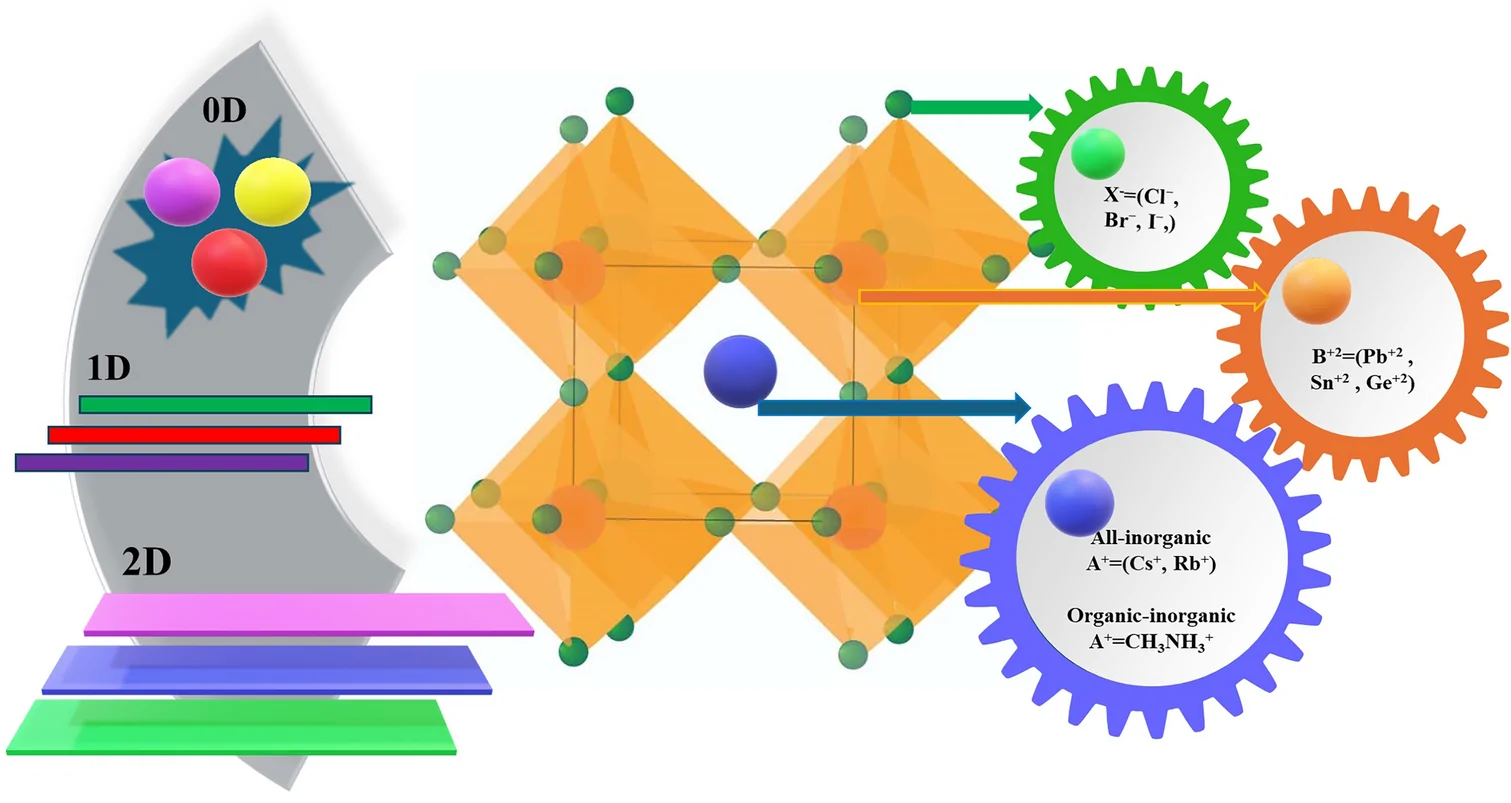
Classification of LD perovskites based on dimensionality (left) and structural diagram of halide perovskites (ABX_3_). Central A site atom (organic or inorganic) determines the nature of all-inorganic or hybrid organic–inorganic perovskites (right)

**Fig. 3:**
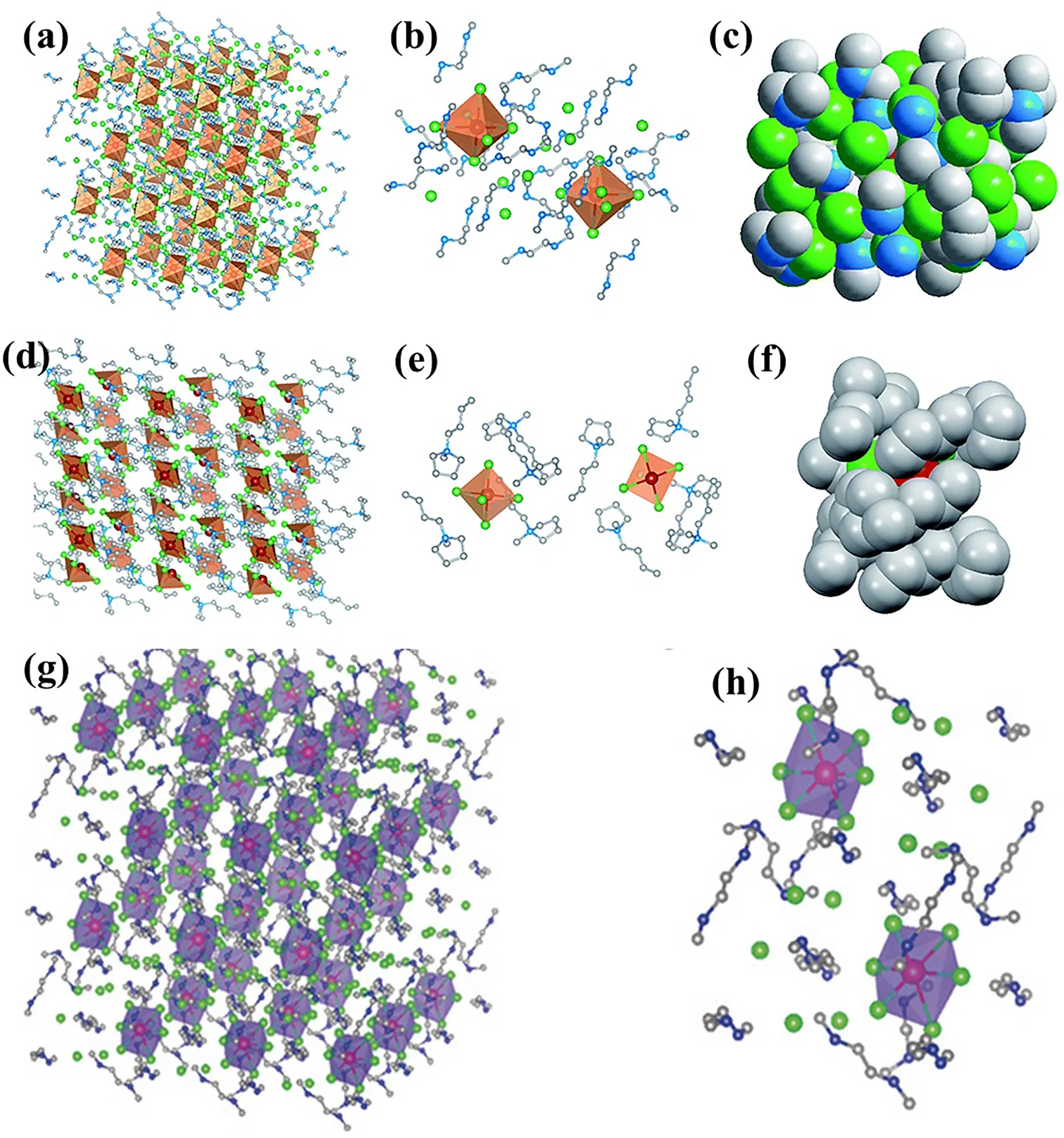
Single-crystal structures and isolation of 0D organic metal halide hybrids. **a** Crystal structure of (C_4_N_2_H_14_Br)_4_SnBr_6_ with SnBr_6_^4−^ octahedra (orange) and organic ligands (C/N: gray/blue; Br: green). **b** Two isolated SnBr_6_^4−^ octahedra surrounded by C_4_N_2_H_14_Br^+^ ligands. **c** Space-filling model of a single SnBr_6_^4−^ octahedron fully encapsulated by organic ligands. **d** Crystal structure of (C_9_NH_20_)_2_SbCl_5_ featuring SbCl_5_^2−^ quadrangular pyramids (orange) and organic ligands (C/N: gray/blue; Cl: green). **e** Two isolated SbCl_5_^2−^ quadrangular pyramids enclosed by C_9_NH_20_^+^ ligands. **f** Space-filling model of an individual SbCl_5_.^2−^ quadrangular pyramid shielded by organic ligands [35]. **g** Crystal structure of 0D Sn bromide perovskite (C_4_N_2_H_14_Br)_4_SnBr_6_ and **h** 0D Sn bromide perovskite unit cell [36]

Figure 2 shows the structural classification of LD perovskites based on dimensionality, which includes 0D, 1D, and 2D perovskites. The cubic structural diagram of halide perovskites with ABX_3_ structure is shown in Fig. 2(left). This structure shows the similarities and differences between all-inorganic and organic–inorganic hybrid perovskites. The main dissimilarity which differentiates inorganic from hybrid perovskites is the central A site atom (organic or inorganic), which determines the nature of all-inorganic or hybrid perovskites. Commonly used A, B, and X ions are mentioned in green, orange, and blue colors (Fig. 3).

#### Zero-dimensional Perovskite Materials

0D perovskites have specific structural and optoelectronic properties. Perovskite quantum dots (PQDs) with size less than its Bohr radii in all three dimensions are considered to be the example of 0D materials [28]. The small sizes result in electron confinement effect and hole wavefunctions [29]. Hence, one can observe that electronic excitations are touching the nanocrystal boundaries and showing better physical, chemical, and optoelectronic properties. These perovskite QDs possess discrete band gaps, and by adjusting the QD sizes, the band gaps may be readily adjusted [30]. Reducing QD sizes may increase the band gap by 1/R^2^ factor, and here, R represents the radius of QDs. The quantum confinement effect enables these PQDs to show narrow emission line widths and a maximum PL quantum yield toward 100% [31]. These prominent characteristics can be utilized in practical applications of PQDs based optoelectronic devices. Several synthesis methods have been reported for perovskite quantum dots, such as the template-assisted method [32], hot injection method [33], and ligand-assisted method [34].

#### One-dimensional Perovskite Materials

One-dimensional perovskites are widely investigated for realizing novel optoelectronic performances, due to their superior and fascinating optical properties featuring a high quantum efficiency and a significant surface-to-volume ratio, anisotropic geometry, and better charge confinement compared with bulk materials [38, 39]. Superior crystalline structure and tremendous properties make 1D perovskites one of the most reliable candidates for the next-generation perovskite-based photonic and electronic devices. 1D perovskites possess well-defined crystal structures, unique 1D enclosed surfaces, higher stability, and better film morphology.

**Fig. 4:**
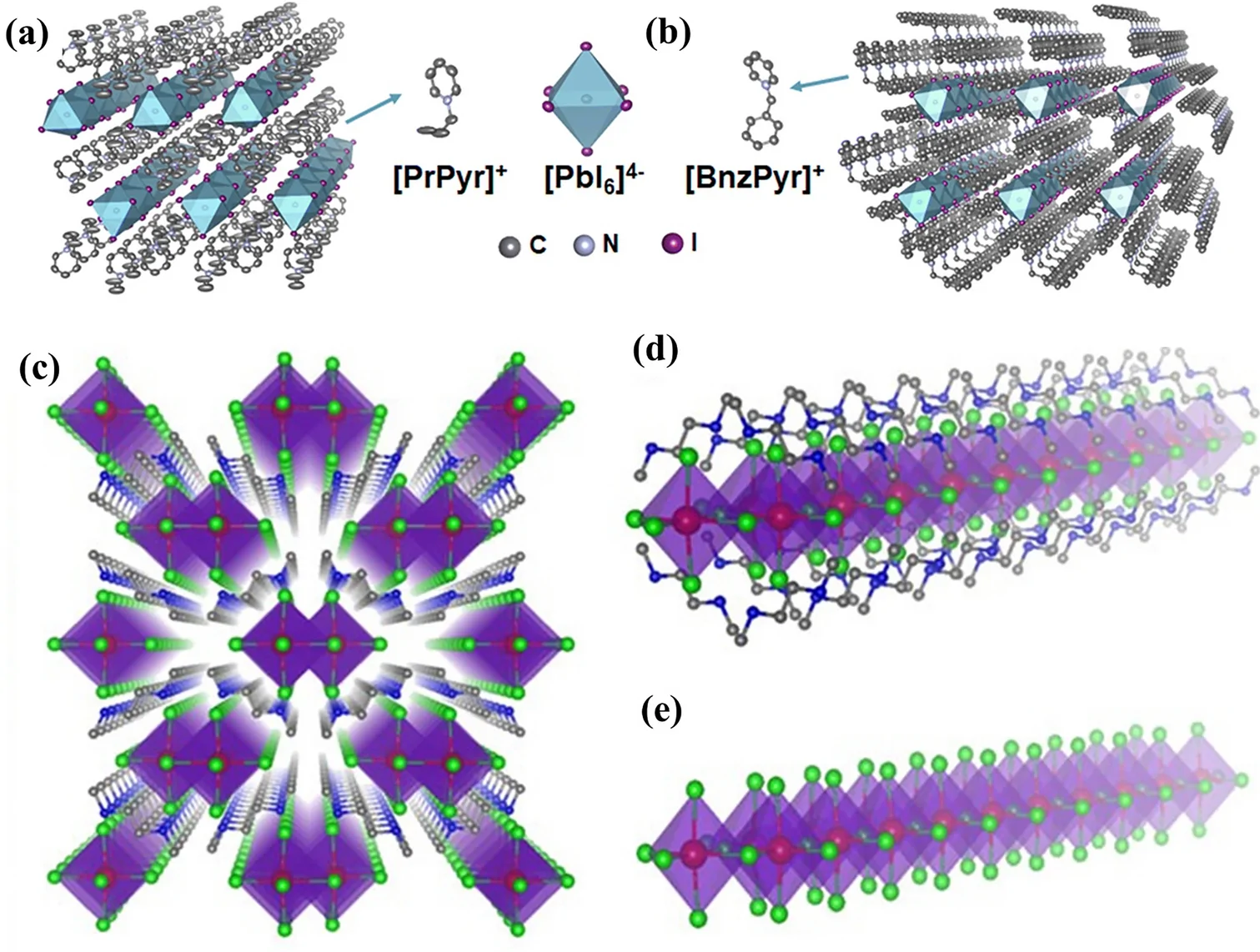
Structure of 1D halide perovskites. Single crystal structures of **a** 1D lead iodide hybrids (PrPyr)[PbI_3_] and **b** (BnzPyr)[PbI_3_]. Here C, N, and I atoms are represented by gray, blue, and purple spheroids, respectively, while the [PbI_6_]^−4^ coordination sphere represented by cyan octahedron. Insets show the molecular structures of PrPyr^+^ and BnzPyr.^+^ cations [46]. **c** Structure of 1D perovskite C_4_N_2_H_14_PbBr_4_. **d** Individual PbBr quantum wire wrapped by the organic cations and **e** PbBr quantum wire with edge-sharing octahedrons [47]

There are several methods for the preparation of 1D perovskites, such as solution-processable synthesis, template-based, and vacuum synthesis processes [40]. Several LD perovskites-based optoelectronic devices are reported to show remarkable high photosensitivity, better photoelectric conversion efficiency, and high photoluminescence (PL) efficiency [41–43]. However, 1D perovskites devices have low current outputs and small active areas so that they are not easy to realize practical applications. Researchers are working to overcome this issue by assembling highly ordered architectures of 1D perovskites that maintain the intrinsic properties of individual 1D perovskites [39]. Well-ordered and highly aligned arrays can improve the surface uniformity, current output, active area, and reproducibility of the fabricated devices. Highly ordered 1D arrays are attractive for photovoltaic applications because of the superior light trapping, significant broadband antireflection, and enhanced mechanical properties [44, 45]. The development of well-aligned 1D perovskites will create room for the availability of 1D perovskite-based large-scale and highly integrated optoelectronic devices [44]. Figure 4 shows 1D perovskite crystal structure where (a-b) represent the single-crystal structures of (PrPyr)[PbI₃] and (BnzPyr)[PbI₃]. The colors are assigned to each atom as: gray, N: blue, I: purple; [PbI₆]^4^⁻: cyan octahedra. The insets represents organic cations (PrPyr⁺, BnzPyr⁺). Similarly, Fig. 4c shows the 1D perovskite C₄N₂H₁₄PbBr₄ where each atoms is represented by a different color like Pb: red, Br: green, N: blue; [PbBr₆]^4^⁻: purple octahedra). Figure 4d, e shows lead bromide quantum wires. Hydrogen atoms omitted in all structures to make the structure simple and easy to understand.

Researchers are developing different assembly methodologies to fabricate 1D arrays [48]. Common techniques involve confinement, meniscus-assisted, and template-guided self-assembly as well as evaporation-induced self-assembly [49]. Every synthesis assembly method gives unique 1D perovskite crystals with different crystal sizes, shapes, aligned horizontal/vertical architecture, periodic structure, other different surface morphologies. Rather than the impact on morphologies and architectures, the synthesis methods have strong influences on the optoelectronic characteristics including charge carrier mobilities, defect states, light extraction, light path lengths, etc. [50, 51].

#### Two-dimensional Perovskite Materials

**Fig. 5:**
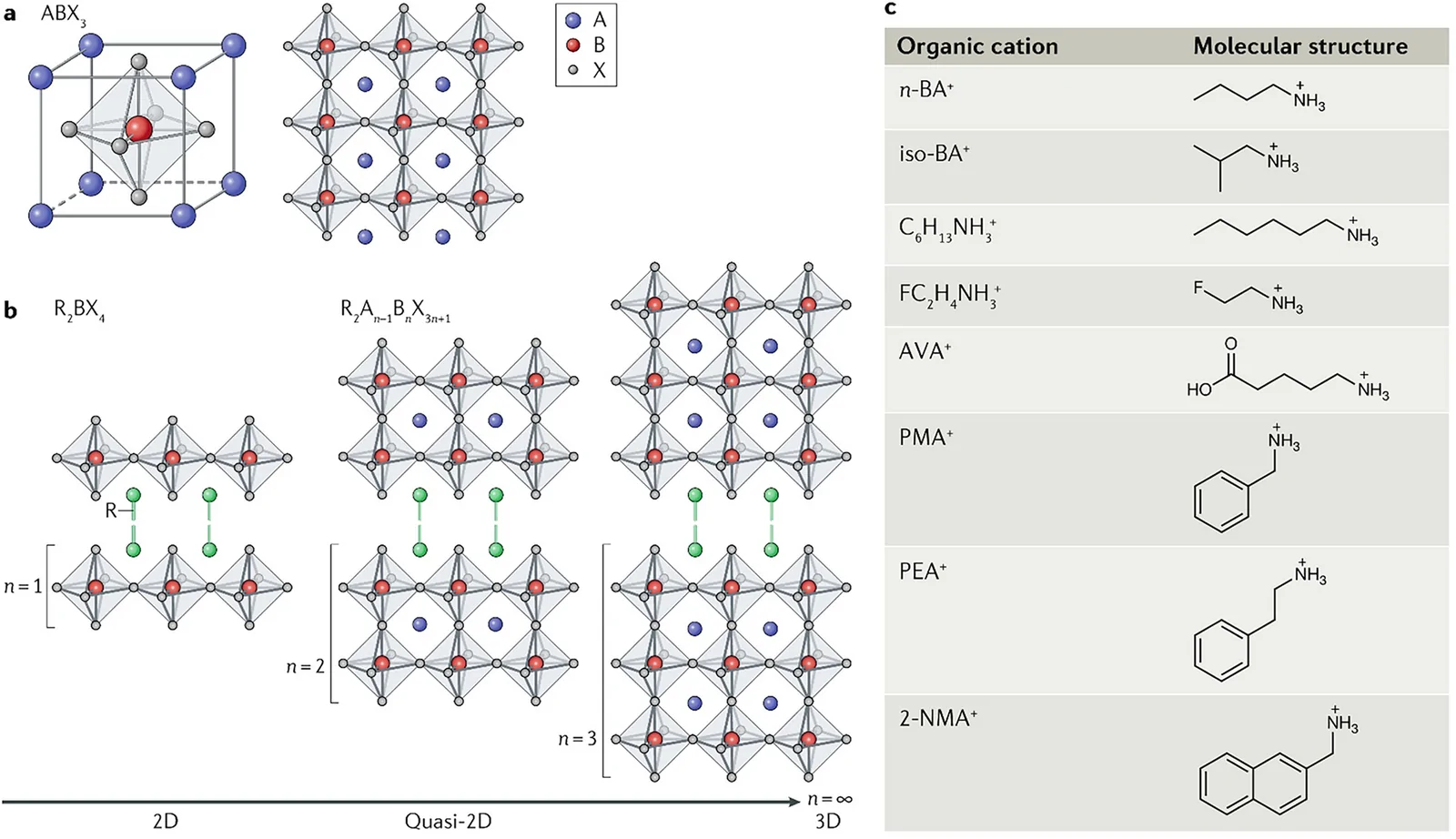
3D and 2D perovskite’s structure. **a** ABX_3_ structure 3D hybrid perovskite, **b** single-, double-, and multiple-layer 2D perovskites. **c** Commonly used organic cations for 2D perovskites [57]

LD perovskites have received significant attention in the last decades [52] toward photonic and other optoelectronic applications [53]. As discussed earlier, the main feature of these materials includes their tunable optoelectronic properties [54] with quantum-sized effect [55, 56]. The simple structures of single, double, and multiple layer 2D perovskites and their 3D architectures, and the commonly used organic cations are shown in Fig. 5.

Figure 5a illustrates 3D hybrid perovskite (ABX_3_), representing corner-sharing [BX_6_]^4−^, where A symbolizes an organic cation, B for a metal cation, and X for a halide. In Fig. 5, low-dimensional perovskite structures have been shown with several perovskite layers (n). Here n = 1 represents pure 2D perovskite with R_2_BX_4_ structure; R represents here a big organic cation. If we increase “n” greater than 1, that is, n > 1, then R_2_A_n−1_B_n_X_3n+1_ structure is formed, which leads to the quasi-2D perovskites. R_2_A_n−1_B_n_X_3n+1_ is the general formula of 2D perovskites (Fig. 5b) [58, 59]. Figure 5c shows commonly used organic cations and their molecular structures in 2D perovskites.

In recent years, 2D perovskites have got a significant interest in the PV market due to their improved stability. 2D perovskites were initiated in the early 1980s. (RNH_3_)_2_MA_n−1_M_n_X_3n+1_ is the most used 2D perovskites general formula. By only altering the value of n, we may regulate the thickness of inorganic layers, which has a direct impact on the characteristics of 2D perovskites [60]. Depending upon the values of n, 2D perovskites can be classified into three main classes, if n = 1, then it is called strict 2D, n = 2–5 belongs to quasi‐2D, and n > 5 represents quasi‐3D perovskites [61]. Most 2D perovskites only consist of the simplest organic cations, such as phenylethyl or butyl ammonium [62]. Now the common interest of researchers is the functional organic cations usage in 2D perovskites so that these functional groups can be utilized to tune the optoelectronic characteristics, leading to better device functionality and efficiency [63, 64].

The flexible structure of 2D perovskites makes plenty of room for materials investigation under compositional engineering, including the choice of R cation. The bulkiest and commonly used R cations are phenylethyl ammonium (PEA^+^) and butylammonium (BA^+^). At the start of the 1990s decade, PEA_2_PbI_4_ having a bandgap around 2.36 eV and relatively superior exciton binding energy about 200 meV (n = 1) is considered to be the most suitable materials to identify the structure as well as optoelectronic properties for the whole 2D perovskites class [65, 66]. This leads to maximum electroluminescence, high PLQY, and significant optical non-linearity. With the addition of smaller cations, like MA^+^ in PEA^+^ for PEA_2_MA_n−1_Pb_n_I_3n+1,_ we can get quasi-2D perovskites [67, 68]. Inorganic sheet thickness is also controlled by changing concentrations of MA^+^ ions in different ratios. The most prominent effect of layer thickness is on the bandgap of material which reduces from 2.36 eV (n = 1) to 1.94 eV (n = ∞) by increasing the value of n and reduces exciton binding energy [69]. It has been noted that the number of inorganic layers in a device can not only be used in tuning the optoelectronic properties but also have a strong influence on crystal orientation and crystal formation of 2D perovskites [70]. There is a direct influence of dimensional variation on binding energy as well as band gap on the final material. An increase in dimensions from 0 to 2D of LD perovskites results in the reduction in binding energy and a band gap. Goldschmidt's tolerance factor-derived size constraints are relaxed and provide the vacancy to incorporate larger organic cations into structure showing the change of exciton binding energy and modulate the bandgap of materials [71, 72].

### Compositional Classification

#### Organic–Inorganic Hybrid Halide Perovskites

The revelation of organic–inorganic hybrid perovskites materials with two-dimensional (2D) structures has enhanced potential in energy harvesting as well as energy storage technologies (e.g., solar cells, LEDs, laser, photodetector, and memory devices). These materials contain metal halide anions and organic cations. A number of hybrid organic–inorganic two-dimensional materials were described by Mitzi et al. in the 1990s with < 110 > metal halide layers [73]. Later, Kagan et al. utilized 2D perovskites as active semiconducting channel layers [20]. A boom in the use of metal halide perovskites was observed after 2009 when Miyasaka et al. firstly reported. These materials serve as photovoltaic (PV) cells' visible light sensitizers with 3.8% power conversion efficiencies (PCE) [74]. The PCE improves dramatically in a decade from 3.8% (2009) to 25% (2020) [75] due to narrow band gap, well-separated electron–hole pairs, maximum mobility, economical and cost-effective fabrication methods. Considering electroluminescent devices, the first room temperature electroluminescent device consisting of organic–inorganic hybrid halide perovskites was fabricated in 2014 [76]. Recently, the structural control for better quantum confinement is of great interest by reducing the dimensionality of bulk 3D to well-arranged 2D levels [77]. Metal halide layers make an important contribution during phase transformation from 3 to 2D by reducing the number of layers with tailorable band gaps and photoluminescence [78]. It is not easy to obtain the 2D perovskite from bulk 3D by mechanical exfoliation or some other techniques adopted for MoS_2_ or graphene. Generally, chemical vapor deposition and solution-based synthesis are adopted for ultra-thin 2D perovskite layers growth. Luckily, PbX_2_ has van der Waals arrangements in MAPbX_3_ which can be availed to make PbI_2_ flakes and further utilized to make MAPnB_3_ by chemical transformation methods [79]. The thickness of resultant perovskites can be controlled from a few to hundreds of layers according to the requirements.

Organic–inorganic hybrid perovskites have attained superior consideration in the optoelectronic industry because of their marvelous properties including tunable bandgap, simple fabrication methods, higher absorption coefficient, which leads to achieve unprecedented PCE of more than 25% [75]. However, the main hindrance in commercialization in organic–inorganic hybrid perovskites is the instability of organic parts under broad exposure to light and relatively higher temperatures. Thermal instability is mainly caused by MA^+^ and FA^+^ cations due to their decomposition at elevated temperatures of 85 and 150 °C for MAPbI_3_ and FAPbI thin films, respectively.

#### Inorganic Halide Perovskites

Inorganic perovskite resolved the thermal instability issue by substituting Cs^+^ ions in the place of organic cations in the precursor solution. CsPbX_3_ was reported in early 1983 but their structure and photo responsive properties were studied in 1958. Recent reports have witnessed that inorganic perovskite possesses better stability and commercialization strength than hybrid perovskites. Considering the synthesis of 2D inorganic perovskites the colloidal synthesis technique is widely adopted [80]. CsPbBr_3_ were synthesized by Bekenstein et al. at higher temperature by directly injecting Cs-oleate solution into PbBr_2_ solution having other solvents including oleylamine, octadecene, and oleic acid [81]. Synthesis temperature plays a dominant role to control the size, thickness, and shape of resulting nanoplates. Precise analysis reveals that experiments conducted at 150 °C mostly produce nanocubes, while low-temperature synthesis (~ 130 °C) could be used to make nanoplates with 20 nm lateral dimension and thickness of ~ 3 nm. The ion exchange method is another synthesis technique for CsPbI_3_ and CsPbCl_3_ nanoplates deposition onto multiple substrates by centrifugal casting for device fabrication [82]. Small lateral sizes ~ 20–300 nm are not sufficient for the assessment of fundamental properties for device fabrication. Shamsi et al. reported rectangular shaped, better lateral sizes from 300 nm up to a few micrometers of ultra-thin CsPbBr_3_ sheets and completely dissolving in oleic acid to present short ligands [83]. Oleic acid has been effectively used as a solvent for Cs-oleate which leads to the formation of nanoplates in a broader temperature range (50 to 150 °C). To achieve sizeable and well-controlled lateral dimension (300 nm to 5 mm) nanoplates, optimum ratios of shorter ligands, octylamine, and octanoic acid can be added into the solution [84].

#### 2D Ruddlesden–Popper (RP) and Dion–Jacobson (DJ) Perovskites

The poor stability of halide perovskites is still a critical issue that needs to be addressed before this low-cost, solution-processable PV technology can be commercialized on a wide scale. Introducing bulky or long-chain monovalent organic cations was previously shown to be a successful method for improving the chemical stability of halide perovskites. Due to better environmental stability, the Ruddlesden − Popper (RP) 2D perovskites got significant attentions from researchers. Commonly single (sometimes multiple) inorganic sheets are sandwiched between organic spacers in 2D perovskites attached by Coulombic forces, such as C_3_H_7_NH_3_^+^ (PA^+^), C_4_H_9_NH_3_^+^ (BA^+^), and C_8_H_9_NH_3_^+^ (PEA^+^), to form 2D RP perovskite phase. As a result, it is exciting to see if customized cation designs might offset the negative impacts of interlayer structures like those found in 2D RP perovskites, allowing for greater stability while limiting carrier transport in solar cells.

**Fig. 6:**
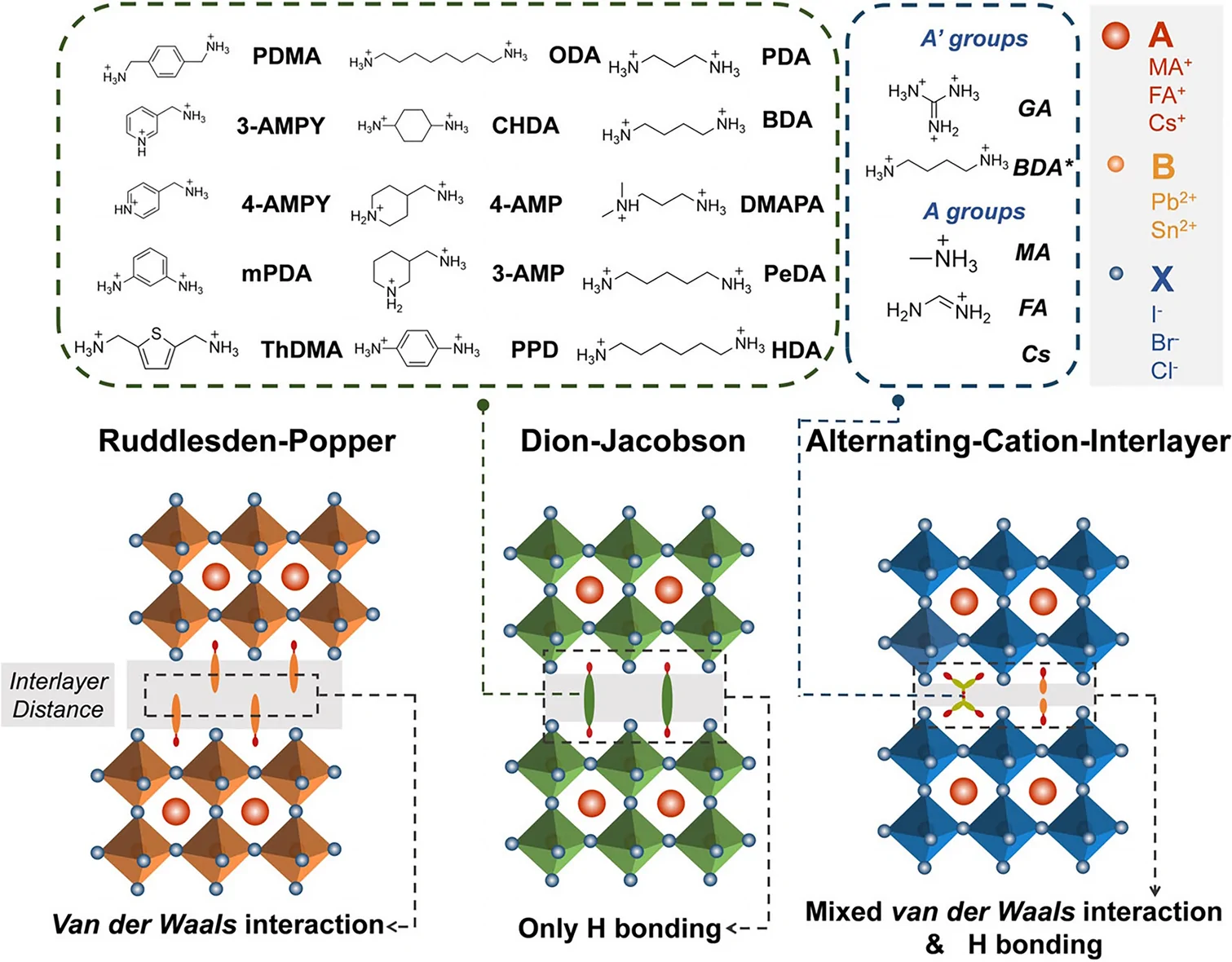
Brief structural comparison between RP, DJ, and ACI phases, with most frequently adopted interlayer cations [86]

Recently, 2D Dion–Jacobson (DJ) perovskites phase with chemical formula (A)_n-1_B_n_X_3n+1_, and alternating cation interlayer (ACI) phases with chemical formula (A′)(A)_n_B_n_X_3n+1_, are attracting a lot of attention from researchers in optoelectronics. The difference of A′ cation makes DJ and ACI phases different. The detailed selection of A, B, and X and structural differences between DJ, RP, and ACI phases are shown in Fig. 6. The chemical formulas of DJ and ACI are a derivative from ABX_3_. 2D DJ phase of perovskites can be fabricated by eliminating van der Waals gaps in RP which leads to better structural strength for photovoltaics [85]. These one-of-a-kind interlayer chemistries favor increased stability while allowing for more carrier hopping/tunneling. As a result, the promise of DJ and ACI LHPs to obtain more optimal device performance in solar cells has been demonstrated. The main distinction is the octahedral configuration in these structures has been shown in the middle panel of Fig. 6. The tightly packed interlayers consisting primarily of modest or small cations favor ACI with GA/MA combinations, resulting in a very low distance of ≈3 Å. There is still an opportunity for further reduced interlayer distances of ACI to be developed.

Better efficiencies of these phases for photovoltaics and structural stability lead to the long-term utilization of fabricated devices by maintaining 95% of initial PCE for 4,000 h at normal air exposure, damp heat (85 °C and 85% RH, 168 h.), and light illumination for 3,000 h.

### Synthesis of LD Perovskites

Understanding growth, as well as assembly mechanisms, is essential to achieve good controllability on the fabrication and improved device performance applications. The preparation route has a significant impact on structural morphologies and performances of the devices based on LD perovskite materials. The feasible synthesis technique to make them available for industrial applications is always desirable. In this section, the solution phase, vapor phase deposition, and templated synthesis have been demonstrated in a controllable and versatile way for the uniform and better geometry LD perovskites nanostructures. Some important factors that significantly affect the film quality including deposition temperature, solvent concentration, substrate temperature, and annealing conditions are also reviewed.

#### Solution Phase Synthesis

**Fig. 7:**
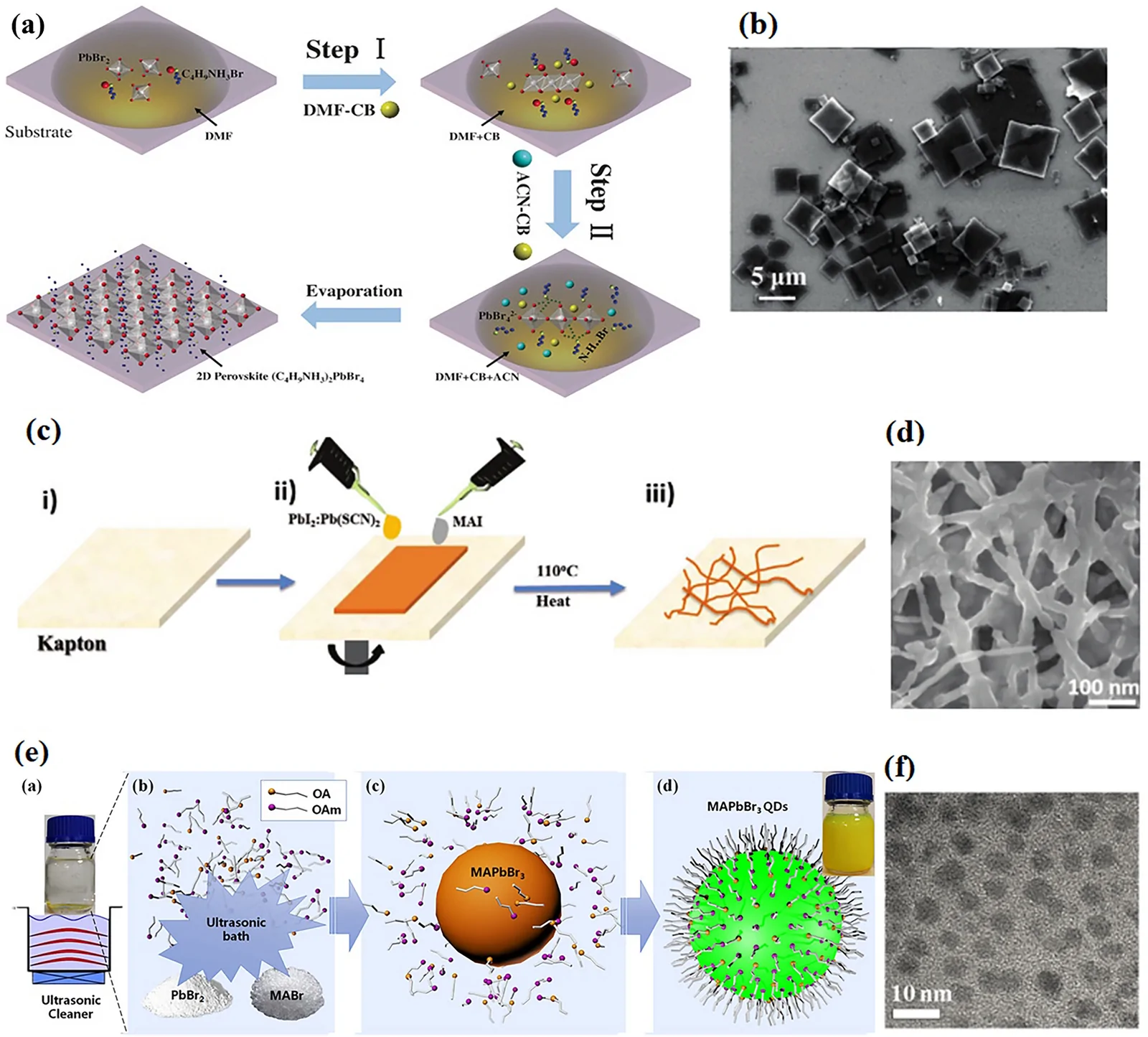
Solution-processable synthesis. **a** Two-step deposition of 2D (C_4_H_9_NH_3_)_2_PbBr_4_ perovskite. **b** Morphological SEM image of 2D (C_4_H_9_NH_3_)_2_PbBr_4_ synthesized at 9.1% DMF ratios [92]. **c** Synthesis schematic of CH_3_NH_3_PbI_3-x_(SCN)_x_ perovskite NWs growth on flexible tape. **d** SEM showing fabricated perovskite nanowires [93]. **e** Schematic showing all states of ultrasonic bath synthesis for CH_3_NH_3_PbBr_3_ perovskite quantum dots. **f** TEM image of synthesized CH_3_NH_3_PbBr_3_ perovskite quantum dots [94]

Several groups are working to attain a few layers of uniform and compact 2D perovskites, high-quality single crystals, and other LD nanostructures by using solution-processable techniques. Solution-based syntheses offer the benefit of allowing for mass manufacturing of colloidal nanostructures for optoelectronic device fabrication with ambient synthesis conditions including low cost and heterolayer deposition feasibility. As a result, solution-based synthesis techniques (both bottom up and top down) are widely used in the fabrication of several nanostructures [79]. Falling a precursor solution on the substrates and then evaporating the solvent resulted in the formation of ultrathin 2D MHP NSs [87, 88]. Another method for assisting the precipitation of 2D MHP nanostructures is to introduce poor solvents to the precursor solution. Zhang et al. used toluene as a poor solvent to grow (PEA)_2_PbI_4_ (PEA = C_8_H_9_NH_3_) NSs. After drop casting, they added toluene to the precursor solution and gradually evaporated the solvents [89]. According to Chen et al., the presence of particular polar solvents in the solution can effectively limit crystal development along the c axis, resulting in ultrathin 2D MHPs once the solvent is evaporated [90]. Yuko et al. used a one-step synthesis method for the growth of 2D (C_3_F_7_CH_2_NH_3_)_2_PbBr_4_ perovskite by using acetone as a poor solvent. They attained the dielectric as well as quantum confinement effects by using this one-step solution deposition technique [91]. Furthermore, Zhai et al. used a two-step solution synthesis technique with the addition of a ternary solvent for the fabrication of large sized 2D (C_4_H_9_NH_3_)_2_PbBr_4_ perovskites. Figure 7a schematically illustrates the synthesis procedure of these 2D (C_4_H_9_NH_3_)_2_PbBr_4_ perovskites. Starting with the dissolution of PbBr_2_ and C_4_H_9_NH_3_Br in DMF solvent and undergoing a two-step deposition, the solution crystallizes into 2D (C_4_H_9_NH_3_)_2_PbBr_4_ perovskite instantly after evaporation [92]. The concentration of perovskite precursor and crystallization temperature were shown to be two important parameters influencing the final morphology of the products. The volume ratio effect of DMF on the morphology of perovskites films was studied and recorded for several concentrations. At a higher ratio of DMF (33.3%), the precursor concentration is very small, and randomly shaped perovskites were found. By making the solution more concentrated and decreasing the ratio of DMF up to 9.1%, the fine perovskites cubes were achieved with uniform sizes as shown in Fig. 7b. The concentration of DMF has significant effect on the nucleation density and size of 2D (C_4_H_9_NH_3_)_2_PbBr_4_, by reducing the DMF ratio results in the increase in nucleation density of perovskites and also enhancements in the sizes of the perovskites significantly. Surprisingly the shapes and random morphologies of perovskites were completely transformed into well-aligned cubes. This evolution can be explained by the supersaturation phenomenon, which dominates the development mode of perovskites and transforms them into a cubic shape with enhanced nucleation density. Cloutier and co-workers used solution-processable synthesis technique to fabricate 1D hybrid perovskite nanowires and utilized them to make flexible photodetector [93]. The schematic for the production and deposition of perovskite nanowires on a flexible Kapton substrate using the spin-coating approach is shown in Fig. 7c, the fabricated nanowires are highly dense and cover the entire surface of the flexible substrate. Figure 7d shows a high-magnification SEM image of a CH_3_NH_3_PbI_3-x_(SCN)_x_ perovskite nanowires network deposited on Kapton substrates. Like 2D and 1D perovskites, the 0D perovskite quantum dots are also fabricated by solution-processable techniques. There are several reports in which different researchers reported the growth of barred and capsulated perovskite quantum dots by solution-processable methods [94, 95]. These quantum dots were then utilized to fabricate highly efficient optoelectronic devices [96–98]. Guo and co-workers used one-step ultrasonication bath treatment approach to make CH_3_NH_3_PbBr_3_ PQDs [94]. The diagram of complete “ultrasonication bath synthesis” process is shown in Fig. 7e. The process starts with the mixing of PbBr_3_ and CH_3_NH_3_ by ultrasonication followed by the formation of MAPbBr_3_ and absorption of ligands onto MAPbBr_3_ to form stable perovskite quantum. Blue background in all images shows the presence of TL solution. The TEM image of synthesized QDs is shown in Fig. 7f.

#### Vapor Phase Synthesis

The preparation route has a significant impact on structural morphologies and performances of the devices based on LD perovskite materials. The feasible synthesis technique to make them available for industrial applications is always desirable. In this section, the solution phase, vapor phase deposition, and templated synthesis have been demonstrated in a controllable and versatile way for the uniform and better geometry LD perovskites nanostructures. Some important factors that significantly affect the film quality including deposition temperature, solvent concentration, substrate temperature, and annealing conditions are also reviewed.

**Fig. 8:**
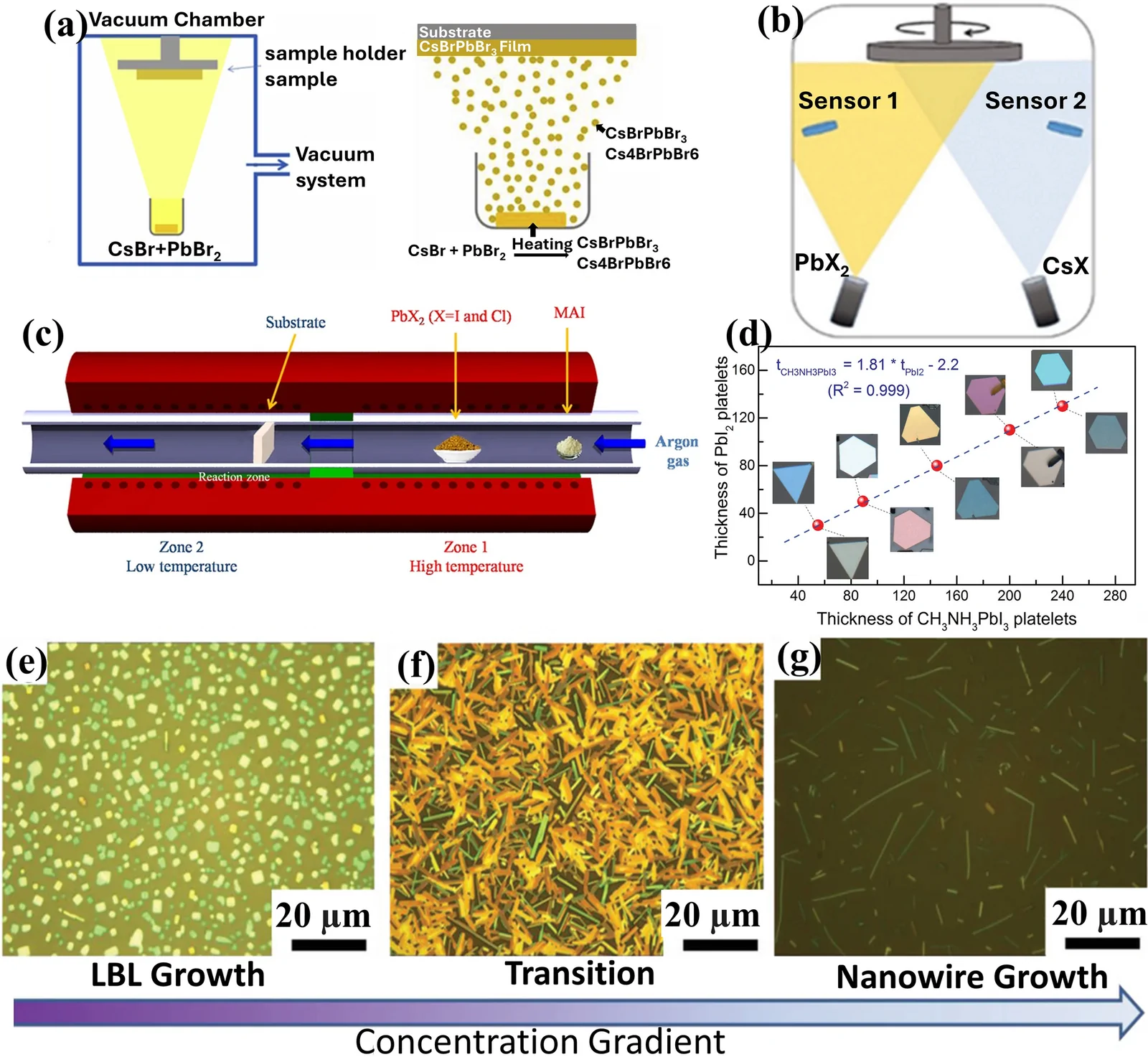
Schematic illustration of 2D perovskite deposition. **a** Single-source, **b** double-source deposition vacuum deposition method [101]. **c** Tube furnace synthesis of MAPBX_3_ [104]. **d** PbI_2_ platelet thickness and color variation before (above data line) and after (below data line) conversion to CH_3_NH_3_PbI_3_. Growth regimes as a function of supersaturation [79]. **e–g** Shape progression from nanoplatelets to nanowires as seen using an optical microscope. **e** Layer-by-layer (LBL) development at high precursor concentrations. **f** Intermediate transition state. **g** At decreasing precursor concentrations, the transformation to nanowire development occurs [105]

Comparing the crystallinity and defect density among mechanical exfoliation, solution-based, and vapor-phase techniques, the vapor phase synthesis is considered to be the better choice for higher quality LD nanostructures [99]. This is also advantageous for charging transport and several other properties in optoelectronic applications. Waleed et al. used the CVD technique to grow CsPbI_3_ NWs in a tube furnace having two heating environments [100]. Li et al. [101] have reported a single-source vacuum thermal evaporation (VTE) technique for the synthesis of CsPbBr_3_ films as shown in Fig. 8a.

Vapor phase synthesis has been widely used for perovskite nanostructures, by treating pre-grown lead halide nanostructures with gaseous MAI. They used CsBr and PbBr_2_ precursors to make pinhole-free and highly compact CsPbBr_3_ nanostructures. They also employed these perovskite films in photovoltaics, observing the influence of thickness and precursor material molar ratio on stoichiometry and photovoltaic performance. The device performance of CsPbBr_3_ PSCs enhances from molar ratio at 0.6:1 to 0.9:1 and then reduces constantly to 3.57% by increasing the molar ratio to 1.1:1. Wang et al. [102] produced 2D MAPbI_3_ perovskite NPs by one-step CVD method on mica substrates. They used dual precursors (PbCl_2_ and MACl_2_) for the direct growth of 2D MAPbI_3_ perovskite NPs. The schematic for dual precursor deposition is shown in Fig. 8b [103]. Xiong et al. utilized a two-step CVD approach to manufacture high-quality 2D MAPbI_3_ platelets. They grew PbI_2_ crystals on muscovite mica via VDW epitaxial growth and later transformed it to perovskite into a CVD furnace by interacting with MAI molecules. The thickness fluctuation of PbI_2_ platelets earlier (pictures above data line) and after conversion to CH_3_NH_3_PbI_3_ is shown in Fig. 8d (images below data line). The color of PbI_2_ platelets altered in proportion to the thickness change (assessed by AFM). The thickness of the CH_3_NH_3_PbI_3_ platelets was around 1.8 times that of PbI_2_ platelets, which is roughly in line with the two compounds c lattice constant ratio [79]. Commonly used two temperature zone furnaces consisting of the furnace, gas source, exhaust system, and quartz tube reactor are mentioned in Fig. 8c [104].

The concentration of deposited solution also plays a dominant role in the 2D crystal growth. Ghoshal, et al. used a CVD technique to examine the effect of concentration on the growth of 2D (C_4_H_9_NH_3_)_2_PbI_4_ perovskite. During the growing process, the depositing substrate was placed on the quartz tubes and kept at a pressure of 600 mtorr. The diagram depicts the growing process (Fig. 8e-g) demonstrates the concentration-dependent growth of samples of different morphologies. Higher concentration can be used to attain layer-by-layer (LBL) growth as shown on the substrates which were closest to precursors (nanoplatelets growth) as mentioned in Fig. 8e, while low concentration or farthest substrates from precursors grow as nanowires (Fig. 8g). The intermediate positions between LBL nanoplates and nanowires are shown in Fig. 8f [105]. Shaojuan et al. [106] used CVD fabrication in order to investigate the impact of different substrates on the morphology of fabricated 2D perovskites. They reported the controlled synthesis of 2D perovskite with higher quality on several substrates, including flexible polydimethylsiloxane (PDMS), mica, SiO_2_/Si, Si, and glass substrates.

#### Templated Synthesis Method

The liquid synthesis methods are widely used to fabricate perovskite materials with different thicknesses and phases [81, 107]. It is not easy to make a uniform assembly of perovskite nanowires by the solution method. Normally, mixtures of rods, wires, plates, and cubic structures are obtained with different size variations. It is also not possible to align the randomly distributed nucleation center and make equal sizes nanocrystals, nanowires or nanorods by solution-processable method. The alignment and orientation control of 1D perovskites are very difficult to achieve by simple solution-processable synthesis methods [108]. Therefore, to control alignments, pinholes, coverage area, aspect ratio, and orientation of the nanowires, the researchers are using the template method. They found the template-based method very effective to achieve an array of aligned micro- and nanofibers, nanowires, and nanotubes with adjustable diameter and length.

In this technique, a template (either nanoporous membrane) with uniform size pores of the same diameter is used to confine the precursor material into the required nanofibers/wires shape and structure and guide the growth direction [109]. Several porous materials can be used as templates to fabricate nanofibers and nanotubes. Uniform sizes and diameters of pores allow achieving control over dimensions of the nanofibers. The difficulties of the template-based synthesis method include selection of appropriate templates, filling the pores/template with perovskites, and then removing the template after the growth of the required material. All these challenges are worth exploring to enhance nanowire quality.

**Fig. 9:**
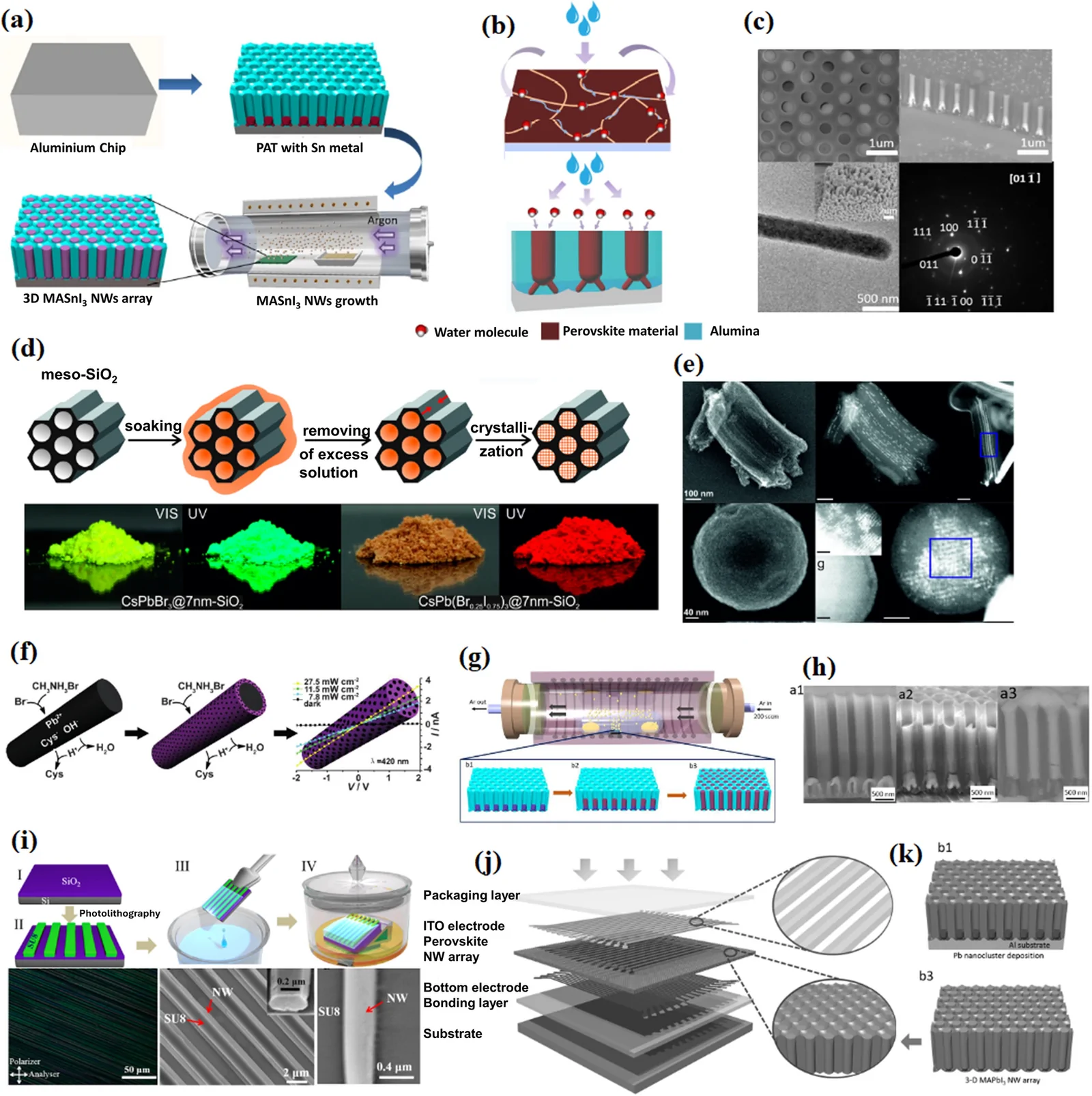
**a** Schematic diagram of template-based tin perovskite NWs obtained from MAI. **b** Decay of tin perovskite thin film (top image) and NWs sidewall protection of PAT from moisture (bottom). **c** Surface, top, and cross-sectional SEM image of tin perovskite nanowires and TEM of single NW [110]. **d** Multistep templated growth of CsPbX_3_ NCs in mesoporous silica pores. Bottom optical images show the mesoporous silica with CsPb (Br0.25I0.75)3 NCs (right) and CsPbBr_3_ (left) under UV and daylight. **e** SE-STEM and HAADF-STEM images of the partially filled samples with CsPbI_3_ NCs. Followed by EDXS of the area highlighted in the next Figure (bottom last Fig. 9e) [111]. **f** Synthesis of porous CH_3_NH_3_PbBr_3_ nanowires by self-template-directed chemical transformation [112]. **g** Schematic of CVD growth process of FAPbI_3_ NWs. Bottom images (b1-b3) show the template-assisted growth of vertically aligned NW array. **h** Cross-sectional SEM of NWs, grown at multiple steps [113]. **i** Schematic of the growth process for the fabrication of CH_3_NH_3_PbI_3_ NW array. Bottom images showing a cross-polarized optical image of the CH_3_NH_3_PbI_3_NW array and SEM image of NW array grown on the photoresist strips [114]. **j** Structural schematic of MAPbI_3_ NWs based image sensor with a magnified image showing 3D NW array inside the nanoengineering template. **k** Schematic showing the growth of high-density and vertically aligned MAPbI_3_ NW array: Pb in PAM before (b1) and after (b3) MAPbI_3_ NW growth [115]

Fan and co-workers reported nanoengineering templates to fabricate 3D perovskite (CH_3_NH_3_SnI_3_) nanowire arrays and used them for the first time in photodetectors with higher stability [110]. They also replaced the toxic Pb with Sn to make it environment-friendly and nontoxic. They employed a multistep manufacturing process, beginning with polishing and anodizing an Al foil to create a PAT having nanopore pitch and size, then electrochemically depositing Sn at the bottom of nanopores, as illustrated in Fig. 9a. Furthermore, the sample is placed next to CH_3_NH_3_I_3_ powder in a tube furnace at 170 °C to make CH_3_NH_3_SnI_3_ NWs. Complete fabrication steps are shown in four parts of Fig. 9a. CH_3_NH_3_I_3_ vapors are carried to the PAT by Ar carrier gas, where they react with Sn nanoclusters within the PAT's nanopores. They also reported the decomposition process in the CH_3_NH_3_SnI_3_ thin film and NWs. Decomposition in thin films is fast due to the presence of pinholes as schematically shown in Fig. 9b. This is unavoidable, although it can be mitigated by reducing grain boundary area and increasing grain size for improving stability. Regarding NWs, the PAT can effectively protect the MAI NWs due to its chemically and mechanically robust, electrically insulating, and optically transparent nature. As a result of the PAT protection, the NWs will disintegrate vertically, taking longer to break down the full NW array. A schematic of the interaction of water molecules with thin film and NWs is shown in Fig. 9b. Surface morphology and shapes of NWs are mentioned in Fig. 9c where SEM images show the apex of the grown MASnI_3_ NWs in a well-organized PAT. Perfectly hexagonal structures can be seen from SEM images; Fig. 9c displays a cross-sectional SEM representation of NWs and the bottom images show different alignments and cubic crystal structures of as-grown NWs.

Kovalenko and co-workers synthesized the colloidal Pb halide perovskite nanocrystals with a bright emittance of pure colors that cover the whole visual spectrum [111]. They reported the superiority of these nanocrystals over conventional quantum dots due to their defect-tolerant photo physics. They used mesoporous silica pores as templates and filled these templates with perovskite precursors. The obtained perovskite NCs were also able to achieve very brilliant PL with quantum efficiencies reaching 50% after drying. The schematic of synthesis assembly is shown in Fig. 9d. Scanning transmission electron microscopy (STEM) was used for the structural analysis of meso-SiO_2_ samples with different pore sizes, e.g., impregnated with either MAPbI3 or CsPbI_3_ at 7, 4, and 2.5 nm as shown in small figures of Fig. 8e. The first figures show the mesoporous structure with 1D channel of about 2.2 nm separation. The filling of pores with CsPbI_3_ can be seen by area selective energy-dispersive X-ray spectroscopy (EDXS) and dark field mode (HAADF-STEM) Fig. 9e (bottom). The final shape and sizes of filled material depend upon the pore sizes and shapes and can be homogeneously distributed throughout the entire particle within its porous structure. Continuing the preparation of porous perovskite nanowires, Zhang and colleagues created porous perovskite (CH_3_NH_3_PbBr_3_) nanowires using a self-template-directed synthesis method at ambient temperature and employed them in high-performance photodetectors [112]. They employed a room temperature solution method, using lead-containing precursor nanowires as the Pb source and sacrificial template. The conversion of Pb-containing nanowires into porous perovskite nanowires proceeds via a self-template-directed reaction with HBr and CH_3_NH_3_Br. The final 1D structure is formed by the disintegration of organic components. Initially, layers based on perovskites were formed on top of Pb-containing nanowires. Pb has a significant chemical reactivity with HBr and CH_3_NH_3_Br leads to the formation of CH_3_NH_3_PbBr_3_. The brief mechanism is shown in Fig. 9f.

Gu et al. used a vapor phase growth technique at a very limited level in nanoporous templates to stabilize the black phase of FaPbI_3_ nanowires [113]. FaPbI_3_ with higher decomposition temperature and lower disintegration process has been widely used in high-efficiency and long-term stable optoelectronic devices. Especially the alpha phase of FaPbI_3_ is very prominent, and numerous unique optoelectronic characteristics are exclusive to the alpha phase. Unfortunately, the desired alpha phase converts into the undesirable delta phase, which has significantly worse optoelectronic properties. Fan and co-workers overcome the stability issue by applying the nanoengineering templates approach to the cubic black phase of FAPbI_3_ and achieved stability over 7 months without any further treatment at ambient conditions. They also reported the 8 days stability in an extremely humid environment with 97% humidity. Figure 9g depicts the FAPbI_3_ NWs growth process in a schematic diagram. Cross-sectional SEM images showing the development of NWs at various phases are shown in Fig. 9h(a1-a3). Figure 9h(a1) shows the initial Pb clusters before the growth of NWs, Fig. 9h(a2) shows half-grown NWs, and Fig. 9h(a3) shows the over-grown NWs.

Deng et al. fabricated high quality with smooth surface single-crystal perovskite NWs employing the fluid-guided antisolvent vapor-assisted crystallization technique on a large scale [114]. The fabricated NWs consist of MAPb(I_1−x_Br_x_)_3_ arrays with x = 0, 0.1, 0.2, 0.3, and 0.4. Figure 9i shows the schematic of the step-by-step growth process of single-crystalline perovskite (CH_3_NH_3_PbI_3_) NW arrays. Initially, they fabricated photoresist stripes on the SiO_2_/Si substrate by photolithography. These strips act as a template to grow these NW arrays. The further process consists of dipping the photoresist template into MAPbI_3_/DFM solution, dragging it out with the hanging solution, placing with CH_2_Cl_2_ solvent on a tilted glass (∼5°), and sealing the fabrication assembly bottle at room temperature. The perfectly constant color shows the growth of continuous and uniform NW arrays at a large scale (Fig. 9i). SEM image shows perfectly aligned NWs with inset showing well-shaped and uniform angular facets (inset in Fig. 9i 2nd SEM image). The last SEM image at higher magnification shows the smooth and crack-free surfaces of CH_3_NH_3_PbI_3_ NW arrays (Fig. 9i). Gu et al. used a novel vapor solid–solid reaction (abbreviated as VSSR) method for the growth of 3D MAPbI_3_ NW arrays in porous alumina membrane (PAM); these PAM are applied to nanoengineering templates [115]. The growing process is initiated by Pb metal nanoclusters at the base of vertical nanochannels. Nanochannels are crucial for controlling the geometry of 3D NWs and grow ultrahigh NW density. Gu et al. used the 3D NWs to fabricate high-efficiency image sensors. Figure 9j depicts a magnified diagram of 3D NW array inside the nanoengineering template, as well as the six-layer structural device of an image sensor based on 3D-NW arrays. The 3D MAPbI_3_ NW array is between bottom gold (Au) metal electrode and the upper ITO electrode. Figure 9k shows the schematic of perovskite NW development at high density in a PAM. Figure 9k(b1) leads in PAM before and after MAPbI_3_ NW growth (Fig. 9k(b3)).

#### Growth of Single-Crystal LD Perovskites

**Fig. 10:**
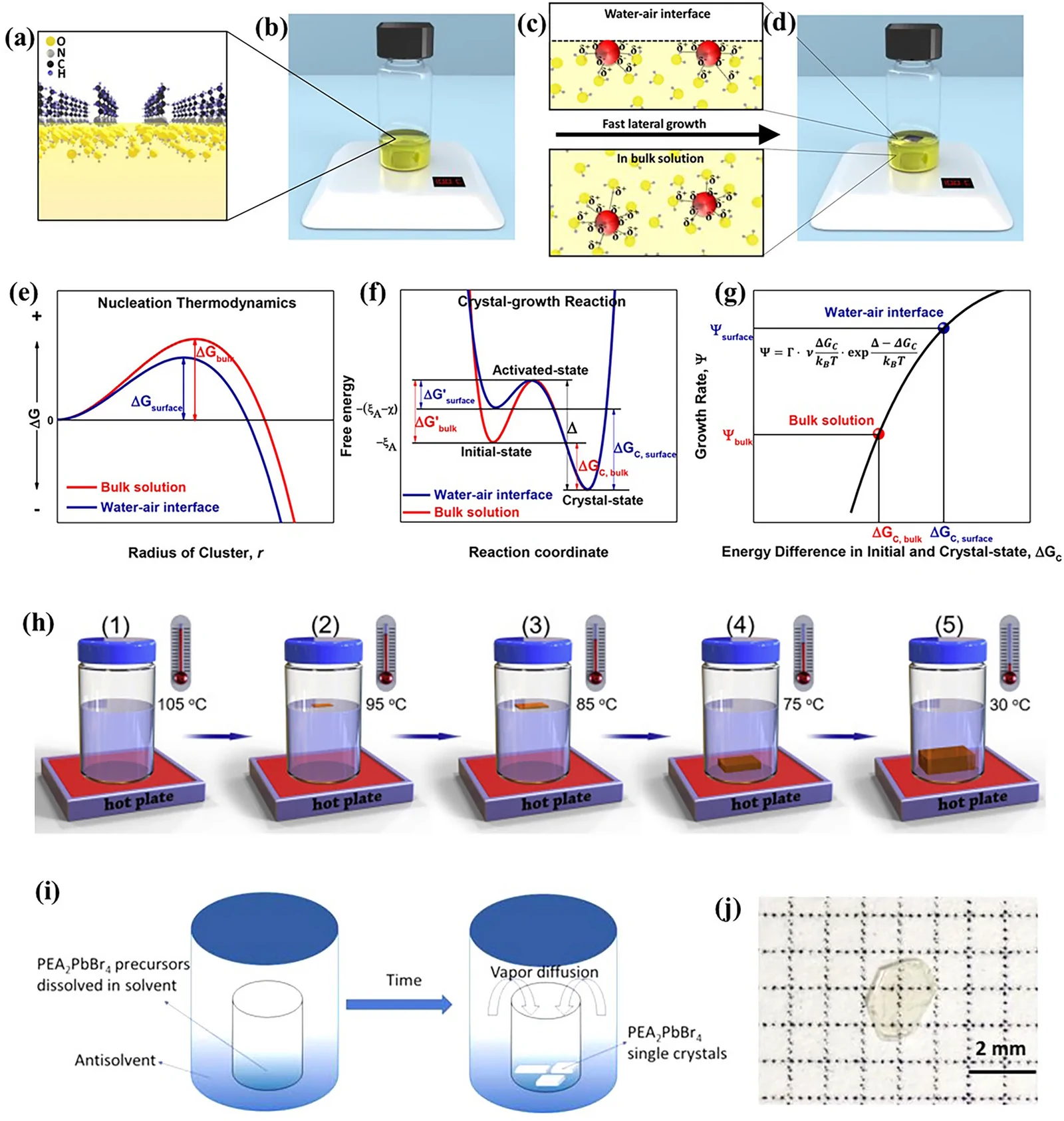
Illustration and experimental setup for the synthesis of PEA₂PbBr₄ single crystals at the water–air interface. **a** Schematic of molecular assembly at the interface during nucleation. **b** Experimental arrangement with a precursor solution heated on a hot plate. **c, d** Comparison of crystal growth dynamics at the interface and within the bulk solution, highlighting the rapid lateral growth at the interface. **e** Nucleation thermodynamics showing the difference in energy barriers for bulk versus interfacial nucleation. **f** Energy landscape of the crystal growth process, indicating transitions between initial, activated, and crystalline states. **g** Growth rate dependence on energy variations in different environments [116]. **h** Controlled cooling approach for crystal formation at different temperatures [117]. **i** Vapor diffusion technique for crystallization using an antisolvent. **j** Optical image of a PEA₂PbBr₄ single crystal [118]

Perovskite single crystals (PSCs) with higher optical absorption, smaller trap-state density, higher carrier lifetime, and better carrier mobility make them prominent than 3D PSCs. 2D layered PSCs are considered an alternative to typical 3D PSCs with better stability and higher performance toward optoelectronic devices. However, the implementation of 2D PSCs is limited by the lack of preparation methods for the high-quality and uniform growth of PSCs. 2D PSC-based devices exhibit better performance than 3D PSCs for certain optoelectronic applications, particularly those fabricated on the (001) plane. Wang et al. employed density functional theory (DFT) and nucleation theory to develop a supersaturated solution. They then applied the concept of thermodynamic proportionality to temperature to synthesize quasi-2D perovskite single-crystal membranes (C₄H₉NH₃)ₙ(CH₃NH₃)ₙ₋₁PbₙI₃ₙ₊₁ (where n = 1, 2, 3, 4, and ∞) [116]. The schematic illustration and growth mechanism are shown in Fig. 10. Figure 10a depicts the orientation and alignment of the butylammonium cation, while Fig. 10b presents the experimental setup for the growth process. Figure 10c, d illustrates the interaction of molecules, where the tensile force on the molecules at the water–air interface is higher. Figure 10e-g provides a graphical representation of the cluster radius dependence on nucleation, variations in the large free energy, and a comparison of the growth rates of precursor molecules in bulk versus the air–water interface, respectively. Similarly, Liu and co-workers used the advantage to grow the 36-mm sized 2D (PEA)_2_PbI_4_ PSCs by using the concept of classic nucleation theory [117].

Figure 10h shows the step-by-step process schematic of (PEA)_2_PbI_4_ PSCs growth assisted by the surface tension mechanism. Tian et al. fabricated exfoliated (PEA)_2_PbBr_4_ 2D PSCs and then fabricated resistive switching memory on them [118]. Figure 10i shows the schematic of the growth process of (PEA)_2_PbBr_4_ of PSCs, and Fig. 10j shows the photograph of a transparent 2 mm single crystal. Recent advancements in crystallization techniques have enabled the growth of large, high-quality 2D perovskite single crystals, leading to devices with remarkable performance metrics, including high responsivity and fast response times. These developments pave the way for the commercialization of PSC-based devices in photodetection and imaging technologies.

#### Theoretical Simulations and AI-Assisted Design for LD Perovskites

The fundamental properties of low-dimensional perovskites can now be investigated effectively using atomistic simulations and first-principles computations. The unique characteristics of LD perovskites are best understood through theoretical investigations and structural simulations. MD simulations and DFT are two computational methods that are frequently used to forecast their electronic band structures, stability, and defect tolerance. Hardik et al. investigated the effects of A site cation changes (formamidinium (FA) and methylammonium (MA)) on their optical, electrical, and structural characteristics. With potential power conversion efficiencies of 30% and strong optical absorption in the visible and ultraviolet spectrums, both technologies hold potential for solar cells. The results offer important facts for developing and refining 2D perovskites for cutting-edge optoelectronic applications [119]. Dong et al. use DFT calculations to examine the connection between the band gap (E_g_) and dielectric constant (ε) in LD perovskites (CsPbX₃, X = Cl, Br, I). A theoretical framework for comprehending dielectric behavior is provided by the establishment of a linear connection between ε and 1/E_g2_. This study enhances LD perovskites and helps build effective materials for solar cells, LEDs, and other optoelectronic devices [120].

Ozorio et al. examine how the stability and optoelectronic characteristics of two prototype structures C_20_H_20_N_4_B_2_X_10_C_20_H_20_N_4_B_2_X_10_ (edge-sharing octahedra) and C_30_H_25_N_6_BX_6_⋅ 2H_2_OC_30_H_25_N_6_BX_6_⋅2H_2_O (isolated octahedra) are affected by halide anions (Cl, Br, I) and trivalent cations (Bi, Sb). They observe that halide substitution affects structural stability and bandgap more than cation substitution, with greater bandgaps and higher stability resulting from smaller halides (Cl) [121]. Hang et al. theoretically investigate the structural and optoelectronic properties of the lead-free perovskites RbGeBr₂Cl and RbGeI₂Cl, using DFT calculations with the Perdew–Burke–Ernzerhof (PBE) functional and the GW approximation for accurate bandgap predictions. They demonstrate that both materials have direct bandgaps, tunable optoelectronic properties, and significant UV absorption [122]. The structural, electrical, optical, and thermoelectric characteristics of LD perovskites, namely Cs₂B'GeCl₆ (where B' = Zn, Cd), are investigated utilizing sophisticated theoretical techniques based on DFT. The generalized gradient approximation (GGA) and hybrid functional techniques are used in computations to assess important features such density of states (DOS), band structures, optical absorption spectra, thermoelectric performance, and lattice parameters [123].

Regarding MD simulations, Beck et al. perform MD simulations to study the binding and structural properties of the halide perovskite MAPbI₃. van der Waals (vdW) corrections, particularly the Tkatchenko–Scheffler (TS) and many-body dispersion (MBD) techniques, are shown to be essential for accurate predictions of lattice constants and bulk modulus. Methylammonium (MA) orientation has an impact that shows that antiparallel MA configurations result in octahedral tilting and lower system energy, while parallel alignment preserves cubic symmetry but poorer stability. Highlighting the importance of dynamic effects, the results provide a computational framework for perovskite material engineering [124]. Magnus et al. use MD simulations to study the structural dynamics of two-dimensional Ruddlesden–Popper perovskites (2D RPPs) with the general formula BA₂MAₙ₋₁PbₙI₃ₙ₊₁, where n is the number of inorganic lead iodide layers, BA is butylammonium, and MA is methylammonium, providing significant insights on the effects of dimensionality on organic cation dynamics and inorganic framework. In 2D systems, MA rotation is more limited by the asymmetric environments produced by BA spacer layers, whereas BA mobility is impacted by its interaction with the inorganic framework and screening by MA cations. Findings demonstrate the intricate relationship between the dynamics of organic components and the inorganic lattice structure in low-dimensional perovskites [125].

Rafael et al. promote the use of lead-free perovskites (XSnI₃, X = Cs, K, Rb) in solar cells for green policy efforts by using MD simulations to examine the mechanical characteristics of these materials under environmental stressors. Anisotropic mechanical behavior by simulations, with RbSnI₃, shows the lowest stiffness (0.131 GPa) and CsSnI₃ the greatest (Young’s modulus: 1.361 GPa). Stress tolerance is strongly influenced by temperature and pressure; performance peaks at 270 K and decreases at higher pressures (200 MPa) [126].

Considering the AI and machine learning for the development of LD perovskites, Ruiyang et al. employed ML-driven approach to predict the structural dimensionality of LD perovskites based on ammonium cation properties and found four important chemical descriptors: steric effect index (STEI), eccentricity (Ec), largest ring size (LRS), and hydrogen-bond donor count (HDC) after compiling 86 reported amines and categorizing them as either 2D-forming or non-2D-forming. They developed an 82% accurate prediction model using logistic regression [127]. Wissam et al. employed an AI-driven method that speeds up materials screening by orders of magnitude in comparison with DFT, which highlights the significance of structural features in property prediction while facilitating the effective exploration of large chemical spaces. They further illustrate how hierarchical ML models can fill computational and experimental gaps in perovskite design and can improve its forecasting potential for optoelectronic applications [128]. Also, Songyang et al. uses a K-nearest neighbor model to predict the dimensionality (2D, 1D, or 0D) of LD perovskites based on organic spacer characteristics with 92.3% accuracy. They draw attention to how AI-assisted design has sped up the discovery of LD perovskites, and the incorporation of theoretical simulations in the future may improve prediction accuracy [129]. Sum et al. offer a geometric data analysis (GDA)-based machine learning method for designing 2D perovskites. They suggest using an innovative method termed density fingerprinting (DF) to depict the periodic and geometrical structural details of perovskite unit cells. To forecast material characteristics, especially bandgaps, these DFs are coupled with a gradient boosting tree (GBT) model [130].

## Applications for LD Perovskites, Advances, Challenges, and Perspectives

### Fundamental Advantages over Conventional Semiconductors

LD perovskites challenge traditional semiconductors by integrating high-performance properties with remarkable solution processability, unlike silicon, which necessitates epitaxial growth at higher temperatures [131]. Low-temperature (< 150 °C) solution processing allows LD perovskites to reach exceptional electrical quality [132]. New developments in 2D Ruddlesden–Popper perovskites have shown that out-of-plane charge carrier mobilities can surpass 100 cm^2^ V^−1^ s^−1^ [133], rivaling single-crystal silicon and allowing for flexible electronics that are not possible with inorganic materials that are stiff. These materials exhibit particularly remarkable quantum confinement effects; in 2D perovskites such as (PEA)₂PbI₄, exciton binding energies can approach 200–400 meV [135, [135]. These values are significantly higher than those of transition metal dichalcogenides (~ 20–100 meV) and GaAs (~ 4 meV), which are typical semiconductors [136], permitting strong excitonic occurrences at normal temperature. LEDs have demonstrated exceptional device performance due to this basic advantage, retaining 28.5% external quantum efficiency at realistic brightness levels of 10,000 cd m^−2^ [137].

The special qualities of LD perovskites address long-standing issues in a variety of optoelectronic applications. In terms of display technology, they provide higher color purity with NTSC 150% color gamut while also overcoming the efficiency roll-off that organic LEDs (OLEDs) lacks [137]. In photovoltaics, LD perovskite/silicon tandems use dimensionality-controlled bandgap tuning (1.2–2.3 eV) [138] to possibly achieve efficiency levels beyond 40%, which are beyond the reach of single-junction silicon cells. New developments in on-chip photonics have shown that 2D perovskite optical modulators with a bandwidth of 100 GHz can surpass traditional silicon photonic devices while still being compatible with CMOS [139–141]. Exact comparisons between organic and inorganic ETLs in perovskite devices have been made possible by numerical modeling studies in 2023–2024; under simulated module settings, inorganic variations exhibit greater thermal and photostability. When included into CH₃NH₃SnI₃-based architectures, Cu-based inorganic HTLs showed over 18% efficiency and strong performance metrics, making them perfect for the deployment of scalable, affordable solar modules. Full-scale PV simulations verify that inorganic transport layers provide long-term operational reliability under a variety of environmental conditions in addition to lowering recombination losses [142–145].

Research in 2023–2024 has shown previously unheard-of possibilities using LD perovskites. 2D perovskite memristors with ultralow 0.1 fJ energy per switching event [9] and 10⁶ unique conductance states are advantageous for neuromorphic computing applications, offering a 100 × improvement over traditional oxide-based devices. The cryogenic restrictions of conventional quantum dots have been removed by room temperature single-photon emitters that exhibit 90% purity [146], revolutionizing quantum technologies. 0D Cs₃Cu₂I₅ X-ray detectors outperform commercial amorphous selenium systems in medical imaging, achieving an impressive sensitivity of 0.07 μGyₐ_ir_ s^−1^ at just 1 V bias [147].

The qualities which make LD perovskites very remarkable include the reversible bandgap tuning via mechanical strain that surpasses 300 meV [148]. For innovative optical applications, natural light polarization ratios are greater than 10:1 [149]. Also, inorganic-like charge transport and molecular tunability are made possible by hybrid organic–inorganic activity [150]. These developments put LD perovskites in a position to facilitate whole new technological paradigms rather of only serving as substitutes for current semiconductors. Future research directions should focus on establishing global stability standards for implementation in the industry [151] and employing high-throughput screening to create extensive materials databases [152], as well as integrating modern manufacturing processes to expand production [153].

#### Limitations of LD Perovskites

LD perovskites are a promising family of semiconductor materials, but to reach their full potential, a number of important issues need to be resolved. Anisotropic charge transfer, ambient instability, and production scalability concerns are the most urgent restrictions; they have all received a lot of study attention lately.

Significant differences in charge transport characteristics are caused by the intrinsic anisotropy of 2D perovskites. Significant quantum confinement effects can cause out-of-plane mobilities to surpass 100 cm^2^ V^−1^ s^−1^ [154], whereas insulating organic spacers restrict in-plane transport, which usually exhibits mobilities under 20 cm^2^ V^−1^ s^−1^ [155].

Significant gains have been made recently in molecular engineering, with conjugated spacers such as thieno[3,2-b]thiophene-2-ethylammonium showing increased environmental stability and enhanced charge transfer (up to 65 cm^2^ V^−1^ s^−1^ in-plane mobility) [156]. To further optimize charge transport channels, preferred orientation of quantum wells has been made possible by innovative crystallization procedures that involve solvent vapor annealing [157].

One of the key challenges is maintaining environmental stability while operations are underway. Degradation processes including organic cation breakdown and halide migration continue to occur even though LD perovskites exhibit better stability than their 3D counterparts [158]. Recent advancements in encapsulation technology have shown notable gains in stability, especially in the deposition of hybrid organic–inorganic barriers using atomic/molecular layers [159]. After 2,000 h of constant illumination at 85 °C, for example, devices with AlO₃/parylene nanolaminate encapsulation retained > 95% original efficiency [160]. Fluorinated spacer cations have also been used to increase intrinsic material stability, improving both thermal stability up to 150 °C and moisture resistance [161]. Another major obstacle to commercialization is manufacturing difficulties. Inconsistent phase distribution and film shape are frequently caused by the intricate crystallization processes of LD perovskites. Many of these problems have been resolved by recent advancements in deposition methods and ink engineering [162].

It is now possible to create homogenous films over 15 × 15 cm^2^ regions with a thickness variation of less than 5% thanks to slot-die coating and improved solvent solutions [163]. Furthermore, phase purity and orientation control in large-area devices have been enhanced using vapor-assisted crystallization techniques [164]. Rapid examination of hundreds of processing parameters is now possible because of the advent of high-throughput screening technologies, which have significantly sped process optimization. Emerging solutions provide a number of interesting lines of inquiry. To anticipate crystallization behavior and design optimal spacer molecules, machine learning techniques are used [165]. In situ grazing-incidence X-ray scattering is one of the advanced characterization methods that offer previously unheard-of insights into the kinetics of film development [166]. Additionally, the advancement of universal stability methods is making it possible to compare material performance across many research groups with greater accuracy [167].

### Memory Devices and Synapse

Researchers are working to reduce the power consumption in memristors to link the challenges of massive data. The key to achieving minimal power utilization is by reducing operational current. Due to the presence of larger densities of defects, such as interstitials and vacancies in perovskite-based memristors, significant leakage current has been found, which will thus lead to extremely low power consumption for devices. Recently, LD perovskites-based memory devices with high ON/OFF current ratios [118], long-term retention [168], low operating voltages [169], and multilevel resistive switching [170] are emerging as future smart memory devices [171–173].

**Fig. 11:**
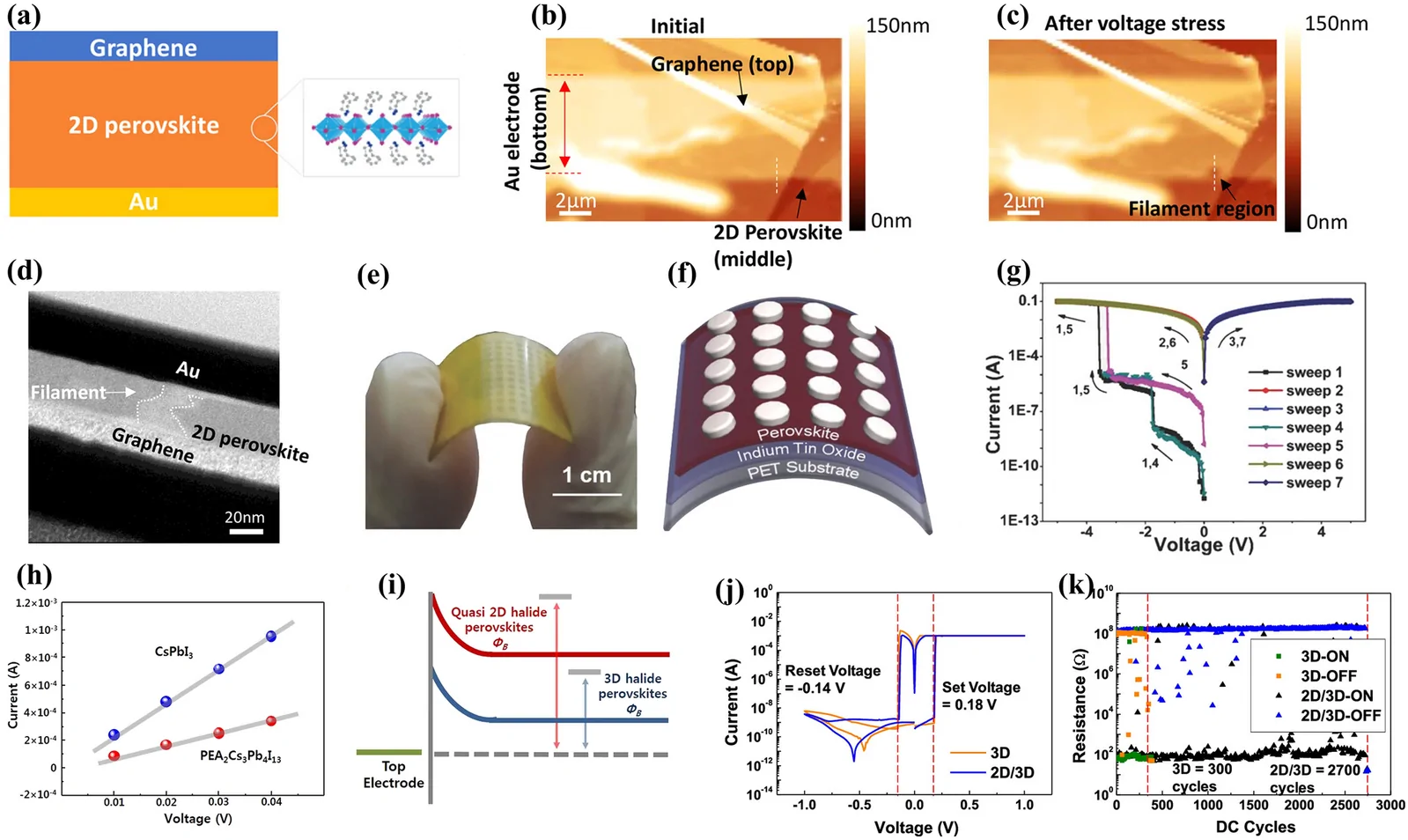
**a** Structural schematic of graphene/2D perovskite/Au vertical device. **b** AFM of a fresh and **c** after the application of pulsed voltage. **d** TEM image representing filament shape in white dotted line at ∼30 nm (diameter) close to graphene and approx. 15 nm close to Au side [118]. **E** Original and **f** schematic diagram of ITO-PET/perovskite film/Al (flexible) device. **g** Current − voltage (I–V) curve of ITO-PET/ MA_2_PbI_2_(SCN)_2_/Al -based memory device [176]. **h** Comparison of linear replotted I–V curves for the HRS of (PEA)_2_Cs_3_Pb_4_I_13_ and CsPbI_3_ based devices. **i** Schematic diagram showing Schottky barrier heights for (PEA)_2_Cs_3_Pb_4_I_13_ and CsPbI_3_ [177]. **j** I − V properties of 2D/3D heterostructure perovskite-based memristor. **k** Endurance performance comparison between 3D (350 cycles) and 2D/3D heterostructure device (2,700 cycles) with fixed pulse period of 640 μs [178]

Ren et al. fabricated a 2D organic–inorganic hybrid memristor with low power consumption [118]. The device structure was Au/(PEA)_2_PbBr_4_/graphene where PEA represents phenethylammonium. Figure 11a shows the schematic diagram of graphene/2D perovskite/Au vertical device with structure. Filament formation is observed from Figs. 11b (initial state) and 9c(after voltage stress) by using atomic force microscopy (AFM) images. Achieving the charge transport suppression in 2D (PEA)_2_PbBr_4_ film, a very small program current (10 pA) is enough to operate the memristor, which is much lower than previously reported memristors. Figure 11d shows the TEM image of filament formation in the device. Seo et al. fabricated 2D hybrid perovskite-based memristor in 2017. They compared the properties of these 2D perovskites-based memristors with 3D variants [174]. The device has Si/SiO_2_/Ti/Pt/BA_2_MA_n−1_PbnI_3n+1_/Ag structure where BA is butylammonium, BA_2_PbI_4_ (n = 1) is 2D perovskite, when n = 2,3 were quasi-2D, and MAPbI_3_ (n = ∞) represents 3D perovskite. Quasi-2D perovskites memristors show much reduction in the set voltages and an increase in the ON/OFF current ratio from 10^2^ to 10^7^. This was explained based on thermal activation energy and higher Schottky barrier heights of 2D perovskite. Tian et al. [118] used 2D (PEA)_2_PbBr_4_ perovskite single crystals to fabricate graphene/ (PEA)_2_PbBr_4_/Au memory devices with as low as ≈0.1 pA I_OFF_ and 10^2^ ON/OFF current, showing the minimum energy consumption of the device. They also reported multilevel storage for several compliance currents. Lee and co-workers reported the effect of sequentially vapor-deposited BA_2_PbBr_4_ grain size impact on the performance of ITO/BA_2_PbBr_4_/Au memory cells [175]. Multiple grain sizes (180 nm, 1 µm, 30 µm) were achieved at several transformation temperatures (100, 150, and 200 °C, respectively). There is no effect of grain size on I_ON_ while I_OFF_ reduced from 10^–4^ to 10^–8^ A, resulting in an increase in ON/OFF current ratio from 5 to 2.4 × 10^3^. It has been reported that low-dimensional (0D, 1D, 2D) perovskites are more stable as compared to bulk 3D perovskites and 3D perovskites can be transformed into 2D by substituting the cations or halides parts of larger groups. 2D hybrid perovskite was synthesized by substituting pseudohalide such as thiocyanate (SCN −). Lu and co-workers prepared 2D MA_2_PbI_2_(SCN)_2_ and deposited by solution-processable method and achieved memristive response of the ITO/MA_2_PbI_2_(SCN)_2_/Al memristors [176]. The original and schematic of flexible device is displayed in Fig. 11e, f. Sweeping the voltage between ITO and Al electrodes, binary or ternary memory switching was achieved. Ternary switching is shown in Fig. 11g.

Recently, Jang and co-workers in 2020 made Si/SiO_2_/Ti/Pt/(PEA)_2_Cs_3_Pb_4_I_13_/Ag memristor based on quasi-2D (PEA)_2_Cs_3_Pb_4_I_13_ perovskites [177]. They achieved a higher ON/OFF current ratio of up to 10^9^, which was much higher than all-inorganic CsPbI_3_ 3D memristor. Higher activation energy, wider bandgap, and the Schottky barrier height of quasi-2D (PEA)_2_Cs_3_Pb_4_I_13_ perovskite make them prominent against 3D CsPbI_3_ perovskites. Higher ON/OFF current ratio, low I_OFF_ values, and improvements in stability against moisture by preventing PEA cation degradations under ambient conditions provide a long-time response of up to 2 weeks.

As we reported earlier, the stability of 2D perovskite-based memristors is much better than 3D based memristors. Recently, in 2020, Jang et al. reported the distinguished performance in terms of stability and high ON/OFF ratio of memory devices based on (PEA)_2_Cs_3_Pb_4_I_13_. They achieved ON/OFF ratio as high as 10^9^, which is almost three times greater than that of an ordinary CsPbI_3_ device. The higher value of the ON/OFF ratio was explained based on reduction in high-resistance state current formation. Under ambient circumstances, they reported the ON/OFF current ratio was high till two weeks, whereas CsPbI_3_-based devices degraded in 5 days. These achievements reveal that quasi-2D halide perovskites are more reliable as compared with 3D perovskite-based devices in resistive switching memory with higher performances and long-term stability. Comparison between I–V curves of 3D CsPbI_3_ and quasi-2D (PEA)_2_Cs_3_Pb_4_I_13_ at HRS is shown in Fig. 11h. The quasi-2D-based memristor shows a much lower current in the HRS. A schematic showing Schottky barrier heights for CsPbI_3_ and (PEA)_2_Cs_3_Pb_4_I_13_ is mentioned in Fig. 11i. Jung et al. fabricated 2D/3D heterostructure perovskite films by low-temperature solution process deposition and observed memristive performance with long-term endurance of about 2700 cycles, higher on/off ratio of 10^6^ with 640 μs operation speed [178]. Because of its higher thermal conductivity, they found that 2D perovskite may control the Ag conductive filament's rupture. Figure 11j shows the device's I–V curve under the DC sweeping voltage (0 V → + 1 V → 0 V → − 1 V → 0 V). The endurance performance of the device is indicated in Fig. 11k. The 3D perovskite device's endurance was reported to be only 350 cycles, which is significantly shorter than the 2D/3D perovskite device's endurance (∼2,700 cycles). In 2018, Kim et al. fabricated highly stable memory device based on CsPbCl_3_ PQDs surrounded in poly(methyl methacrylate) (PMMA) [179]. They reported 2 × 10^4^ ON/OFF current ratio and 1 × 10^4^ s stability of the device. Recently in 2021 Lee and co-workers reported all-inorganic perovskite quantum dots based light-emitting memories and transformed to light-emitting electrochemical cell [180]. Wang et al. in 2021 fabricated 1D perovskite-based resistive memory with ultra-low operating voltage about 0.2 V and bipolar I–V hysteresis [172].

### Memory Devices-based optically driven synapses

Biological synapses play a significant role in the signal’s transmission for memory, learning, and several other processes with very small power consumption. The biological synapses perform the computing and memory functions simultaneously. This makes the human brain capable of low power (100 fJ) consumers for each synaptic function. The central nervous system is made of ≈10 ^11^ neurons, linked by ≈10^15^ synapses to form a complete architecture for the human brain [181]. Rapidly increasing demand of fast data transfer systems as compared to conventional von Neumann architecture-based computing architecture, big data, and development of internet technologies, the significance of next-generation data computing components with minimum energy consumption by overcoming the limitations of separated main memory and central computing unit (CPU) is becoming more prominent [182]. Researchers are working to attain an alternative to traditional von Neumann architecture in which we need two different processing units for memory and information processing with the consumption of high power to perform neuromorphic computation [183]. Recently, synaptic devices are considered to be an alternative to classical von Neumann architecture as they can perform complex signal processing including decision-making, image classification, and involve the use of artificial neural networks for pattern recognition [184, 185]. Through the removal of the von Neumann architecture's bottleneck, neuromorphic computing simulates the function of biological synapses in information processing. In neuromorphic operating systems, mimicking synapse processing using these devices is crucial. Researchers have driven synaptic devices by electrical and optical signals to encourage the modeling of various synaptic processes. These devices can be classified into electrically, photoelectrically, and optically stimulated synaptic devices depending on the activation of electrical and optical signals, respectively [186].

Many studies are focusing on the device architectures and materials properties for the use in synaptic devices, but the van der Waals (vdW) heterostructures of 2D materials are among the best of researcher’s choice due to their unique mimicking capacity in synaptic plasticity for neuromorphic computing, which is based on the memristive hardware neural networks [187–189]. Different structural and compositional arrangements of perovskite halides make them versatile in electronic, physical, and chemical properties. All-inorganic, as well as organic–inorganic mixed perovskite halides, are extensively utilized for the fabrication of synaptic and memory devices [190, 191]. Under constant irradiation of light, the defects migration in the perovskite layer generate a higher hysteresis in IV response, resulting in better memory performances and artificial synapse devices applications for neuromorphic computing. Thus, defects present in the perovskite layer due to halide vacancies perform an important job in the memory functionalities and characteristics of the synaptic devices. In recent years, the 2D perovskite-based RRAM and the flash memory systems were used to construct the advanced artificial synaptic devices including two-terminal memristive synapses and three-terminal FET-based synapses to mimic the biological synapses. Simultaneously, the perovskites are more promising candidates for utilization in the artificial synapse with the capabilities of concurrent processing and learning due to their ionotropic effects as compared to other semiconductor materials. The movement of halide ions between the layers plays an important role in the conductance of 2D halide perovskite-based devices. This phenomenon is very similar to the synaptic conductance mechanism through intracellular Ca^2+^, Na^+^, and K^+^ ions in neurons. Considering the ionotropic effects and better phototunable properties in halide perovskites-based memory devices, the electric bias and photonic illumination are used as external stimuli to simulate several synaptic operations including short-term potentiation (STP) and depression (STD), long-term potentiation (LTP), and depression (LTD), paired-pulse depression (PPD) and facilitation (PPF), spike time-dependent plasticity (STDP), and spike rate-dependent plasticity (SRDP).

**Fig. 12:**
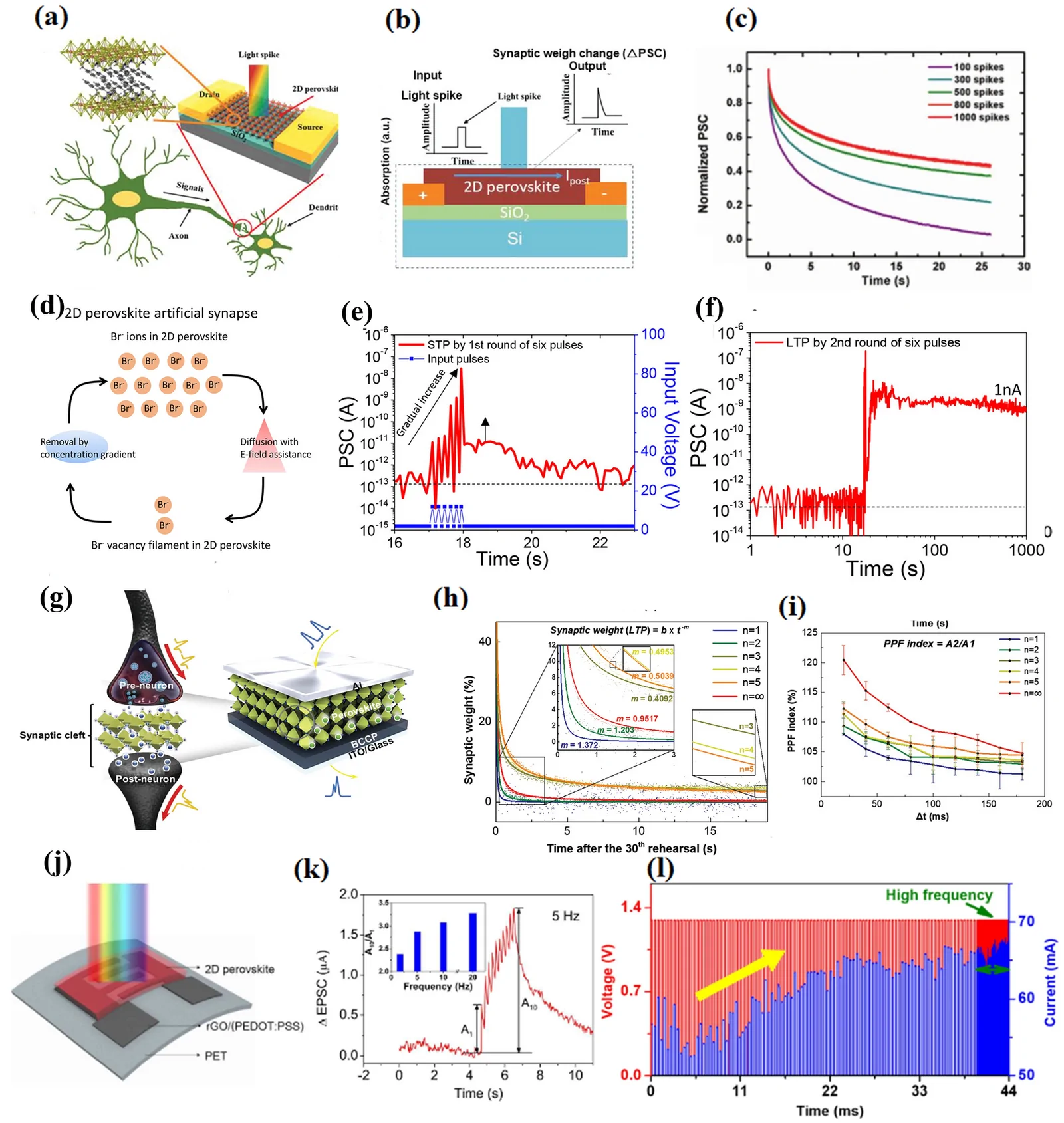
**a** Structural schematic of the device and biological synapses with axons to dendrites. Insects show crystal structure of 2D layered perovskite. **b** Side view schematic of device with input light spikes and the output PSC in the insets as an example. **c** Normalized PSC decay under 100 to 1000 light spikes [192]. **d** Schematic of Br ion migration in 2D perovskite-based artificial synapse. **e** STP at first round of six pulses. **f** LTP at the second round of six pulses with the increase in current level to 1 nA with long-term (1000 s) retention [118]. **g** Schematic diagram showing the artificial synapse and perovskite-based artificial synapses device geometry. **h** Decay curves for the current after the 30th rehearsal of perovskite synapses. **i** PPF index under several applied pulses with multiple time intervals 20 ≤ ∆t ≤ 180 ms [194]. **j** Structural schematic for a (PEA)_2_SnI_4_ flexible photoconductor. **k** Change in EPSC under 10 light pulses with time intervals of 200 ms [195]. **l** Neuromorphic behavior at gradual current change under continuous pulse voltage [196]

In 2019, Sun and co-workers made a photoelectrically stimulated synapse with Pb-free 2D perovskite-based ((PEA)_2_SnI_4_) [192]. They discovered photocurrent activation in a neuron-like process under light illuminations and possess synaptic capabilities such as STP and LTP. They successfully modulated the synaptic connectivity by exposure, irradiance, and wavelength of light spikes. The ternary structure of reported 2D perovskite (PEA)_2_SnI_4_ possesses varied photoelectric properties under different compositions. They proposed the great potential of (PEA)_2_SnI_4_ for future multifunctional artificial neuromorphic systems. 3D schematic diagram of 2D perovskite-based synaptic device is shown in Fig. 12a with a crystal structure of (PEA)_2_SnI_4_ (in the top left corner of inset Fig. 12a). The cross-sectional view of as-fabricated devices with artificial synaptic functionalities including examples of the input light spikes and the output post-synapses current (insets) is demonstrated in Fig. 12b. Figure 12c shows the normalized post-synapse currents after several light stimulations with improved memory response by increasing the number of light spikes. Device conductivity is typically regarded as synaptic weight in artificial synaptic devices [193]. Tian et al. in 2017 reported 2D RP phase hybrid PbBr_2_ perovskite single crystals with mixed electrical and ionic transport for low-power consumption nanodevice applications and simple fabrication process [118]. They mentioned the ionic transport due to the bromide ions migration and 20-nm-diameter filaments were observed. They achieved extremely low operating current resistive memories about 10 pA, which is smaller than conventional materials. Figure 12d shows ionic transport in 2D Br-based artificial synapse with Ca^2+^ ions release and recycling process.

The device performance in terms of STP with a constant increase in peak current is shown in Fig. 12e. Figure 12f illustrates the six pulses in the second round used to generate potentiation in the short to long term at a current of about 1 nA. This result can be attributed to filament formation under constant pulses. The device's stability over the long term is reported as more than 1,000 s (Fig. 12f). Recently, in 2019 Lee and co-workers reported dimensionality-dependent plasticity in artificial synapses made of halide perovskites and used it for neuromorphic computing [194]. They reported approximately ≈0.7 fJ energy consumption per synaptic event that is very small as compared with previously reported synapses and competitive to that of biological synapses in which energy consumption is considered to be about 1–10 fJ per synaptic event. Figure 12g shows the schematic illustration of artificial synapses (left side) and device structure (right side) of a two-terminal device with ITO (bottom electrode)/ buffer-capped conducting polymer (BCCP)/perovskite layer/Al (top electrode). Figure 12h illustrates how they used repeated stimuli to produce the STP–LTP transition in the reported synapses. After 30, the loss of memory retention fitted a power function y = b × t^−m^, where m is the power function rate, t is the time duration, b is the scaling fit constant, and y is the synaptic weight [197]. The retention curve gradually declines as m drops, meaning that memory length rises, as seen in Fig. 12h inset. Another important perimeter of a synapse is paired-pulse facilitation (PPF), which is a type of STP. In the PPF, the synaptic strength progressively increases with the applied paired pulse at a small interval ∆t. The increase in PPF under two consecutive stimuli of the device is shown in Fig. 12i. Qian and co-workers reported for the first time the lead-free 2D perovskite-based flexible photoconductor and synaptic device by simple solution process method [195]. They claim the improved stability and high reproducibility of the devices at 30 mol% addition of SnF_2_ into the perovskite. Under 470 nm light, the flexible photoconductors' photoresponsivity was 16 A W^−1^ and their detectivity was 1.92 × 10^11^ Jonesrecorded and claimed to be higher than most of the similar devices. Figure 12j shows the schematic of structural diagram for (PEA)_2_SnI_4_ flexible photoconductor with PET as flexible substrate and perovskite active material under irradiation of light. The dynamic synaptic behavior of the device is mentioned in Fig. 12k. The highest value of EPSC reached by one pulse was greater than the preceding one when ten light pulses were continuously irradiated at 200-ms intervals. The EPSC drops and reaches the starting current when the light is off. This is known as mimicking a biological excitatory synapse's STP behavior.

Kim and co-workers attain multi-leveled resistive switching behavior without compliance in a fully transparent 2D perovskite and utilized it in neuromorphic computing [196]. They reported multileveled resistive switching with pulsed voltage tuning. Under applied voltage pulses, current across the device continuously flipped and reached four different values, all of which remained stable for a long period, confirming multimode RS. Possessing the ability to use a pulse input signal to regulate the device current, the analog memory device can achieve synaptic functionality. They explored the transparent 2D perovskite-based device's progressive current change characteristic in the context of pulse cycling. Normally neuron combines with the input signals to attain the threshold and produce the spike signal. Figure 12l shows the device response under 0 to + 1.3 V DC voltages pulses.

### Phototransistors and Synapse

Field-effects transistors (FETs) are the most prominent candidate to build novel and modern electronic devices for the future electronic industry. FETs having several superior properties like minimum power consumption, small noise factors, high input resistance, and ease of integration make them superior among other electronic devices. Surprisingly increasing demands of FETs with higher efficiencies and better integration densities are attaining the interest of researchers for developing modern optoelectronic devices which were not compensated by conventional metal oxide semiconductor-based FETs. The performance and efficiency of FETs mainly depend upon the selection of channels. There are several channel materials reported with improved performances, and higher responsivities but 2D perovskite-based FETs possess significantly high performances and efficiencies with extreme resistance against the short channel effect [198]. The most common methodologies to improve the working of FETs are based on the optimization of interface morphology [199], tuning of Schottky barrier height [200], and optimizing integration density by scaling down the FET into the integrated chip [201]. The density effect can be defended by the Moore’s law statement, in which he precepted that quantity of transistors will be doubled on the microchip every two years. He also proposed that the speed and capability of computers will be increased every couple of years with low cost and ease of operation.

There is extensive research in LD perovskite-based photovoltaics and light-emitting diodes but still plenty of room available for FETs and phototransistors (PTs). The exceptional charge transport properties, optoelectronic properties, and cost-effective synthesis make perovskites feasible for practical optoelectronic device applications [202, 203]. The simplest exciting example is LD perovskite-based low-cost and highly sensitive photodetectors. Normally perovskite photodetectors are classified into two main types, photodiodes and photoconductors [204]. Photodiodes with solar cell-like architecture have fast response speeds, low dark currents, and high-frequency operation ranges with low noise. Photo conductance of perovskite-based photoconductors can be modulated under incident light illumination. Phototransistor is a bipolar transistor that allows the light to illuminate its base–collector junction and generate photocurrent, flowing between emitter and collector. PTs are a special type of photoconductor with a high gain due to inherent amplification functionality by the transistors [205]. Similar to photoconductors and photodiodes, the phototransistor responds to the incident light illumination and creates excitons as well as trions in perovskite layer. When there is an external electric field present, these photoelectrons dissociate into free charges, which are gathered as photocurrents at electrodes. Another advantage of PTs over conventional two-terminal photodetectors is the modulation of input signal weak current through the gate–source voltage, permitting an improved and better sensitivity, which is not possible by two-terminal photodetectors. Like FETs, the performance of PTs is also described in terms of detectivity (D*), responsivity (R), t_rise_ and t_decay_ quantifying their interaction with light. The photoresponsivity is defined by:

$$R = \frac{{I_{{{\mathrm{light}}}} - I_{{{\mathrm{dark}}}} }}{P \times A}$$

Here I_light_ and I_dark_ represent the current under illumination, current in dark, respectively. P represents the incident light's intensity, and A is the device's active region. Similarly, D*, the detectivity, is computed by:

$$D^{*} = \frac{R \times \sqrt A }{{\sqrt {2 \times I_{{{\mathrm{dark}}}} \times q} }}$$

Here R represents responsivity, A is the device’s active area, q denotes the elementary charge. Time response of PTs is computed by t_rise_ and t_decay_, which is determined by the time it takes for the current to increase from 10 to 90% of its maximum value and drops from 90 to 10% of its highest value.

Chen al in 2016 fabricated for the first time hybrid 2D perovskite-based phototransistor. They used PEA_2_SnI_4_ thin films as 2D perovskite and achieved the responsivities about R = 1.9 × 10^4^ A W^–1^ below 447 nm illumination light owning power density 5 × 10 ^–3^ mW cm^–2^ and t_rise_ = 0.45 s [206]. Recently, Wu and co-workers introduced an innovative solid–solid conversion process that transforms unstable 3D perovskite films into highly crystalline, well‐oriented 2D configurations via amine steam treatment, significantly enhancing material stability. The resulting 2D films enable self‐powered photodetectors with narrowband light sensing, offering a promising route for the advanced manufacturing of high‐performance passive sensors [207]. Wang et al. used the same (PEA)_2_SnI_4_ perovskite material to fabricate PTs with vinylidene fluoride-trifluoroethylene as gate dielectric and attained the variation in the dielectric polarization with responsivity R = 14.57 A W^–1^, D* = 1.74 × 10^12^ Jones, and a t_rise_ (_tdecay_) of 50 ms (1.5 s) under 470 nm illumination having a light power density of 2.1 × 10^–3^ mW cm^–2^ [208]. Later on, Zhu et al. enhanced the performance of (PEA)_2_SnI_4_ PTs by improving perovskite microstructure by applying solvent engineering and reported with a light power density of 0.3 µ mW cm^–2^ and R = 1.6 × 10^5^ A W^–1^ and D* = 3.2 × 10^17^ Jones under 532 nm light, which is better than Si or InGaAs-based photodiodes [209]. 2D perovskite-based PTs with high gain and better response to the incident light illumination are used in neuromorphic visual systems. These PTs recognize as well as memorize the optical information which is important for optically driven artificial synapses. This optically driven synapse responds well to optical signals with higher gain and a much lower level of noise. Organic–inorganic hybrid halide perovskites are widely used as optically modulated synapses with a long charge carrier lifetime and high absorption coefficient. The main hurdle in these hybrid perovskites-based PTs is the difficulty to store the optical information because of rapid recombination of photo-generated carriers after the excitation of illumination.

**Fig. 13:**
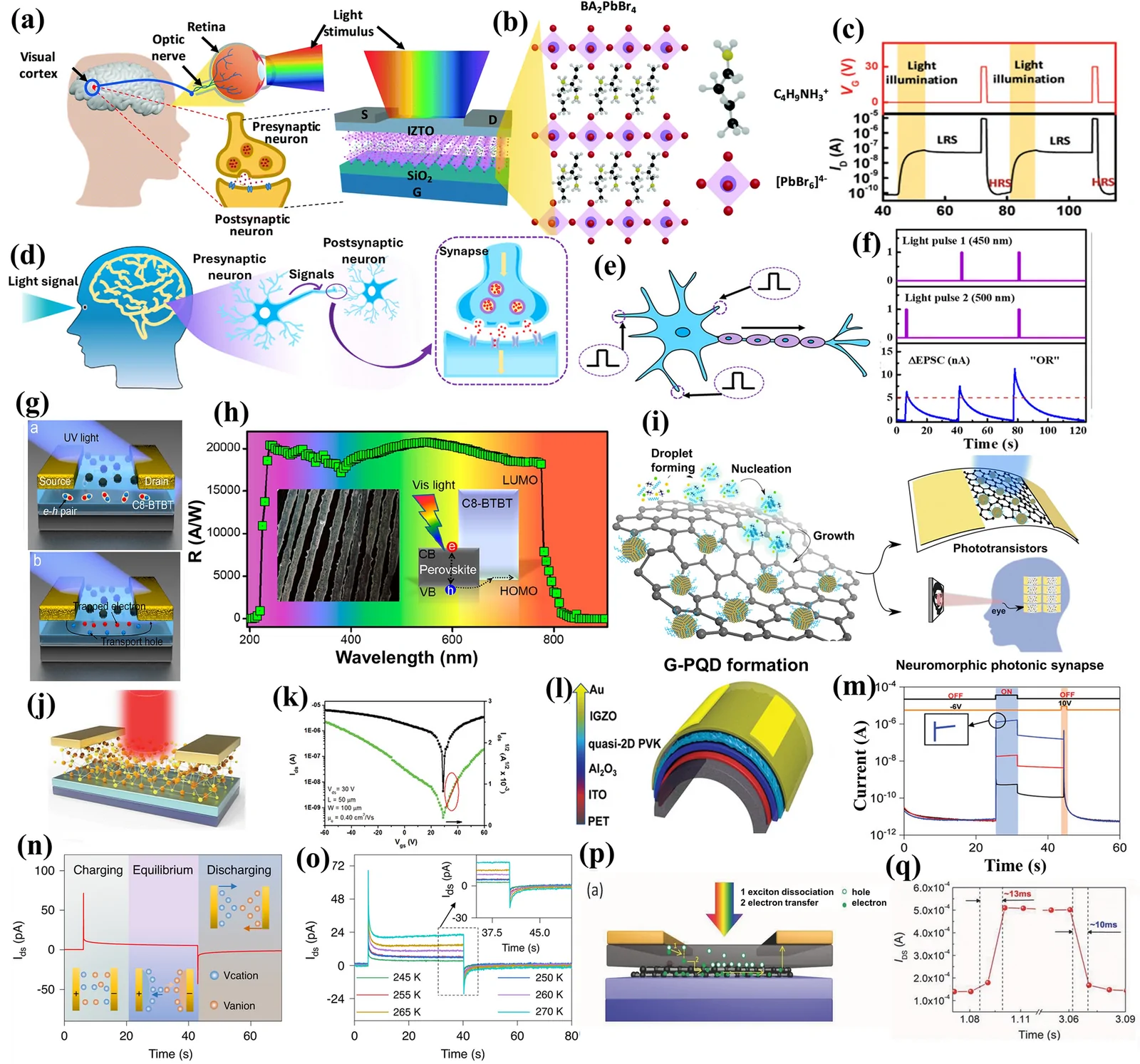
Phototransistors and synapse. **a** Schematic diagram of the human visual system and 2D perovskite-based optical synapse. **b** Structural diagram of BA_2_PbBr_4_ (where, Pb, Br, C, N, H, are represented by blue, red, black, yellow, gray, and blue colors respectively. **c** Variation in device resistance under different optical spikes [210]. **d** Schematic image of a human visual system with signal transmission between pre- and post-neurons. The right size shows the schematic of the CsPbBr_3_ QDs-based device. **e** Schematic of input and out pulse integration in a neuron. **f** Excitatory post-synaptic current curves at different input light pulses [191]. **g** Schematic of phototransistor device operation under UV illumination. **h** Photoresponsivity of the device against light wavelength, with the inset showing optically driven charge movement mechanism and another inset image of optical microscope showing C8-BTBT single-crystal array embedded with CH_3_NH_3_PbI_3_ nanoparticles [211]. **i** Schematic of the growth process of graphene perovskite superstructure (left side), device architecture (right side), and its applications in the neuromorphic photonic synapse [212]. **j** Scheme of the FET structure under light illumination. **k** Saturate transfer curves of the device with 30 V V_ds_ [213]. **l** Schematic of the flexible quasi-2D PVK/IGZO based phototransistor. **m** Writing and erasing processes of the device under infrared pulse (1064 nm). Inset showing a sharp increase in source–drain current when the gate voltage pulse is applied. The blue-shaded region represents the laser pulse [214]. **n** Schematic showing charging, equilibrium, and discharging process for Au/2D perovskite/Au device and **o** its temporal response curves under dark [214]. **p** Schematic of photogating effect in NGQDs–perovskite/mrGO phototransistor. **q** Photoswitching under light and dark at 2 s intervals with rising/fall time of 13/10 ms [215]

Recently, Lee and co-workers in 2021 overcome the carriers recombination issue by using indium zinc tin oxide (IZTO) in combination with 2D-layered metal halide perovskite to fabricate broadband optoelectronic synaptic transistors with improved performance and higher optical memory [210]. Figure 13a shows the schematic process of humans using the retina to perceive optical information. Nerve cells inside the retina initiate the transformation of optical data into neural signals. The optic nerve subsequently carries these neural signals to the brain's visual cortex. The whole process needs a huge network of neurons and synapses to process and memorize data. Figure 13a also shows the comparison of fabricated phototransistor devices consisting of 2D layered BA_2_PbBr_4_ and IZTO with the human visual system. Figure 13b shows the schematic structure of BA_2_PbBr_4_ containing octahedra [PbBr_6_]^4−^ (inorganic layer) sandwiched between two organic [C_4_H_9_NH_3_]^+^ layers where Pb, Br, C, N, H are represented by blue, red, black, yellow, gray, and blue colors, respectively. The device showed a uniform change in the resistance by transforming between high and low resistance stares under repeated applied optical pulses as shown in Fig. 13c.

Previously in 2020, Hao et al. fabricated a photonic synaptic transistor by CsPbBr_3_ perovskite quantum dots and organic semiconductors for the artificial visual system [191]. They used a simple solution-processable method to synthesize the material. They reported that the fabricated device possesses basic functionalities, including pair-pulse facilitation, the transformation between short-term and long-term memory, excitatory post-synaptic current, and “learning experience” responses. High photosensitive perovskite material combined with higher conductivity organic semiconductor showed a remarkable response at low voltage of − 0.2 V. They reported the synaptic responses at an extremely low operation voltage of − 0.0005 V. Schematic of the human visual system compared with the fabricated device is represented in Fig. 13d. Figure 13e displays the schematic diagram of dendrite integration in a neuron. Multiple pre-synaptic inputs from various dendritic sites can be combined by a neuron to elicit dynamic logic in the post-synaptic neuron. The OR logic operation is demonstrated in Fig. 13f with multiple inputs by utilizing one or two input light pulses and transforming them into a larger threshold value. Lee and co-workers applied one-step solution-processable synthesis approach to fabricate dual-band phototransistors with higher performance from hybrid perovskite as well as from organic crystal arrays [211]. They reported dual-band phototransistors with responsivity as maximum as 1.72 × 10^4^ A W^−1^ in region of 252 − 780 nm, the absorption covering the entire UV − vis range and utilized as high-security communication. Figure 13g(a) shows the device's structural diagram and working mechanism. CH_3_NH_3_PbI_3_ hybrid perovskite nanoparticles are dispersed at the C8-BTBT surface and electrons holes pair formed with in C8-BTBT when UV falls on the layer. Holes can be controlled in a very small region near BCB/C8BTBT interface by means of an applied electric field, while electrons were trapped within C8-BTBT with strong electron trapping capability; the schematic of the holes restriction and electrons trapping is shown in Fig. 13g(b). Photoresponse of the device against light wavelength at fixed light intensity is shown in Fig. 13h. The inset shows the optically driven charge movement mechanism from the hybrid perovskite layer to C8-BTBT. Another inset image of optical microscope shows C8-BTBT single-crystal array embedded with CH_3_NH_3_PbI_3_ nanoparticles. Thomas et al. used organic–inorganic halide perovskite quantum dots combined with graphene to form a superstructure providing generating and transporting charges efficiently on a single platform [212]. Perovskite quantum dots were directly grown on the active sites of graphene surface to form a superstructure. The schematic of the growth process started from droplet forming, nucleation, and growth is shown in Fig. 13i (left side). They employed these superstructures to make ultra-thin, ultra-sensitive phototransistors with photonic synaptic activity and neuromorphic computing, which they demonstrated through machine learning and facial recognition. A structural schematic diagram of the fabricated phototransistor and proposed application as neuromorphic photonic synapse is shown in Fig. 13i (right side).

Guo and co-workers fabricated ambipolar phototransistors based on CsPbBr_3_ all-inorganic perovskite microplates [213]. They reported room temperature anomalous ambipolar transport characteristics. Furthermore, they mentioned that hole mobility is light dependent, but the electron mobility is independent of incident light illumination and remains the same under various light incidences. Figure 13j shows the schematic of the fabricated FET structure under light illumination. The saturated region of CsPbBr_3_ FETs exhibits the n-type transfer curve in Fig. 13k, electron mobility of 0.40 cm^2^ V^−1^ s ^−1^ under 30 V Vds. Phototransistors and photo-induced memory operations were combinedly studied by Liao and co-workers [214]. They employed a heterostructure of indium gallium zinc oxide (IGZO) with quasi-2D perovskite to fabricate highly sensitive flexible phototransistors for broadband photodetection. They reported the photoresponsivity of the device > 10^5^ A^−1^ W at 457 nm, specific detectivity 5.1 × 10^16^ Jones, and 457–1064 nm broadband photoresponse. The schematic of flexible quasi-2D perovskite/IGZO heterostructure-based phototransistor with bottom-gate structure is shown in Fig. 13l. Apart from photodetection, they also used this quasi-2D perovskite/ IGZO heterostructure for photonic memory operations. The photoresponse of memory in terms of writing/programming and photocurrent stability is shown in Fig. 13m and inset of Fig. 13m, respectively. Jiang et al. fabricated another heterostructure with photoactive dielectric behavior consisting of Al_2_O_3_ and 2D perovskite and used them for high-performance phototransistors [214]. 2D perovskite with better availability of photogenerated carrier results in the higher photoresponsivity of > 10^8^ and 10^6^ A W^−1^ at 457 and 1064 nm, respectively. At the same time, Al_2_O_3_ facilitates the neutralization of charge trapping and ionic migration of perovskites ensuing better stability under bias voltages. A brief schematic of the charging, equilibrium, and discharging process with the movement of electrons/holes and ionic vacancies under external bias for the Au/2D perovskite/Au device is presented in Fig. 13n. The quick flow of electrons/holes along the external electric field causes a sharp increase in instant current under external bias. At the same time, the ionic vacancies accumulate at the interfaces of 2D perovskite/Au and oppose the external electric filed by forming an internal electric field, explained in Fig. 13n. Later on, the ion-induced electric field reduced to attain equilibrium condition. After removal of bias, the backward movement of ionic vacancies forms a negative current. Thermal response of devices under dark is shown in Fig. 13o.

There are several reports about low-dimensional perovskite combined with other materials to form a heterostructure and utilized as different high-performance phototransistors. Another strategy of composites formation by doping nitrogen into graphene quantum dots (NGQDs)–perovskite composite was used by Liu et al.to fabricate phototransistor with broadband detection (365 to 940 nm range), high photoresponsivity (1.92 × 10^4^ A^−1^ W), and fast response to light on–off (≈10 m) [215]. The schematic of photogating with incident light and charges movement between the hybrid channel layers is shown in Fig. 13p. Green circles indicate electrons, whereas white circles indicate holes. The extreme responsive behavior with magnified photocurrent curve under light illumination (on–off) and switching interval of 2 s is displayed in Fig. 13q; rise and fall time is 13 and 10 ms, respectively, showing the highest photoresponse rate among other graphene- or rGO-based photodetectors.

### Photodetectors

Photodetectors (PDs) are employed to transform optical signals into electrical signals. They have a wide range of applications in different areas including image sensing, optical communications, sensing applications, military applications, environmental monitoring diagnostic tools, digital radiography, and nuclear medicine, etc. The efficiency of PDs mainly relies on fast response speed, high detectivity (D*), high responsivity (R), and on/off ratio. To fabricate the higher efficiency PD, the active layers should have higher carrier mobility, absorption coefficient, better charge carrier collection, and low trap-state density. Nowadays, InGaAs-, Si-, and GaN-based photodetectors are available for commercial use. These available photodetectors have the drawback of their high manufacturing cost and low efficiency.

Researchers are striving to develop low-cost, high-efficiency photodetectors that are compatible with the CMOS technology. For decades, CMOS technologies have been employed to build mature, low-cost, and reliable integration solutions. Photodiodes and phototransistors are widely used for photodetectors. They are divided into further types like PN photodiode, PIN photodiode, and avalanche photodiode. CMOS technology has been used to fabricate PIN photodiode, avalanche photodiode, phototransistor, and a photodiode for the single-chip application. PN photodiode is very commonly used for photodetection and is very easily implemented into the standard CMOS technology. Most PDs in the UV–visible to NIR range are made up of crystalline inorganic semiconductors and their heterojunctions [216]. These semiconductors-based PDs lack in terms of complicated structures and complex fabrication requirements. Commonly used high-cost and higher temperature fabrication methods are metal–organic chemical vapor deposition (MOCVD) and molecular beam epitaxy (MBE). Meanwhile, the rigidity of the materials bounds them for flexible and large-area PDs. PDs based on solution-processed fabrication techniques are getting more attention for next-generation photosensors [217, 218]. Low-dimensional metal halide perovskites (LDMHPs) are capable candidates for optoelectronic device applications with simple solution deposition methods, weakly bound excitons, high absorption coefficient, long carrier lifetime (> 1 ms), and tunable bandgap (1.48 and 2.23 eV) [48, 83]. MAPbI_3_ and MAPbBr_3_ have Urbach energies of 15 and 13 meV, respectively, which is comparable to III–V materials (e.g., Urbach energy of GaAs is 7 meV), and LDMHPs have a significantly greater absorption coefficient than crystalline Si [219]. This small value of Urbach energy refers to the high crystallinity and minimal level of structural irregularity of LDMHP nanocrystals. At the same time, the complete exponential optical absorption edge reveals the absence of deep states within the bandgap. All these parameters make LDMHPs suitable for PDs applications for their higher optoelectronic performance [42, 220]. On a variety of substrates, 2D perovskites with single-crystalline structure can be synthesized. These single-crystalline perovskites possess better surface morphology, highly efficient photoluminescence, lower recombination rate, fewer grain boundaries, lower traps, extended carrier lifespans, and improved mechanical qualities for printed and flexible devices [221–223]. At the same time, these 2D single-crystalline perovskite materials show suppressed ion migration, better mechanical stability, phase stability, and environmental stability of final devices than polycrystalline ones. So, tremendous performances, better stabilities, and flexibility of these 2D Perovskites can produce various PDs that have not yet been noticed in polycrystalline LDMHPs. 2D MHPs are classified into layered and nonlayered MHPs. From three-dimensional MHP structures, nonlayered are directly flattened down to atomically thin films. Unlike 3D perovskites, 2D layered perovskites do not have a size restriction on the interlayer cations. Because of this structural characteristic, organic cations can reach the interlayer gap between inorganic layers. 2D layered MHPs possess better in-plane anisotropic geometry effective light absorption and quicker in-plane transmission of charge carriers due to minimum hopping barriers. 2D layered MHPs are suitable for wavelength selective narrow-band PDs, which can be widely used in machine vision, biomedical sensing, XR-imaging, and military defense systems [204, 224]. 2D layered MHPs able are able to recognize the circularly polarized light (CPL) which has potential in magnetic memory devices, optical report mechanism, display technologies, telecommunication, and chiral catalysis. All these improvements are attracting the researchers’ toward LDMHPs for PD applications. 2D perovskites with better environmental stability and tremendous optoelectronic properties are emerging as an alternative against 3D bulk perovskites. 2D perovskite-based PDs with simple and low-cost synthesis, high efficiency, wide-spectrum-range detection are significantly dominating in the optoelectronic market. Singe-crystal-based 2D perovskite PDs are more efficient in photoresponsivity as compared to film-based perovskite photodetectors due to low trap density and better stability. There are different synthesis methods for single-crystalline perovskite materials, e.g., CVD on mice and SrTiO_3_ substrate and solution-processable methods. Solution-processable methods are more prominent for the fabrication of single-crystalline structure at ambient conditions.

**Fig. 14:**
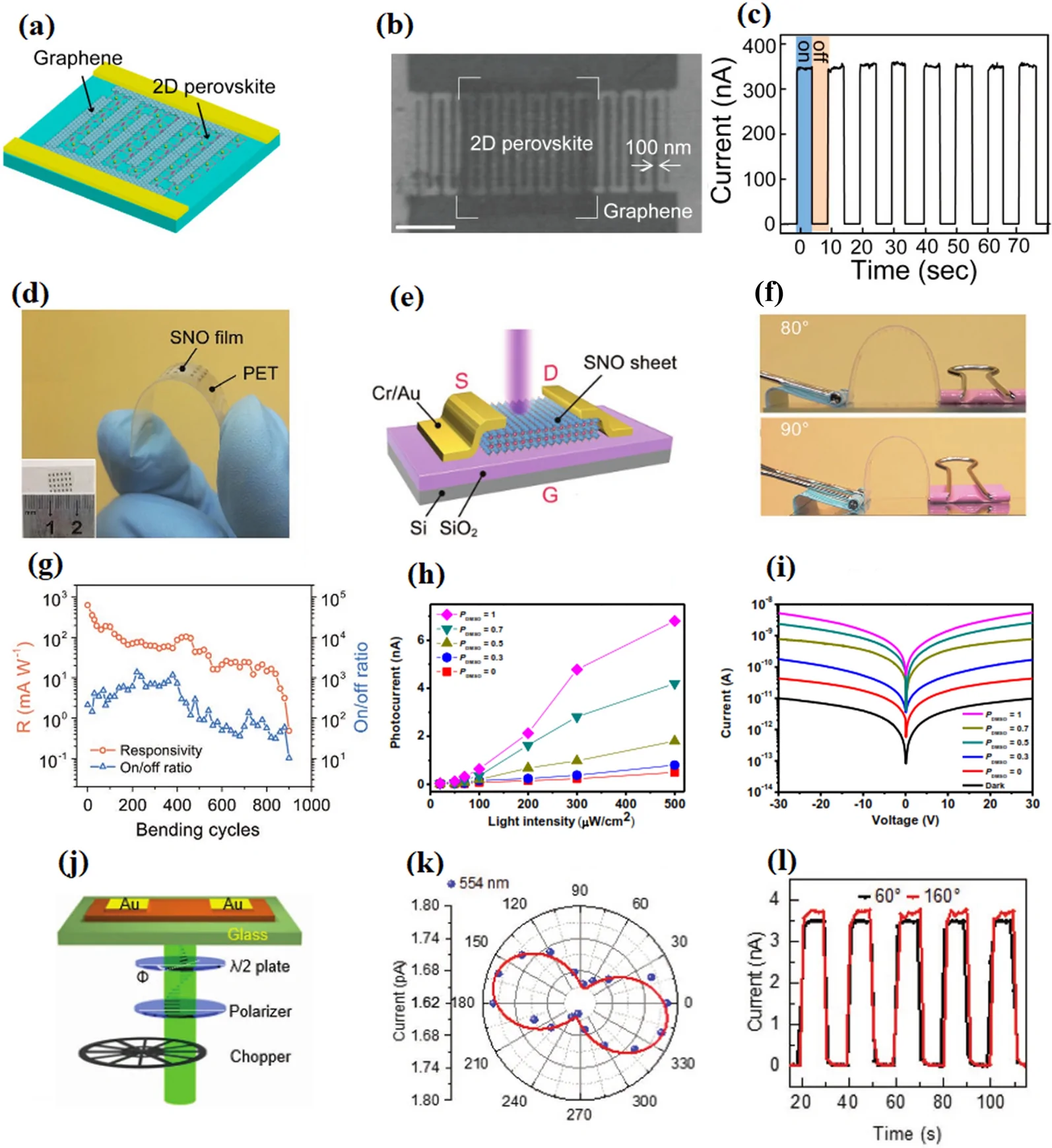
**a** Structural schematic of the fabricated device. **b** SEM showing 2D perovskite and Graphene of the fabricated device at 1 µm scale bar. **c** Current–time response of the device with a 470-nm defocused laser with 1 mm spot size, at a 0.5 V, and 10 µW bias voltage and power, respectively [22]. **d** Optical image of a flexible device on PET substrate. **e** Structural schematic of SNO sheet-based phototransistors with source, drain, and gate voltages. **f** Mechanical stability in terms of bending test at 80° and 90° degree bending angles. **g** Flexibility in terms of R_λ_ variation and ON/OFF ratio at several bending cycles [225]. **h** Photoresponse of the PD with several pure DMSO concentrations under 0–500 µW cm.^−2^ light intensities range [226]. **i** I–V response of the PD with several pure DMSO concentrations in darkness with semilogarithmic scale. **j** Schematic of device architecture and measurement setup configuration. **k** Graphs for photocurrent against polarization angle. **l** Device showing optical switch characteristics under 560 nm monochromatic light [227]

In 2016, Tan et al. synthesize (C_4_H_9_NH_3_)_2_PbBr_4_ crystals with several to tens of micrometers domain sizes by room temperature simple solution-processable technique on SiO_2_ /Si substrates and used them to fabricate photodetectors [22]. Octahedral PbBr_6_ is well connected by corner-sharing Br^**−**^ atoms to form a 2D perovskite layer in *bc*-plane. Schematic structural diagram of the fabricated device with detailed SEM image showing 2D perovskite layered pattern and graphene electrodes is shown in Fig. 14a, b. The 2D perovskite crystal under graphene functions as a light-sensitive substance and the graphene electrodes are around 100 nm in length. Figure 14c shows the photoswitching response of the device at approximately 10 on/off current ratios at a very small bias voltage (0.5 V).

Development of nontoxic and stable 2D perovskite material with a wide bandgap is a challenge. Fang and co-workers created 2D all-inorganic perovskite Sr_2_Nb_3_O_10_ (SNO)-based nanosheets with 1.8 nm thicknesses via liquid exfoliation, and they reported UV PDs based on these SNO few-layer sheets for the first time [225]. These PDs exhibit higher performance in terms of better UV detection with 1.4 × 10^14^ Jones at 270 nm detectivity, 5.6 × 10^2^% outstanding external quantum efficiency, quick response time (t_rise_ ≈ 0.4 ms, t_decay_ ≈ 40 ms), and narrowband responsivity = 1214 A W^−1^. All these characteristics are higher than any individual reported 2D sheet-based UV PDs. Figure 14d displays an optical view of a flexible device on a PET substrate. Figure 14e represents the structural diagram of SNO sheet-based phototransistors on Si/SiO_2_ substrates with incident light from upside and source, drain, and gate terminals. The flexible device's mechanical stability on a PET substrate as it is fabricated is shown in Fig. 14f at 80° and 90° degree bending angles. Bending test of flexible PD shows that PD response remains stable with Ip remaining invariant up to 80° bending angle. Figure 14g shows a sharp decline in responsivity and on/off ratio in the first 100 cycles and then constant variation with increasing bending cycles.

Grain boundaries have an important role in photodetector performance by trapping charge carriers and obstructing charge transit in perovskites. High-performance PDs are fabricated by controlling the grain boundaries of perovskite film. Lee et al. synthesized large-grained and highly crystalline films of 2D RP perovskites (C_4_H_9_NH_3_)_2_(CH_3_NH_3_)Pb_2_I_7_ by hot casting method to fabricate high-performance PDs [226]. They investigated how grain sizes and boundaries affected the performance of PDs. The concentration of DMSO and DMF is essential for film formation and grain boundaries of 2D RP perovskite thin film. Lee and fellows fabricated PDs based on several concentrations of pure DMSO and plotted the photoresponse of the PD under 0–500 µW/cm^2^ light intensities range as shown in Fig. 14h. PDs I–V characteristics depending on pure DMSO = 0, 0.3, 0.5, 0.7, and 1 under a light intensity of 300 µW cm^−2^ at 460 nm wavelength and in darkness semilogarithmic scale are shown in Fig. 14i. Li and co-workers fabricated another single-crystal 2D perovskite-based PDs, to polarization of light [227]. Previously, polarizers were combined with high cost filters to make polarization-sensitive narrowband PDs. To minimize the cost and make a simpler optical system, these single-crystal 2D perovskite-based polarization-sensitive narrowband PDs without any further sophisticated optical components were fabricated. Figure 14j shows a simple diagram of device architecture and design of the measuring setup with the polarizer on top and chopper at the bottom. It was reported that the linear dichroic ratio was 1.56 at 552 nm at an incident angle of 45° and 20 nm (full width at half maximum), that the maximum on–off ratio could reach 10^3^, that the external quantum efficiency was high at 120%, and that the peak specific detectivity was 1.23 × 10^10^ Jones under normal illumination. Using scotch tape, the crystal plates were first exfoliated to create new surfaces that would make good contact with the electrodes. The device's two typical optical switch characteristics are displayed in Fig. 14k under monochromatic light at 560 nm at two polarization angles with a bias voltage of -5 V. Photoresponse anisotropy of (iso-BA)_2_PbI_4_ single-crystal narrowband PD has been confirmed by observing a larger photocurrent for 160° than that of 60° polarization. Furthermore, as seen in Fig. 14l, the photocurrent against polarization angle response in two-lobed morphologies of the polarization-dependent photocurrent confirmed the photoresponse anisotropy.

**Fig. 15:**
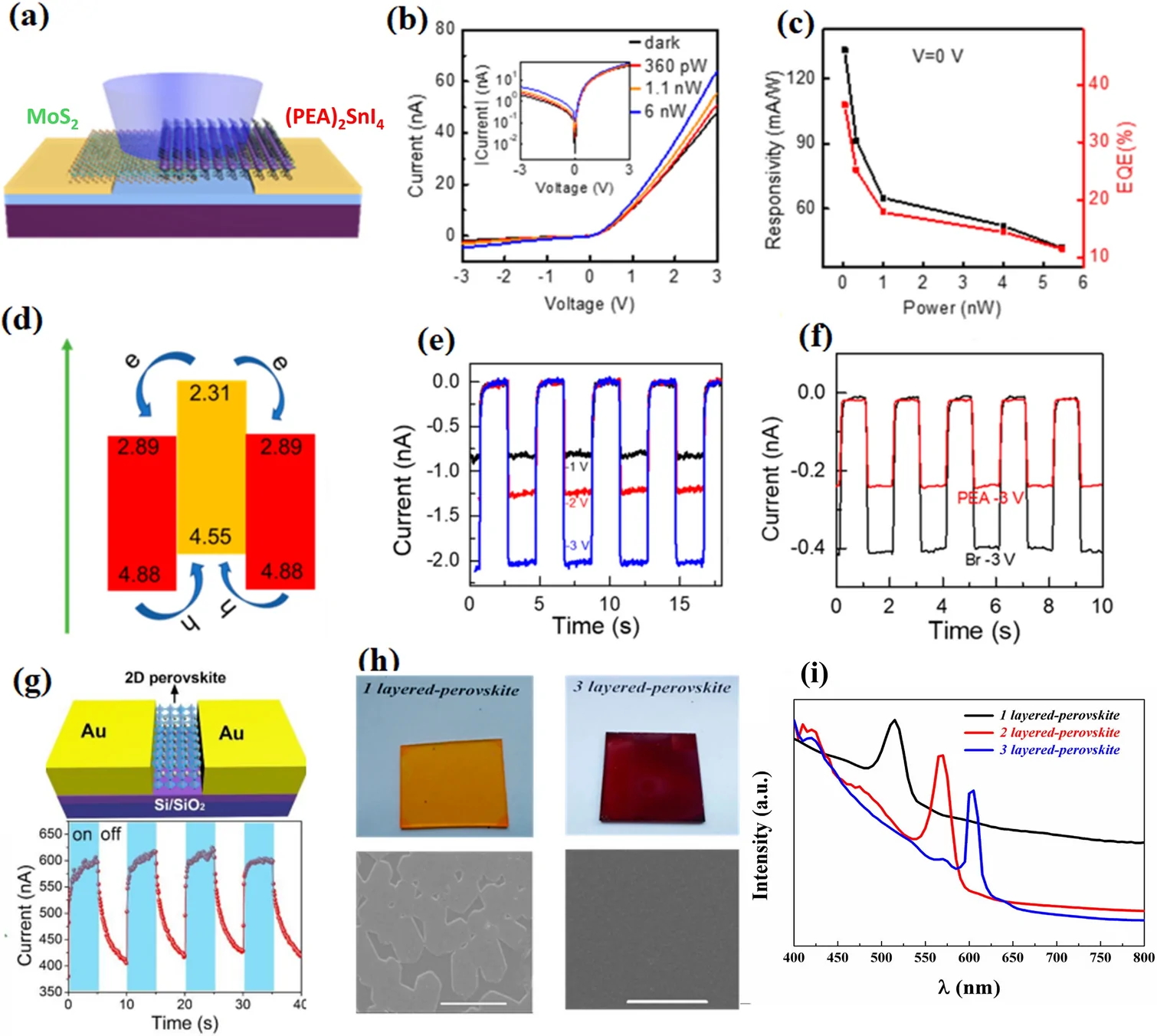
**a** Structural schematic of fabricated PD. **b** I–T plot in dark and under a 451 nm light. Inset showing logarithmic scale plots and **c** its zero bias responsivity and EQE plot [227]. **d** Band diagram of (BA)_2_PbI_4_/(BA)_2_MAPb_2_I_7_. **e** Optical switching characteristics of the heterostructural photodetector under − 1 to − 3 V applied bias and illuminated by 540 nm light with 45 mW/cm^2^ power density. **f** Optical switching characteristics of the (BA)_2_PbBr_4_/(BA)_2_MAPb_2_Br_7_ and (PEA)_2_PbI_4_/(PEA)_2_MAPb_2_I_7_ device under device at − 3 V bias voltage and under the 410 nm light (24 mW/cm) illumination and 540 nm (46 mW cm^−2^ 2) illumination, respectively [228]. **g** Structural schematic of fabricated (PEA)2SnI4 device (top image) and its I–T plot under 3 V bias voltage, 195.8 μW cm.^–2^ at 470 nm light illumination and interval of 5 s. **h** Optical images showing orange and brown colors for 1 and 3 layers-perovskite, respectively. Bottom images showing SEM images with different film morphologies for 1 and 3 layers perovskite at 5 µm scale. **i** UV–vis spectra for layer-structured perovskite materials [229]

Researchers are working to develop the stable, wide-range, and harmless 2D perovskite and their heterostructure-based PDs. n-type few-layer MoS_2_ and p-type 2-dimensional perovskite (PEA)_2_SnI_4_ heterostructures based on PD were fabricated for the first time by Li and co-workers [227]. They were able to perform light sensing over the visible and near-infrared wavelength ranges, and a tunable photoresponse peak. Figure 15a presents the diagram of the manufactured device with MoS_2_ and perovskite layers with incident light from the top side. I − V responses of the device in various light intensities and in the dark are shown in Fig. 15b. Light with 451 nm illumination and various powers intensities reflects a clear current rectification behavior with a rectification ratio of ∼15, due to the exceptional diode features in (PEA)_2_SnI_4_/MoS_2_ heterojunction. The rise of photogenerated carriers is revealed by increasing forward and reverse current by increasing light power. Logarithmic plots of I–V responses are plotted in the inset of Fig. 15b. Responsivity and EQE of the graphene/(PEA)_2_SnI_4_/MoS_2_/graphene device gradually drop by increasing incident power of the 451 nm light as shown in Fig. 15c. They reported EQE of 38.2% and the maximum responsivity of 121 mA W^−1^ were observed under 451 nm illumination with 36 pW of power.

Continuing the research on 2D perovskite heterostructures, Xiong et al. fabricated highly narrow dual-band PD based on centimeter-sized 2D perovskite heterostructures. They used the solution-processable synthesis method for (C_4_H_9_NH_3_)_2_PbI_4_/(C_4_H_9_NH_3_)_2_(CH_3_NH_3_)Pb_2_I_7_ heterostructures having better crystalline quality, controllable thickness, sizes, higher phase purity, tunable junction depth, and increased stability for thin dual-band photodetectors. They reported low dark current (about 10 − 12 A) and a very narrow dual-band spectral response, with a full width at half maximum of 20 and 34 nm at 540 and 610 nm, respectively. This improved efficiency is linked with the high crystalline purity of heterostructures. Figure 15d displays the manufactured heterostructures' band diagram, which is a type-II structure. Optical characteristics in terms of current–time plots at -1, -2, and -3 V (negative voltages) under 540 nm light illumination are shown in Fig. 15e, which shows the constant rise in photocurrent under a gradual increase in the magnitude of applied voltage. Stability and reversibility of the PD can be observed by the constant response for on and off current for all measured cycles. Furthermore, the stability and reversibility were also confirmed by the constant optical switch responses as shown in Fig. 15f. Ding and co-workers synthesized non-toxic and square-shaped microsheets of 2D Pb-free perovskite (PEA)_2_SnI_4x_Br_x_ (x = 0,1,2) by using the ternary solvent method [229]. They reported for the first time higher detectivity 2.06 × 10^11^ Jones, better photoresponsivity of 3.29 × 10^3^ A W^−1^, and a higher photo-conductive gain of 8.68 × 10^3^ of 2D lead-free perovskite microsheets-based PDs. The schematic diagram of device Au top electrodes, 2D perovskite, and Si/SiO_2_ substrates is shown in Fig. 15f upside. Constant photoswitching response at multiple cycles in terms of I–T plots under 195.8 μW cm ^−2^ illumination, 5-s on–off intervals, and 470 nm wavelength incident light is shown in Fig. 15g (bottom plot). As discussed earlier, 2D layered perovskites with quantum confinement effect and tunable bandgap can detect circularly polarized light (CPL) and can be used in magnetic memory devices, optical data mechanisms, display technologies, telecommunication, and chiral catalysis [230, 231]. All these advancements are attracting the researchers toward 2D layered perovskites-based PDs. 2D organic and inorganic hybrid perovskite materials are efficient to develop ultra-thin and fast photodetectors due to their outstanding features like high absorption, high mobility, and tunable bandgap. Many photodetectors have been reported based on organic and inorganic hybrid perovskite materials; for example, MAPbX_3_ materials-based photodetector exhibits outstanding photodetection in visible range and response speed can be suppressed by 1 ns. But restricted by the bandgap, detection of the light is only limited to the visible range. Some reports suggested that detection ability can be increased to NIR region by perovskite materials assisted with quantum dots and polymer. Huang and co-workers synthesized 2D organic–inorganic hybrid layer-structured perovskites. Optical image of C_4_H_9_NH_3_)_2_PbI_4_ (n = 1) and (C_4_H_9_NH_3_)_2_(CH_3_NH_3_)_2_Pb_3_I_10_ (n = 3) 3 layered perovskites with yellow and brown colors, respectively, is shown in Fig. 15h. SEM images of 1 layered perovskite clearly show the presence of irregular and partially discontinuous film formation which leads to insufficient light-harvesting (Fig. 15h). SEM image of 3 layered perovskite films shows better film morphology with smooth and flat surfaces. Better film morphologies with several optical band gaps of these layered materials have the potential for improved photoelectronic properties as demonstrated in Fig. 15i. UV–Vis absorption spectrum of 1, 2, and 3 layered perovskites was plotted with different characteristic absorption peaks for all three perovskites, i.e., 515, 570, and 605 nm, respectively. The corresponding optical band gaps for 1, 2, and 3 layered perovskites (2.33, 2.11, and 2.00 eV) were also reported. The difference in the optical band gaps can be attributed to different molecular structures, which directly affect the photo response of PD for multiple wavelengths of incident lights.

### High-Resolution Artificial Retina and e-Skin

**Fig. 16:**
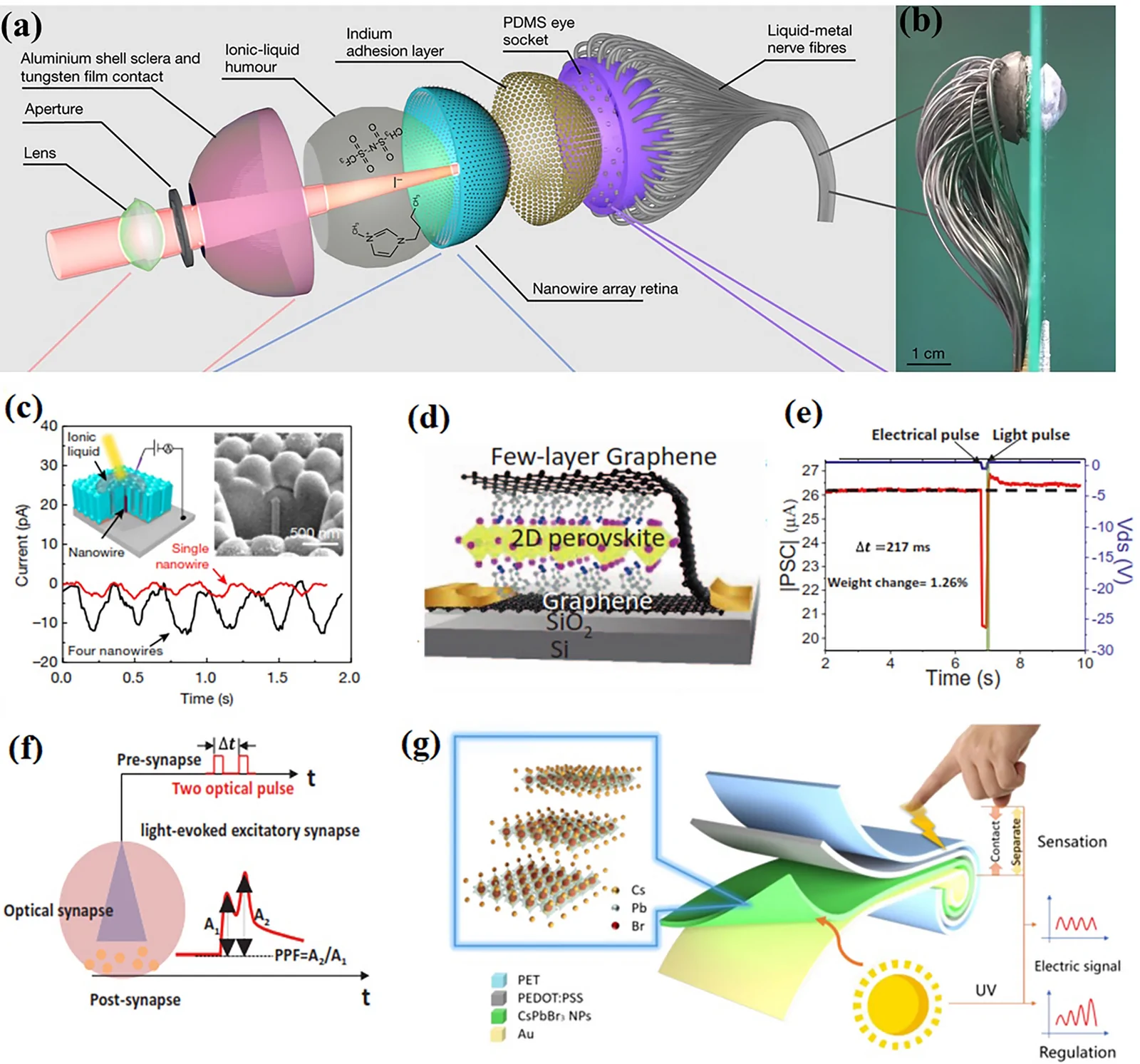
Brief schematic of EC-EYE architecture with all components **a** exploded, **b** top view and **c** its transient photoresponse [51]. **d** Schematic of the optically stimulated synaptic device consists of 2D perovskite sandwiched between graphene layers on Si/SiO_2_ substrate side as a post-synaptic terminal. **e** Synaptic response as LTP and 217 ms delay time under pre-synaptic light input and post-electrical pulse. **f** Analysis setup for optically induced synapse with two optical pulses [234]. **g** Structural schematic and working mechanism of e-skin [235]

Biological eyes are indeed the most valuable sensory organ for living species on the earth [232]. More than 80% of the information to the brain is provided by our eyes with concavely shaped retina, highest broad field of view (FOV), and ultra-high-resolution images. The advantage of the retina's domed shape is that it reduces aberration from the curved focus plane, making optical systems simpler. Artificial vision systems, which mimic human vision, are just as important in autonomous technology like robotics [233]. In particular, robots with humanoid features should have visual systems that resemble with humans, in addition to having better device qualities, to support congenial human–robot interaction. The hemispherical shape of the image sensor which resembles the human retina would be adopted to accomplish that goal. Fan and co-workers fabricated a biomimetic electrochemical eye (EC-EYE) with a hemispheric shape by using high-density perovskite nanowire retina [51]. Figure 16a shows a thorough structure of EC-EYE. In this architecture, light-sensitive, functional electrodes are made from nanowires. The counterelectrode is a tungsten (W) layer on an aluminum (Al) hemispherical shell. The cavity is filled with the ionic liquid between two electrodes, acting as an electrolyte and simulating the vitreous humor in human eye. To improve contact between the liquid metal and the nanowires, flexible eutectic gallium indium liquid–metal wires in soft rubber tubes are used to transmit signals between the nanowires and external electronics. Top view of the fabricated EC_EYE is shown in Fig. 16b. The possibility for excellent image resolution is one of the key advantages of adopting high-density nanowire arrays for artificial retina. Inset of Fig. 16c shows with a focused ion beam, a single nanowire grew in a single nanochannel, producing a single pixel with a lateral dimension of 500 nm and a footprint of 0.22 m. The graph (Fig. 16c) shows the photoresponses of 4 nanowires and single nanowire-based devices. Ren and co-workers fabricated 2D perovskite-/graphene-based highly efficient optical synapses and used them as an artificial eye [234]. Due to the huge amount of data interchange between the CPU and memory in traditional von Neumann designs, they consume a lot of power. To solve this limitation, neuromorphic computation with large-scale synapses and neurons, which can be considered a partially distributed architecture, has been proposed by Ren et al. Figure 16d shows the structural diagram of the fabricated device with 2D perovskite sandwiched between graphene layers on Si/SiO_2_ substrates. As a pre-synaptic input, a light signal is applied to the optical synapse, and an electrical pulse as a post-synaptic input is applied at the drain as mentioned in a graphical illustration (Fig. 16e). LTP occurs when the electrical signal arrives 217 ms after the electrical signal arrives and before the light pulse, i.e., 105 ms. In biosynapses, the paired-pulse facilitation (PPF) effect is a crucial short-term function that our optical synapse can replicate. Two laser pulses are delivered to at V_g_ = 10 V and V_bias_ = 500 mV, and PSC reaches maximum after the second light pulse.

Artificial skin, often known as electronic skin (e-skin), is crucial to the development of future artificial intelligence systems. Fabrication of e-skin is another important discovery toward humanoid robotics. Several attempts have been made to fabricate flexible e-skin that can detect several stimuli, but the primary stumbling block is the lack of self-regulation and learning from the environment. Kim and co-workers reported the optically induced, self-powered smart e-skin that uses photosensitive perovskite technology to achieve photoelectric neural computing and tactile sensing to enhance the human–machine interface [235]. 2D perovskite nanoplates were used to achieve the photoelectric and triboelectric effects for the functioning of e-skin. The electrode-free triboelectric concept was used to generate electrical signals from mechanical stress. The skin also works as an optoelectronic memory and possesses several synaptic behaviors. E-skin consists of a single 2 cm × 2 cm element with flexible PET substrate, PEDOT:PSS layer, CsPbBr_3_ perovskite nanoplates, and Au contacts. The sensation mechanism with optical and mechanical stimuli and heterostructure device architecture is shown in Fig. 16g.

### LD perovskite-based Photovoltaics

In the past decades, rapid population growth and huge industrial progress dramatically increased energy demand. This boom in energy demand forced people to think about clean energy alternatives to conventional fossil fuels. These replacements can be regarded as renewable and sustainable energy resources [236, 237]. Commonly used natural and sustainable energy methods include wind energy, hydraulic energy, bioenergy, and solar energy. However, solar energy is considered to be a distinguished source of electricity due to the continuous and extensive supply of energy by the Sun. Recently, photovoltaics (PV) seems to be the best solar energy harvesting technology; it directly converts solar energy into electrical energy in the most promising way to overcome the energy crises we are facing now [238, 239]. After the discovery of the photovoltaic effect in 1839 by French physicist Alexandre-Edmond Becquerel, research advanced significantly, culminating in 1954 when Daryl Chapin, Calvin Fuller, and Gerald Pearson successfully developed the first crystalline silicon (c-Si) solar cell at Bell Labs [240]. Following this breakthrough, researchers made continuous improvements in silicon-based solar cells [241–243], including enhancing the quality of amorphous silicon (a-Si) layers [244, 245] for better interface [246] and photocurrent enhancement [247, 248], replacing c-Si wafers with epitaxial silicon (epi-Si) films [249, 250], and make their real life applications [251]. However, despite these advancements, the high cost and lack of flexibility of silicon-based solar cells have limited their wider adoption in the market.

Consequently, much effort has been invested in exploring alternatives to silicon-based solar cells, such as organic solar cells (OSCs), dye-sensitized solar cells (DSCs), perovskite quantum dot-embedded solar cells, organic–inorganic hybrid solar cells, and inorganic perovskite-based solar cells (PSCs). Additionally, the incorporation of electrically harmless nanocoatings has been explored to further enhance the efficiency and durability of these alternative technologies [252–254]. Miyasaka et al. first time used organic–inorganic hybrid perovskite in 2009 for photovoltaic applications with 3.8% PCE [74]. After that in 2012, the performance was enhanced up to 9% by Gratzel and co-workers by using lead iodide perovskite-based solar cells [255]. Several research groups are working to improve the efficiencies and stabilities of perovskite-based solar cells to make them an industrially feasible candidate [256]. Alternately quantum dots embedded in perovskite are also another strategy to fabricate intermediate band solar cells [257].

**Fig. 17:**
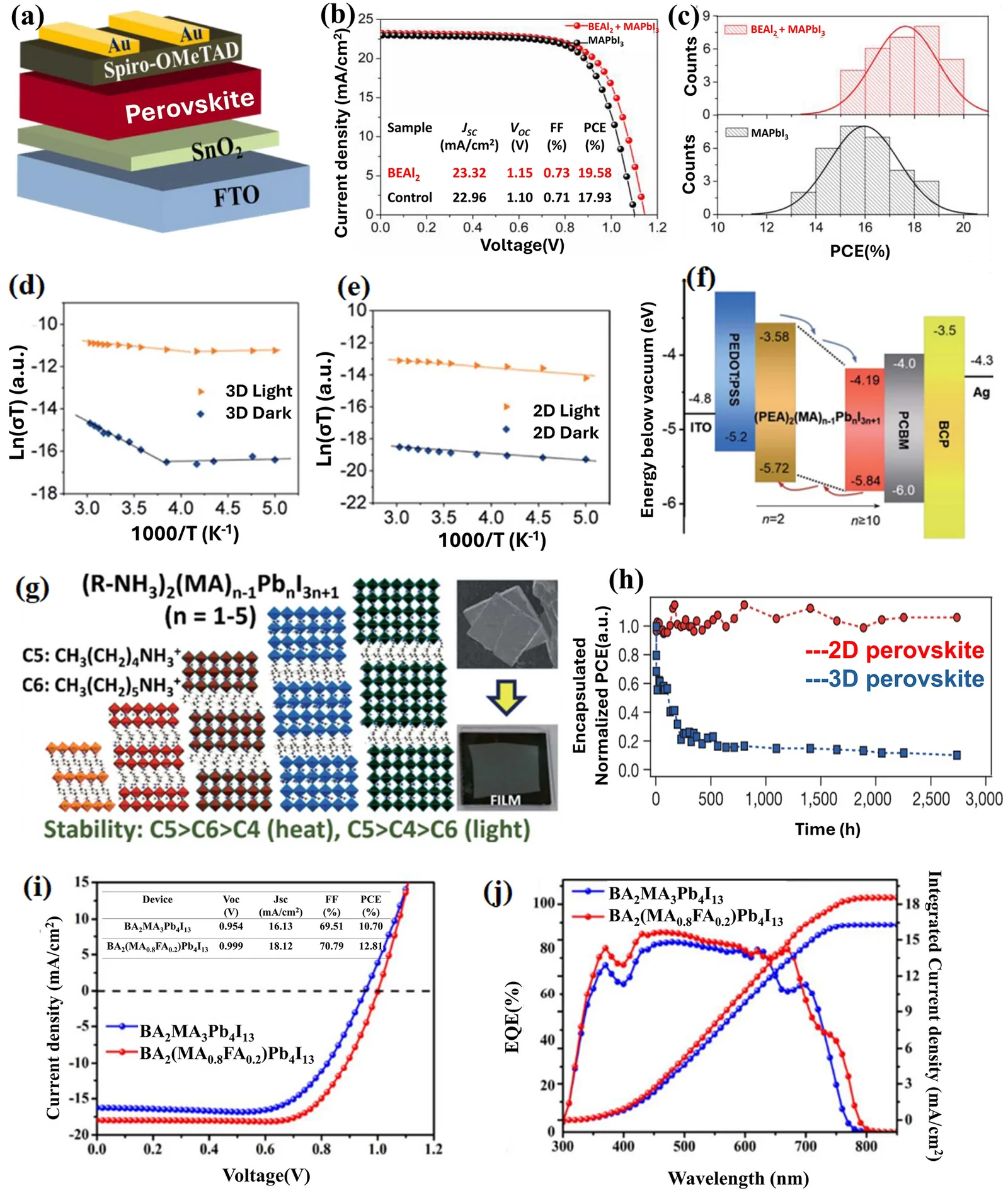
**a** Structural architecture for Au/Spiro-OMeTAD/perovskite/SnO_2_/FTO Solar cell. **b** J–V curves of PSCs with and without BEAI2 treatment. **c** Multiple devices stability against PCE for both perovskite solar cell groups [263]. Conductivity assessment at different temperatures of **d** 3D (MAPbI_3_) and **e** 2D ((BA)_2_(MA)_3_Pb_4_I_13_) films. **f** Schematic alignment for RPPs energy levels with n = 2 and n = 10, and its charge transfer mechanism [264]. **g** 2D (PA)_2_(MA)_n-1_Pb_n_I_3n+1_ family structures with increasing inorganic layer thickness from n = 1 to n = 5 [70]. **h** Stability comparison between 2 and 3D perovskite devices at continuous light irradiation for a long time (< 2,500 h) [265]. **i** J − V curve and **j** EQE response for inverted device structure of (BA)_2_(MA_1−x_FA_x_)_3_Pb_4_I_13_, x = 0, and 0.2, Q-2D perovskite-based solar cells [266]

Matteocci et al. used organic–inorganic hybrid perovskite and added a very small thickness of MoS_2_ between CH_3_NH_3_PbI_3_ and spiro-OMeTAD enhancing the efficiency of solar cells by about 13.3% [258]. In 2016, Tsai and co-workers enhanced the structural arrangement of the quasi-2D perovskite by hot casting method for a planar PCS and reported 12.5% PCE for BA)_2_(MA)_3_Pb_4_I1_3_ [259]. Due to the low-cost fabrication process and higher power conversion efficiencies (PCE), the PSC (25% PCE) and OSC (10% PCE) rapidly achieved tremendous attention from researchers [260]. Extremely high PCE and very simple fabrication process and their light management properties are making PSCs the best emerging competitor in the field of solar cells [261]. Researchers are predicting that it will beat commercial Si-based solar cells soon [262]. The structural flexibility of 2D perovskites can be utilized to tune the optoelectronic properties and make them feasible for the practical application of devices. The strong excitonic response was observed with n ≤ 2 most probably due to quantum confinement effects. At room temperature, this results in the highest photoluminescence yield, making it suitable for light-emitting applications. However, n ≤ 2 perovskites are not suitable for PVs due to their narrow absorption and high exciton binding energy; on contrary, for n ≥ 3 low exciton binding energy, smaller bandgap, and extended light absorption of perovskites in the visible region make them suitable for photovoltaic application. Figure 17a represents the structural diagram for Au/Spiro-OMeTAD/perovskite/SnO_2_/FTO solar cell. To confirm the effect of BEAI_2_ treatment on the performance of the devices, the current density–voltage (J–V) measurements were taken with and without BEAI_2_ treatment**.** Figure 17b shows the J–V curves of PSCs with and without BEAI_2_ treatment. Inset table of Fig. 17b gives complete detail about different performance parameters. The results clearly illustrate the dominant performance of BEAI_2_ with J_SC_ 23.32 mA cm ^−2^, FF 0.73, and PCE 19.58% which is complete dominant than J_SC_ 22.96 mA cm ^−2^, FF 0.71, and PCE 17.39%. The main booster in efficiency is the formation of 2D layer by post-treatment of BEAI_2_ at 3D interfaces. In addition to the performance parameters, the post-treated devices show excellent reproducibility as shown in Fig. 17c [263]. Considering more research on 2D perovskite-based solar cells, a graphical conductivity assessment comparison at different temperatures of 3D (MAPbI_3_) and 2D (BA)_2_(MA)_3_Pb_4_I_13_) films is studied in Fig. 17d, e. Schematic alignment for RPPs energy levels with n = 2 and n = 10 and its charge transfer mechanism is also mentioned in Fig. 17f [264]. 2D layered perovskites bring ease for tuning the layer thinness by adjusting n values, with a noticeable impact on perovskite structure. In Fig. 17g, the 2D (PA)_2_(MA)_n-1_Pb_n_I_3n+1_ family structures with increasing inorganic layer thickness from n = 1 to n = 5 are presented [70].

Figure 17h shows the stability comparison between 2 and 3D perovskite devices at continuous light irradiation for a long time (< 2,500 h). The graphical results illustrate the long-term stability of 2D perovskites as compared to unstable 3D perovskites. 2D perovskites-based devices are almost 80% stable after 2,500 h [265]. Zhou et al. reported (BA)_2_(MA_1−x_FA_x_)_3_Pb_4_I_13_, x = 0, and 0.2, Q-2D perovskites as light absorbers films for photovoltaics applications in the inverted architecture of ITO/PEDOT: PSS/perovskite/PCBM/BCP/Ag. In this architecture, PEDOT: PSS serves as a hole transport layer while PCBM acts as an electron transport layer. Figure 17i, j shows the J − V curve and EQE response for inverted device structure of (BA)_2_(MA_1−x_FA_x_)_3_Pb_4_I_13_, x = 0, and 0.2, Q-2D perovskite-based solar cells, respectively [266].

**Fig. 18:**
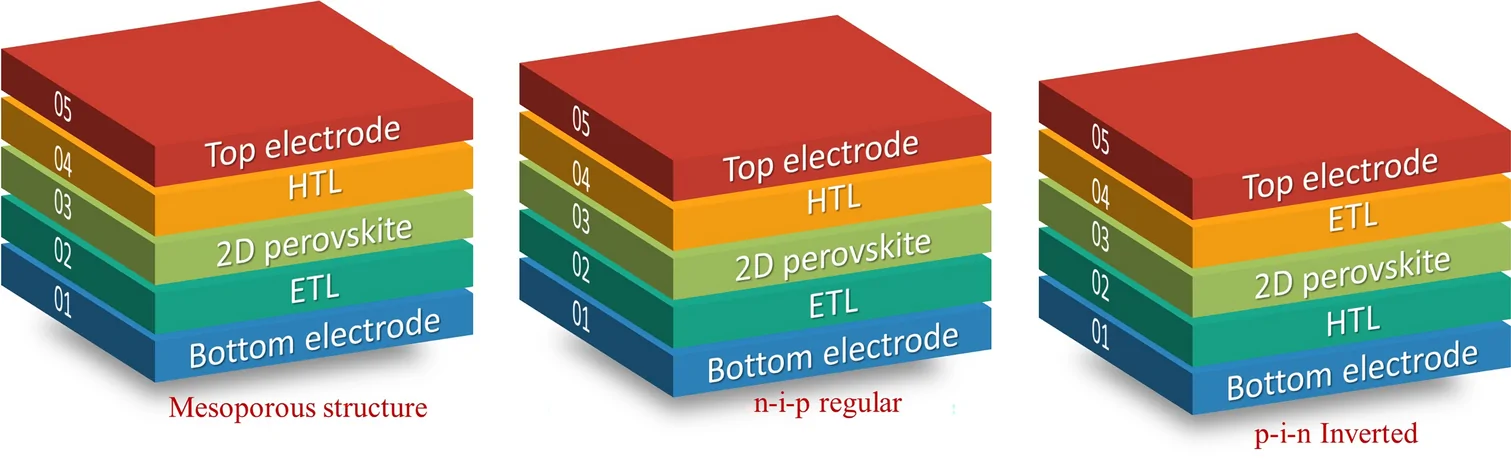
Solar cell structural configurations with **a** mesoporous, **b** n-i-p, and **c** p-i-I structures

Compared with more than 26% PCE reported in 2024 for 3D perovskite [267], the Dion–Jacobson 2D PSCs achieved the highest efficiency of 19.11%, among all other low-dimensional perovskite in 2024 [268]. Tremendous advancements in device structure [255, 269], high-quality film formation [270, 271] synthesis methods, architectural engineering of perovskite materials [260, 272] have led to boost the PCE of PSCs. The revolution in PSCs performance opened new ways to understand and enhance the PCE; one of the main approaches is the device architecture which includes mesoporous, n-i-p, and p-i-p device structure as presented in Fig. 18. These structures can be used in planner as well as inverted device structures. Table 1 shows the comparative summary of different LD perovskite material compositions, their photovoltaic performance parameters (PCE, V_oc_, J_sc_, FF), stability, fabrication methods, and device architectures.

**Table 1:** Performance and stability of various LD perovskite compositions for photovoltaic applications

Material composition	(PCE) (%)	(Voc) (V)	(Jsc) (mA/cm^2^)	(FF) (%)	Stability	Fabrication method	Device architecture	References
(BA)_2_(MA)ₙ₋₁PbₙI₃ₙ₊₁ (n = 3, 4)	12.52%	~ 1.10	15.2	~ 75	> 95% after 1655 h in N₂	Hot casting	ITO/2D perovskite/PCBM/Ag	[259]
(BA_0.5_PEA_0.5_)_2_FA_3_Sn4I_13_	8.82%	0.60	21.82	67	Improved stability	Spin-coating	Planar heterojunction	[273]
(ThDMA)(MA)_4_ Pb_5_I_16_	15.75%	~ 1.05	19.8	~ 78	> 95% after 1655 h in N₂	Solvent engineering (DMF:DMSO)	FTO/TiO₂/perovskite/Spiro-OMeTAD/Au	[274]
(4AMP)(FA)_3_Sn_4_I_13_ (DJ phase)	4.22%	0.64	14.90	44	–	4AMP spacer	ITO/TiO₂/ZrO₂/perovskite/Cr	[275]
AVA2FA_n-1_SnnI3_n+1_ (⟨n⟩ = 5)	8.71%	0.61	20.15	68	Improved stability	Spin-coating with NH4Cl additive	Planar heterojunction	[276]
(en)FASnI_3_	7.14%	0.48	22.54	66	Improved stability	Spin-coating	Planar heterojunction	[277]
FA-based quasi-2D RP (n = 5)	16.18%	~ 1.10	20.1	~ 80	High humidity/thermal stability	Antisolvent	ITO/SnO₂/perovskite/Spiro-OMeTAD/Au	[278]
(BA)_2_(MA)_3_Pb_4_I_13_ + GABr	19.3%	~ 1.15	21.0	~ 80	Improved light/thermal stability	Secondary crystallization	ITO/NiOₓ/perovskite/PCBM/Ag	[279]
(TN)FASnI_3_	5.53%	0.40	22.72	61	Improved stability	Spin-coating	Planar heterojunction	[280]
(PDA)(FA)_3_SnI_4_ (o-PDA spacer)	7.18%	0.489	20.69	71	–	o-PDA spacer	ITO/PEDOT:PSS/perovskite/DCM/BCP/Ag	[281]
(PEA)₂(FA)₆Sn₄I₁₃	5.94%	~ 0.50	18.3	~ 65	> 100 h in air (unencapsulated)	Hot casting	ITO/PEDOT:PSS/perovskite/PCBM/Ag	[282]
(GA)2FA_n-1_SnnI_3n+1_	9.60%	0.61	21.20	72	Improved stability	Spin-coating with EDA2 additive	Planar heterojunction	[283]
(PEA)_2_PbI_4_	3.2%	1.24	4.5	56	Low (degrades in humid air)	Vacuum-assisted deposition	ITO/PEDOT:PSS/(PEA)₂PbI₄/PCBM/Ag	[284]
AVA_2_FA_4_Sn_5_I_16_ (RP phase)	8.71%	0.61	21.0	68	Retained > 80% PCE after 30 days in ambient air (RH ~ 30%	NH₄Cl + 5-AVA⁺ spacer	ITO/PEDOT:PSS/Spiro-OMeTAD/BCP/Ag	[276]
(PEA)_2_FA_n-1_Sn_n_I_3n+1_ + SnF_2_	9.41%	0.61	22.00	70	Improved stability	Spin-coating	Planar heterojunction	[285]
(BA₀.₇PEA₀.₃)₂FA₃Sn₄I₁₃ (RP phase)	8.82%	0.60	21.82	67	–	Mixed BA/PEA spacers	ITO/PEDOT:PSS/Perovskite/Ca/LiF/Al	[273]
MASnI3 + 10% TEAI	6.80%	0.53	19.80	65	Improved stability	Spin-coating	Planar heterojunction	[286]
BA₂FA₃Sn₄I₁₃ (RP phase)	4.04%	0.42	23.96	40	–	BA spacer	ITO/bi-TiO₂/mp-TiO₂/PTAA/Au	[287]
(C_6_H_5_C_2_H_4_NH_3_)_24_PbI₄	2.8%	1.15	4.1	59	Moderate (stable under N₂)	Hot-casting technique	ITO/NiOₓ/(C₆H₅C₂H₄NH₃)₂PbI₄/PCBM/Ag	[288]
BDAPbI_4_ (1D lead iodide wires)	14.1%	-	-	-	95% after 1000 h	Solution processing	Not specified	[289]

### LD Perovskite-based LEDs

Low-dimensional perovskites are gaining attention for light-emitting devices because of their unique emission characteristics, which include ultra-broad, strongly Stokes-shifted luminescence with white light chromaticity [290]. Typical "sandwich" architectures are seen in LEDs, which can be classified as single-layer, double-layer, three-layer, or multi-layer structures based on the number of layers. Aside from the substrate, cathode, and anode, the emissive layer (EML) is the only component of the most basic single-layer system. Due to the imbalanced carrier transport and the quenching phenomena of the interface between the electrodes and EML, the device's efficiency is rather low despite its simple preparation method [291]. When compared to a monolayer structure, a double-layer structure increases the carrier transport efficiency and exciton recombination likelihood by adding an additional layer of either the hole transport layer (HTL) or the electron transport layer (ETL) [292]. By creating a new low-temperature reverse microemulsion technique, Zheng et al. fabricate a novel class of nano crystals (NC), which are colloidal semiconductors, by using 0D perovskite Cs4PbBr6 with an 85% yield of the reaction [293]. These 0D perovskite NCs have a superior photoluminescence quantum yield (PLQY) in both colloidal and thin-film forms (PLQY: 65% and 54%, respectively). Zhou et al. reported a 0D-tin mixed halide perovskite-based broadband yellow light emitter with high efficiency [294]. This phosphor's excited state structural rearrangement results in a significant broadband yellow emission with Stokes shift that peaked at 582 nm. It possesses a high PLQY of around 85% at room temperature and a broad full width at half maximum (FWHM) of 126 nm [294].

Cao and co-workers in 2018 fabricated for the first time low-dimensional perovskites-based LEDs by using solution process method that spontaneously form sub micrometer-scale structures; they exhibit high-brightness and efficient electroluminescence [295]. These structures can effectively extract the light from the device while maintaining electroluminescence at every wavelength and viewing angle. Amino acid additives are added to the perovskite precursor solutions to create these perovskites. These compounds can also decrease non-radiative recombination and passivate perovskite surface imperfections to improve the EQE of final devices. Xing et al. use a small ligand called isopropyl ammonium (IPA) which moderately replaces the long ligand called phenylethyl ammonium (PEA) for fabricating the quasi-2D perovskites. The process controls the crystallization of these perovskites to produce stable in color, with blue emissions perovskite thin films with high PLQYs [296]. The combined use of IPA and PEA ligands inhibited the development of the lowest-n and highest-n phases, allowing the formation of the intermediate-n phases to take center stage. The resulting films exhibit steady blue emission and extremely efficient photo luminance. Furthermore, by mixing metal halide perovskites with polymer Tian et al. fabricated perovskite-based red LEDs which are stable and extremely efficient use perovskite composite thin films as the light-emitting interface [297]. Highly luminous perovskite thin films made by varying the molar ratios of organic salts (benzyl ammonium iodide) to inorganic salts (cesium iodide and lead iodide) may emit hues that range from red to very dark red. Compared to their clean quasi-2D perovskite counterparts, thin films of quasi-2D perovskite/PEO composite have much higher photoluminescence quantum efficiencies. For these red perovskite LEDs, remarkable electroluminescence spectrum stability has been attained under continuous device operation.

Sargent et al. reported that by employing a bifunctional chemical additive that concurrently regulates the reduced-dimensional perovskites poly-dispersity and passivates the perovskite quantum well (QW) surfaces, reduced-dimensional perovskites (RDP) with a more monodispersed QW thickness distribution are created, along with a fluorinated triphenylphosphine oxide additive that creates a hydrogen bond with the organic cations, preventing the production of low-thickness QWs and regulating their diffusion during RDP film deposition [298]. By forming coordination bonds with unsaturated sites, the phosphine oxide moiety passivates the perovskite grain boundaries and prevents the production of defects. This produces RDP thin films that are compact, homogeneous, and smooth, with high photoluminescence quantum yield and narrowband emission.

**Fig. 19:**
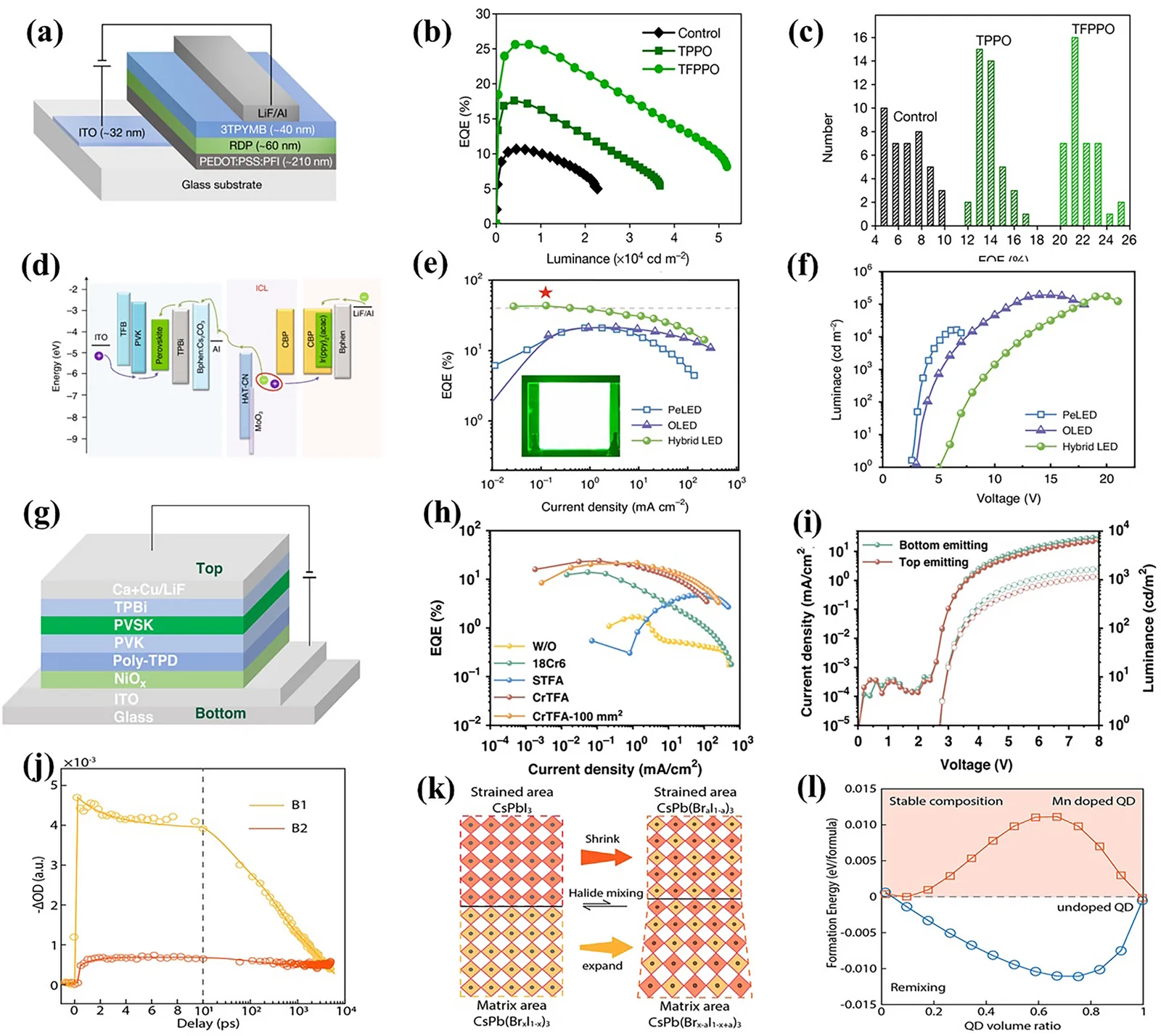
**a** LED device schematic sowing all layers. **b** Luminance-dependent EQE curves and **c** histogram of 40 devices showing the EQE of TPPO-treated and TFPPO-treated RDPs based devices [298]. **d S**chematic energy band diagram of the fabricated device. **e** EQE-J characteristics of all LEDs and maximum EQE marked by a red star. Inset shows a hybrid LED photograph at 7.5 V bias and a 30 mm × 30 mm active area. **f** Voltage-dependent luminance curves [299]. **g** Structural schematic of the device sowing all layers. **h** EQE off all devices. **i** J–V–L of both top and bottom emitting PeLED [300]. **j** Optical properties of QD-in-matrix solids. Representing kinetic traces of the B1 and B2 peaks. **k** Schematic showing transition of QD-in-matrix from a stable to a halide-remixed phase with bromide migration to strained QD. Anion exchange at the QD/matrix interface induces material strain. **l** Total free energy change (red) during the transition in **k** versus strained QD volume ratio. Free energy changes for halide mixing from unstrained iodide domains and matrix perovskite to the halide-remixed phase are also shown (blue) [301]

Figure 19a shows the schematic diagram of LED fabricated by using ITO-coated glass substrates with patterns used to make LEDs (thin-film devices) on which PDOT: PSS (poly(3,4-ethylenedioxythiophene)-polystyrene sulfonate) and PFI (nafion perfluorinated ionomer) with ratio 1:1 were deposited; then, RDPs as active layer and similarly other layers were deposited. Figure 19b shows EQE-Luminance characteristics of control, TPPO (triphenylphosphine oxide)-treated, and TFPPO (tris(4-fluorophenyl)-phosphine oxide)-treated RDP-based LEDs where TFPPO treatment RDP-based LEDs show enhanced luminance as compared to control and TPPO. Figure 19c shows that RDP-based LEDs treated with TFPPO comparatively have a very high EQE of 25.6% while TPPO-treated LEDs and control had EQEs of 17.6% and 10.7%, respectively.

Yang et al. combine the commercial organic LED along with perovskite LED to create a hybrid LED with a tandem structure that is stable, efficient, and has good color purity [299]. Perovskite and organic LEDs with a near photoluminescence peak are used to attain narrow emission spectra and maximum photon output without photon re-absorption. The green-emitting hybrid LED results from the design of an effective inter-connecting layer in addition to doping the p-type interface that reduces Joule heating and runs competent optoelectric pairing and maximum external quantum efficiency [299]. Figure 19d shows the hybrid device's energy band diagram. Due to the synchronized energy levels of the suggested ICL (inter-connecting layer), carriers were efficiently produced at the interface of MoO_3_ (Molybdenum trioxide) and CBP (4,4′-bis(N-carbazolyl)-1,1′-biphenyl)), as schematically illustrated, and inject into the top OLED and bottom PeLED under forward bias. The Al and MoO_3_ layers inside the devices have extremely small thicknesses. The thin Al layer, as opposed to an external Al electrode, makes it easier for electrons to separate and move from the ICL. The ultra-thin MoO_3_ (less than 1 nm) permits holes to pass through the spacer, which is crucial for raising the charge carrier concentration and removing more carriers. The LUMO (unoccupied lowest molecular orbital) of MoO_3_ is deeper than that of HAT-CN (1,4,5,8,9,11-hexaazatriphenylene hexacarbonitrile), but it is also lower than the HOMO (highest occupied molecular orbital) of CBP (4,4′-bis(N-carbazolyl)- 1,1′-biphenyl). This allows electrons to move from p-type interface-doped CBP to HAT-CN, enhancing the built-in electric field and enabling the creation of high-performance devices. Figure 19e shows that the luminance–voltage (L–V) graph has much higher luminance peaks of hybrid LED as compared to single sub-unit devices. Figure 19f shows the EQE-J properties of hybrid LED, PeLED, and LED where the star is highlighting the maximum efficiency value point. The hybrid LEDs are extremely efficient for generating carriers, injections, and recombination indicated by the extremely higher peak of EQE of 43.42%, which corresponds to a current efficiency that is nearly equal to the sum of the values of PeLED and OLED.

Ning et al. created a supramolecular method to control green emissive perovskites' crystallization kinetics using dual additives which slowdowns crystal growth, lowers nucleation centers, and fabricated LED with improving the efficiency nearly 21.6% also reached the external quantum yield around 23.9% [300]. Figure 19g illustrates the device's schematic, which begins with ITO-coated glass and progresses to the deposition of a NiO_x_ (nickel oxide) layer. Layers of PVK and poly-TPD are then applied to the NiO_x_ to create a stacked energy level alignment that speeds up hole injection, perovskites serve as the light-emitting layer; further TPBi (40 nm thick), LiF (1.2 nm thick), and Ca (8 nm)/Cu (4 nm) are deposited, respectively. Figure 19h shows the comparison of external quantum efficiency of various fabricated devices including without using additives, using one and more than one additive. The CrTFA (combination of STFA and 18Cr6 (18- crown-6)) device shows the lowest turn-on voltage, with effective carrier injection, low series resistance, and highest external quantum efficiency while STFA (sodium trifluoroacetate) and 18Cr6 exhibit low external quantum efficiencies because of delayed carrier transfer and high non-radiative recombination. Figure 19i displays the transparent device's current density–luminance–luminescence (J–V–L). The representative device indicates effective carrier injection from both electrodes by turning on at a low voltage of about 2.8 V. The maximum peak value of around 5.7% is obtained from the other side, whereas the ITO side's emission yields a peak of 7.9% for EQE.

Sargent et al. encapsulate strain-induced QDs as nucleation sites to create stable, bright QDs with a thin coating of perovskite precursor solution and to encourage the homogeneous crystallization of a perovskite matrix [301]. Figure 19j depicts that there are no observable emissions from the perovskite matrix in the hybrid material's PL spectrum. In the first 2 ps, the bleach recovery dynamics of B1 and B2 show that B1 recovers quickly while B2's bleach amplitude increases. Figure 19 k reveals the migration of bromide from matrix to strained QD during the change from the compositionally durable QD-in-matrix phase to the halide-remixed phase. The material experiences strain because of anions with varying atomic sizes exchanging places at the QD/matrix contact. Lattice matching is compromised when the halides are moved between the QD and the matrix. The QDs function as charge acceptors and luminophores when surrounded in a semiconducting matrix. Figure 19l shows a function of the material's strained QD volume ratio, the total free energies change (red) of the transition seen in (Fig. 19k). The halide mixing process's free energy changes are also depicted (blue) from a combination of matrix perovskite and unstrained iodide domains that have the same volume ratio as the halide-remixed phase. A table showing the comparison between different LD perovskite-based LEDs in their film morphology, PLQY, and dimensionality is presented (Table 1). It highlights the impact of different material compositions and passivation strategies on EQE and morphological characteristics. Table 2 shows a comparative overview of different perovskite compositions highlighting their external quantum efficiency (EQE), film morphology, photoluminescence quantum yield (PLQY), and structural dimensionality, along with references.

**Table 2:** Comparison of optoelectronic properties including PLQY, dimensionality, and film morphology of LD perovskite-based LEDs

Material composition	External quantum efficiency (EQE)	Film morphology	Photo-luminescence quantum yield (PLQY)	Dimensionality	References
PEA_2_Cs_16_MA_08_Pb_3_Br_10_ + TFPPO	25.6% (max), 22.1 ± 1.2% (avg)	Compact, smooth, uniform	Highest (improved by passivation)	Quasi-2D (Reduced-Dimensional Perovskite)	[298]
PEA_2_(CsPbBr_14_Cl₃)ₙ₋₁PbBr_4_ + DFBP	15.03% (max), 13.55% (avg)	Smooth, dense, uniform	64.3% (under 320 nm excitation)	Quasi-2D (optimized phases)	[302]
(PEA)_4_(S/R-PRDA)_2_-ₓSn₀.₁Pb₀.₉Br₄ chiral 2D perovskite	5.7%	Smoother surface with Sn^2^⁺ doping	–	2D (Sn^2^⁺-doped)	[303]
(TPA)_2_MnBr_4_ (TPA = tri-n-propylammonium)	–	Crystalline powder (for LED deposition)	62%	0D (isolated [MnBr₄]^2^⁻ tetrahedra separated by TPA⁺ cations)	[304]
(MBA/MPPA/MOPA)_2_Csₙ₋₁PbₙI₃ₙ₊₁	28.7% (638 nm); 22.2% (627 nm); 25.1% (645 nm)	Smooth, uniform films	> 80% (for optimized MM-MOPA films)	Reduced-dimensional (quasi-2D) Ruddlesden–Popper perovskite with MOPA ligand	[305]
Cs_3_Cu_2_I_5_	0.1%	Polycrystalline thin film	90%	0D (Isolated [Cu₂I₅]^3^⁻ clusters)	[306]
Ph_4_P)_2_MnBr_4_	7.2%	Grainy requires host matrix (e.g. TAPC)	–	0D (Isolated [MnBr₄]^2^⁻ tetrahedra)	[307]
CsCu_2_I_5_	7.4%	Densely packed, fewer defects	84.8%	1D ( [Cu₂I₅]⁻ chains)	[308]
Cs_3_CeCl_6_·3H_2_O	0.13%	Nanocrystal aggregates	~ 100%	1D ([CeCl₆]^3^⁻ chains)	[309]
(PEA)_2_SnI_4_	9.32%	Micron-sized islands → compact films	–	2D [SnI₄]^2^⁻ sheets	[310]
Cs_3_Sb_2_Br_9_	0.206%	Ligand-coated, smooth	51.2%	2D [Sb₂Br₉]^3^⁻ layers	[311]
TEA₂SnI₄ + cyanuric acid	20.29%	Reduced defects	–	2D Quantum-confined [SnI₄]^2^⁻ sheets	[312]
CsPbBr_3_ (4 nm)	97%	Uniform small nano crystals (NCs)	4.7%	Zero-dimensional	[313]
CsPbBr_3_ (3.5 nm)	94%	Small NCs	10.3%,	Zero-dimensional	[314]
CsPbI_3_ (5.4 nm)	≈100%	Small NCs	28.5%,	Zero-dimensional	[315]
CsPb(Br/I)_3_	93%	Mixed halide NCs	24.4%	Zero-dimensional	[316]
CsMnBr_3_	65.1%	Chain structure	–	One dimensional(1D)	[317]
CsPbBr_3_@Cs_4_PbBr_6_	> 90%	Nanocomposites	Enhanced stability	Core–shell	[318]

## Challenges and Perspectives

This section discusses the ongoing challenges and future prospects in memory devices and artificial synapses. It further examines recent advancements in phototransistors, photodetectors, and high-resolution retina-inspired sensors. Additionally, emerging LED technologies and innovative integration strategies are explored to overcome current limitations.

### Perspectives Memory Devices

2D perovskites are appropriate for high-performance memory devices because of their high Schottky barrier height and broad bandgap, and higher activation energy. Researchers achieved superior performances including long retention time, higher on/off current ratio, and low operating/switching voltages, long-term stability, ambient conditions synthesis, and excellent reproducibility of 2D perovskites-based memristors. These achievements reveal that 2D perovskites are more reliable compared with 3D perovskite-based devices in resistive switching memory with higher performances and long-term stability. Remarkable improvements are made in terms of device architecture and performance, but for better device design and memristive characteristics optimization, including charge trapping/detrapping and ionic transport, scientists still require a deeper knowledge of the analytical models and memristive mechanism, universal memristive properties for memory and applications of neuromorphic computing, photonics responses of memory, better controllability for conductive pathways and novel multifunctionalization for industrial applications.

### Perspective Memory and Synapse

Artificial synapses have attained remarkable attention due to their high energy efficiency, fast and reliable performances, self-learning, and massive parallel characteristics. Due to the existence of intrinsic defects, 2D perovskite has greater ion migration and charge trapping effects, making it suited for multi-level artificial synapses. It also has a lot of potential for the future development of neuromorphic computing. 2D perovskites are becoming an alternative for photo and photoelectric stimulated synaptic devices due to achievements toward better synaptic plasticity, fast learning process, long-term STP and LTP with low power consumption. 2D perovskite-based phototunable artificial synapse expands its applications toward visual simulated neuromorphic computing. Halide perovskite-based memristors are suitable for monolithic integration with silicon CMOS-integrated circuits. This will provide an opportunity to integrate nanoscale memristors with CMOS electronic circuits for advanced synaptic functions neuromorphic computing applications. Two-terminal synaptic devices perform the electronic signals transfer and self-learning process separately, while three-terminal synaptic devices are capable of both learning mechanisms and signal transmission at the same time. Considering synaptic plasticity as a fundamental principle for the human brain's learning and memory process, the simulation of an artificial synapse is desirable to achieve high-efficiency neuromorphic computing. Advanced synthesis methods for 2D perovskite with improved interface engineering methodologies and a combination with new materials for parallel processing of signals are desirable. Deep understanding of optoelectronic properties, controlled as well as uniform wafer-scale synthesis, compatibility with traditional CMOS of 2D perovskite for neuromorphic computing should be focused on the near future research.

### Perspective Phototransistors and Synapse

Even though the perovskite-based phototransistors were among early reported electronic devices, there is plenty of room to reach the level of maturity, expected for commercialization. The challenges which require immediate attention of researchers include hysteresis, low working performances, low trap density, transit frequency, and fast degradation of PTs under ordinary conditions with exposure to health, light, and moisture. Several strategies have been adopted to overcome these issues and fabricate better, efficient, and hysteresis-free perovskite-based PTs. Recently, the researchers are working to improve carrier mobility and device performance, with some strategies including interface engineering, combining heterostructure, altering buffer layer, and utilizing these higher efficiency PTs in some novel functionalities i.e., memory, synapse, etc. [319, 320]. Quantum and bandgap engineering are very hot topics in PTs research these days [321, 322]. The perovskites are advantageous over traditional semiconductors in terms of simple synthesis methodologies and better compatibility with maximum substrates. Moreover, the intermediate charge carrier movements (< 100 cm^2^ V^−1^ s^−1^) are also an advantage of these LD perovskite-based PTs. Higher working frequencies with a broad operating range are also necessary to utilize the PTs as commercial displays, wireless communication including wireless transmitters as well as receivers, military communications instruments, and fast operating computer processors. Operating voltage, channel length, contact resistance, and parasitic overlap among the gate and source/drain electrodes are also crucial factors in controlling the performances of the PTs-based synaptic devices [323–325].

LD perovskites enable us to fabricate an ultra-thin, low cost, and self-powered PTS with high efficiency and long-term stability. These achievements reveal that LD perovskites are more reliable compared with 3D perovskite-based devices in broad range operation with higher performances and long-term stability.

### Perspectives Photodetectors

Photodetectors are facing different challenges for commercialization including long-term air stability and better performances comparatively silicon-based mature technologies. Many attempts have been seen to overcome the stability issue and further enhance efficiency in the perovskite semiconductors materials by different researchers. Low trap density and controlled growth-defined thickness are two factors that may be utilized to improve air stability, high mobility, and improved efficiency in perovskite semiconducting materials. However, researchers are also trying to overcome these issues by applying different techniques, for example, interface engineering, buffer layer, sandwich device geometry, and combining heterostructure with 2D materials. The new research is focused on bandgap engineering, quantum engineering, and the deposition of novel 2D materials in the heterostructure to create efficient FETs and high-performance photodetectors. The function of bandgap engineering is to control the alloy composition during the material's growth, while quantum engineering contains heterostructure layers. 2D materials allow us to fabricate the ultra-thin, self-powered, and low-cost photodetector with high efficiency and air stability. In 2D perovskite materials, fast electron coupling at the interface results in a self-trapping phenomenon in between 2D layered perovskite materials. Self-trapping is very advantageous to adjust the electrical and optical characteristics of 2D perovskite materials. As we mentioned, several research groups have made remarkable development in exploring 2D perovskite-based PDs, but there are some challenges for device engineering, and efficiency improvement needed to be overcome. Researchers achieved the feasibility of nanosheets and nanoplatelets synthesis of perovskite, but at the same time, the morphology and thickness control remain to be improved. The thickness of nanoplatelets or nanosheets is a critical consideration for the quantum confinement mechanism to tune the optical properties of devices. Therefore, we need better synthesis techniques for improved morphology and thickness of the depositing material that has already been accomplished in traditional semiconductors and metal nanocrystals.

Despite the excellent optical properties shown by 2D perovskites, the stability of air and water remains a barrier to their broad applications. Recently, different coating like silica or Al_2_O_3_ and organic molecular capping were used to improve stability [326, 327]. However, more work is still needed to get a general method with comprehensive suitability for 2D perovskites. The presence of Pb makes lead halide more toxic than other silicon-based semiconductors, which are hazardous for humans and the environment. There have been several attempts to replace safe metal elements for lead; however, the resulting perovskites perform poorly compared to Pb-based perovskites. For example, Sn-based perovskites with bandgaps were used for better optoelectronic applications, but the oxidation of Sn^2+^ to Sn^4+^ makes them unstable compared to Pb-containing perovskites. Similarly, Ge- and Bi-based perovskites possess higher stability, but the large bandgap of these two perovskites limits their light absorption in long-wavelength range responses. Surface passivation, fabricating hybrid bilayer photodetectors, and phototransistor gating are some ways to attain improved performance and better stabilities of PDs. There is a big room to overcome the stability, performance, and toxicity issues to achieve the commercialization of 2D perovskites-based photodetectors.

### Perspective High-resolution Artificial Retina and e-Skin

To enhance the stability of LD perovskites, various coatings such as silica or Al_2_O_3_ as well as organic molecular capping have been utilized [328]. However, further research is needed to develop a generic technique that is suitable for all LD perovskites. Lead (Pb) halide is more toxic than other silicon-based semiconductors, which are hazardous to humans and the environment. Many attempts have been made to replace Pb with nontoxic elements; however, the resulting perovskites perform poorly compared to Pb-based perovskites. There are several reports about all-inorganic perovskite nanoplates showing their potential for optical information processing and advance bionic science research. This also paves the way for the future realization of neuromorphic vision and recognition. Similarly, the multifunction e-skin with mechanoreceptor and photomemory and mimicking human skin is of great interest for future research.

### Current Focus and Challenges of LD Perovskites-based Solar Cell

Despite the rapidly increasing industrial attentiveness in hybrid PSC, their commercialization is restricted to some main issues. One of the most prominent issues is device stability, an unstable device with short lifetimes is not capable to compete with the PV market requirements. The main focus of researchers is to define some basic standardized parameters for solar cell stability testing and overcome these stability issues as soon as possible to make it a commercially available device [329]. Perovskites are considered a new material in photovoltaics; they need a lot of exploratory research to make them a strong competitor in this field. Researchers are working to increase their efficiencies by optimizing the efficiency-reducing defects by altering the synthesis mechanisms correspondingly [259].

Although PSC attained rapid potential, several issues remain to be solved on an urgent basis to make them alternative to conventional PV technologies. The most critical hurdle is the very weak moisture resistance, which leads to rapid degradation of the device. Various groups are working to make a permanent solution for instability, which includes polymer-based coatings etc. Also, the presence of toxic lead in perovskite makes them environment unfriendly. Tin is used as an alternative for lead, but it has lower reported efficiencies. The addition of a new DJ phase with 13.3% cell efficiency and tremendous device stability has opened a new era of research [259, 330]. Even in extreme conditions, such as ambient air (40–70 percent relative humidity [RH]) for 4,000 h, damp heat (85 °C and 85 percent RH) for 168 h, and continuous light irradiation for 3,000 h, unencapsulated devices retain over 95 percent efficiency. This ultrahigh device stability is achieved by alternating hydrogen bonding interactions between diammonium cations and inorganic slabs, which supports the 2D layered perovskite structure [259]. Perovskite solar cells could also be used in high-performance tandem device topologies that efficiently convert ultraviolet and visible light into electricity. An additional advantage of perovskite materials is their tunable bandgap, which complement the absorption of their partner material. This can boost their efficiency and reduce costs for commercialization. The last milestone of PV is the investigation and optimization of fabrication processes [331]. A scalable and reproducible production method can be adopted and perovskite PV modules potentially exceed the office’s levelized cost of electricity (LCOE) targets [332].

One of the main challenges in LD perovskites is the photocurrent collection, which is limited due to electric field-assisted electron–hole pair separation and transport across the organic barriers. Also, the absorber layer thickness is limited to 200 nm, allowing for improved absorption and very effective photogenerated carrier separation and transport [333]. Controlling doping concentration in LD perovskites is difficult when it comes to determining the charge carrier, defect density, and band alignment in device functioning. The self-doping of single crystals of PEA_2_PbI_4_(MAPbI_3_)n1 (n = 1, 2, 3) has recently been examined as a representative application. In prototypal 2D perovskites, the femtosecond transient absorption spectroscopy is used to explore the exciton–exciton interactions by using pump-probe methods [334].

In a very short period, LD perovskites have become the alternative to 3D perovskites with better stability PSCs. Still, improving the stability of LD perovskite against moisture and air and exploring their usage in efficient and stable PSCs are the main motivation in the PV field. Although there are some improvements in LD perovskite behavior, still some crucial aspects need to be solved on a priority basis for the development of efficient commercial PSC. To accomplish well-aligned LD perovskites growth and improve the charge-transport mechanism in these materials, the next significant challenge is to establish perfect control of the material structure and thin-film morphology. Applying LD perovskite layers as barriers against water diffusion into PSCs has shown significant improvement in device stability, but improving the device efficiency is still challenging in this architecture. Cost-effective and simple synthesis methods of LD perovskites make them prominent as compared to 3D perovskite-based solar cells.

### Prospectives of LD perovskite-based LEDs

LDPs are viable contenders for the next-generation display technologies with low fabrication cost, outstanding luminous properties, including high color purity, adjustable emission wavelength, and high PLQY. External quantum efficiencies of more than 25% have been attained by green, red, and near-infrared perovskite LEDs throughout the last ten years [302]. Low-dimensional perovskites, primarily Cs_4_PbBr_6_, have lately made a comeback in a variety of nanocrystal and single-crystal forms as materials with characteristics that connect inorganic semiconductors with organic molecules. In addition to exceptional green photoluminescence with enhanced stability and high quantum yield, these characteristics include spontaneous Pb^2+^ ion emission, a significant exciton binding energy, and a tiny polaron generation following photoexcitation. The presentation of light-emitting diode devices based on Cs_4_PbBr_6_ also encourages more research into these novel materials and emphasizes the rapid efforts toward their applications as well as highlighting the development of Cs_4_PbBr_6_ perovskites, with particular attention to their molecular–electronic characteristics and the contentious green photoluminescence.

For many years, low-dimensional perovskite has been widely recognized yet because of 3D perovskite's exceptional power conversion efficiency; low-dimensional perovskites have received less attention. These materials can be synthesized utilizing a variety of organic barriers, which opens an entirely unique class of fascinating materials with enormous optoelectronic potential applications [335]. They have greater potential for LEDs than 3D counterparts because the 3D structure is constrained by the elements that can be altered in the perovskite structure, including the metal, the halide, and the small organic cation (typically methylammonium). Low-dimensional perovskite can employ organic barriers with a variety of configurations, including double bonds, aromatic rings, and chain lengths, to provide a range of optical, structural, and electrical characteristics. Additionally, low-dimensional perovskite is a major focus of future electro-luminance research because of its superior defect tolerances, adjustable emissions, exceptional purity, and enhanced stability, particularly in the presence of moisture and ultraviolet radiation.

### Current Status and Future Potential of LFHPs

Although lead-based perovskites continue to be the industry standard for efficiency in photovoltaics, with power conversion efficiencies (PCEs) in perovskite/silicon tandem systems above 33% [336], LFHPs have become a viable and environmentally beneficial option with unique benefits. Three basic LFHP restrictions are the main cause of the performance difference between these material systems: (1) Sn-based perovskites with deep-level defects localized 0.3–0.5 eV below the conduction band [337], (2) adverse band alignment at interfaces for charge transfer [338], (3) much reduced carrier mobilities (less than 10 cm^2^ V^−1^ s^−1^ as opposed to more than 100 cm^2^ V^−1^ s^−1^ in their lead-based equivalents) [339].

In tackling these issues, recent developments in two-dimensional (2D) LFHPs show impressive success. However, butylammonium copper chloride (BA₂CuCl₄) has exceptional heat stability up to 200 °C [340], while phenylethyl ammonium tin iodide ((PEA)₂SnI₄) demonstrates both remarkable moisture stability (> 1,000 h at 85% relative humidity) and strong photoluminescence quantum yield (PLQY = 82%) [341]. Double perovskite Cs₂AgBiBr₆ meets or surpasses 10^13^ Jones in specific detectivity in photodetector applications, competing with silicon-based commercial devices [110].

The performance of the LFHP device may be enhanced using three main strategies. With Sn^2^⁺/Ge^2^⁺ alloying in CsSn₀.₅Ge₀.₅I₃ which reduces trap density by 40% [342] and Mn^2^⁺ doping in Cs₂AgInCl₆ that increases PLQY from 35% to 86% [343], compositional engineering through cation substitution has shown notable improvements. Another key strategy is dimensional control, where 0D Cs₃Cu₂I₅ exhibits pure blue emission (CIE coordinates 0.16, 0.07) with 95% PLQY [344] and quasi-2D (GA)(MA)₃Pb₄I₁₃achieves 18.5% PCE with improved stability [345]. With atomic layer deposition of Al₂O₃ increasing device lifetime (T80) to 1,500 h under extreme environmental conditions (85 °C/85% RH) [346] and phenethyl ammonium iodide (PEAI) treatment minimizing interfacial recombination by 60%, interface engineering via surface passivation and encapsulation proved equally important.

Low-dimensional LFHPs have special qualities that make them ideal for applications involving visual perception. In display technologies, 2D (PEA)₂PbI₄ allows for polarized light-emitting diodes (LEDs) with remarkable anisotropy ratios that surpass 100:1 [347], while 0D Cs₃Cu₂I₅ is a powerful scintillator with light yields that reach 45,000 photons/MeV for X-ray imaging [90]. Emerging applications in AR/VR take use of the ultra-thin (< 100 nm) form factor that 2D perovskite micro-displays may achieve [348], while biomedical applications use Cs₂AgBiBr₆'s biocompatibility to produce implanted sensors [349]. These developments demonstrate how LFHPs have the potential to open new technological possibilities that are not possible with lead-based perovskites.

Moving forward, the development of LFHPs should focus on three main areas: (1) establishing robust performance comparisons through universal stability testing protocols [350], (2) building extensive structure–property databases using high-throughput screening techniques [351], and (3) developing scalable manufacturing methods like roll-to-roll processing and inkjet printing [153]. The environmental benefits and distinctive characteristics of LFHPs make them attractive substitutes for particular applications where sustainability and safety are crucial, even if there are still difficulties in matching the peak performance of lead-based perovskites.

## Summary and Outlook

The rapidly increasing demand for low-dimensional materials in smart optoelectronics has driven extensive efforts to make these materials both industrially feasible and beneficial for improving human quality of life. This demand aligns with the growing interest in portable, lightweight, low-cost, flexible, and wearable electronics. Addressing these challenges is crucial for the development of next-generation electronic devices, representing significant technological advancements in performance characteristics and a wide range of potential applications.

In this review article, we have included a list of modern fabrication processes as well as some notable applications, such as synapse, memory, photodetectors, photovoltaics, and light-emitting devices. Many new opportunities need to be addressed. Recently, there is a significant improvement in the research about low-dimensional perovskites in terms of material synthesis, stability, photonic and optoelectronic application in the last few years, but there is plenty of room available for new prospects and questions to be addressed.

1.Although there is a tremendous achievement in the fabrication techniques of LD perovskites with controlled shape and sizes as compared with conventional semiconductors, it is still very challenging.

2.LD perovskites are also continuously expanding with discoveries and inventions. For future uses of high-quality perovskite NCs, more research is needed to make them compatible with currently existing nanofabrication synthesis processes.

3.Regardless of the immense utilization of LD perovskites in several aspects, the criticism for better stability toward heat, moisture, and polar solvent with weak metal–halide bond, to achieve complete control over thin-film morphology, and their ionic nature is still challenging.

4.The fabrication techniques including surface passivation, element alteration, and physical encapsulation are widely used to make environmentally stable devices, but these strategies are limiting the performance of the devices. As a result, enhanced stability and better performance are critical for future perovskite-based devices to be commercialized.

In conclusion, LD perovskites with better tunability, ease of manufacturing, and outstanding structural properties are intriguing prospects for future electrical and optoelectronic applications.
